# Tuning the Photonic Behavior of Symmetrical bis-BODIPY Architectures: The Key Role of the Spacer Moiety

**DOI:** 10.3389/fchem.2019.00801

**Published:** 2019-12-03

**Authors:** Ainhoa Oliden-Sánchez, Rebeca Sola-Llano, Jorge Bañuelos, Inmaculada García-Moreno, Clara Uriel, J. Cristobal López, Ana M. Gómez

**Affiliations:** ^1^Molecular Spectroscopy Laboratory, Science and Technology Faculty, Physical Chemistry Department, Basque Country University (UPV/EHU), Bilbao, Spain; ^2^Laser Materials Laboratory, “Rocasolano” Physical Chemistry Institute, Department of Low-Dimension Systems, Surfaces and Condensed Matter, CSIC, Madrid, Spain; ^3^Bioorganic Chemistry Department, Instituto de Química Orgánica General (IQOG-CSIC), Madrid, Spain

**Keywords:** dye chemistry, charge transfer, excimers, lasers, BODIPY-dimers

## Abstract

Herein we describe the synthesis, computationally assisted spectroscopy, and lasing properties of a new library of symmetric bridged bis-BODIPYs that differ in the nature of the spacer. Access to a series of BODIPY dimers is straightforward through synthetic modifications of the pending *ortho*-hydroxymethyl group of readily available C-8 (*meso*) *ortho*-hydroxymethyl phenyl BODIPYs. In this way, we have carried out the first systematic study of the photonic behavior of symmetric bridged bis-BODIPYs, which is effectively modulated by the length and/or stereoelectronic properties of the spacer unit. The designed bis-BODIPYs display bright fluorescence and laser emission in non-polar media. The fluorescence response is governed by the induction of a non-emissive intramolecular charge transfer (ICT) process, which is significantly enhanced in polar media. The effectiveness of the fluorescence quenching and also the prevailing charge transfer mechanism (from the spacer itself or between the BODIPY units) rely directly on the electron-releasing ability of the spacer. Moreover, the linker moiety can also promote intramolecular excitonic interactions, leading to excimer-like emission characterized by new spectral bands and the lengthening of lifetimes. The substantial influence of the bridging moiety on the emission behavior of these BODIPY dyads and their solvent-sensitivity highlight the intricate molecular dynamics upon excitation in multichromophoric systems. In this regard, the present work represents a breakthrough in the complex relationship between the molecular structure of the chromophores and their photophysical signatures, thus providing key guidelines for rationalizing the design of tailored bis-BODIPYs with potential advanced applications.

## Introduction

Modern avenues in dye chemistry are not only oriented to the development of single fluorophores with tailor-made molecular structures (De Moliner et al., 2017) but are also focused on the rational design of multichromophoric architectures where the fluorophores are linked through covalent bonds (Ahrens et al., 2013; Fan et al., 2013). The proximity of the chromophoric subunits enables intramolecular interactions, giving rise to new photophysical phenomena ranging from a wide assortment of excitonic interactions (H- and/or J-aggregates, excimers) (Alamiry et al., 2011; Ahrens et al., 2016; Patalag et al., 2017) to charge (Zhao et al., 2013; Liu et al., 2018) and/or energy transfer (Speiser, 1996; Avellanal-Zaballa et al., 2017) processes. The balance between them or the promotion of one of them determines the final photonic performance of the multichromophoric system and, consequently, its potential field of application. It is well-established that the photonic behavior of fluorescent molecular assemblies becomes effectively modulated through a rational election of the chromophoric building blocks and the tether between them (Wang et al., 2017; Zhang, 2017; Blázquez-Moraleja et al., 2018). However, understanding and unraveling the impact of the molecular structure into the dynamics of their excited state remains a challenge owing to the complexity of multichromophoric dyes and the coexistence of several deactivation pathways competing at the same time and showing also a marked dependence on the solvent properties (Thakare et al., 2016). This knowledge becomes critical for designing straightforward and low-cost synthesis routes for smart dyes with multifunctional properties, fulfilling the tight requirements of the most advanced technological applications (Alberto et al., 2018).

**Figure 1:**
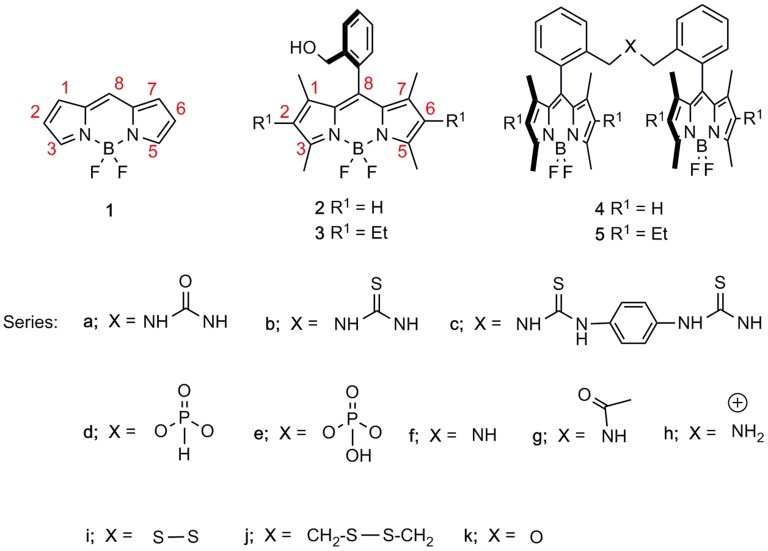
Basic molecular structure of BODIPY (**1**), our BODIPY starting materials (**2** and **3**), and all BODIPY dimers synthesized therefrom, i.e., **4** and **5**, with spacers consisting of urea-, thiourea-, phosphonate-, amine-, disulfur-, and ether-based linkers, differing in length and/or stereoelectronic properties.

Toward this aim, boron-dipyrromethene (BODIPY) scaffolds, e.g., **1** in Figure 1, are ideal candidates as building blocks owing to the chemical versatility of the chromophoric core (Loudet and Burgess, 2007; Ulrich et al., 2008). The boron-dipyrrin backbone is ready amenable to a wide range of post-functionalization routes (Boens et al., 2015), which might allow its ulterior covalent linkage to additional chromophoric units (Dumas-Verdes et al., 2010; Misra et al., 2014; Gartzia-Rivero et al., 2015; Kesavan et al., 2015; Arroyo-Córdoba et al., 2018; Xu et al., 2018; Zhang et al., 2018). Such tailoring of the molecular structure being available enables the modulation of the spectral bands of the BODIPY, leading to stable and bright dyes along the whole visible spectrum and even reaching the near-infrared region (Lu et al., 2014; Bañuelos, 2016). Multichromophoric dyes based on the BODIPY core are currently being intensively applied in various technological fields such as photovoltaics (Galateia et al., 2015), photosynthetic antennae (Ke et al., 2017), sensing and electronics (Squeo et al., 2017), electrochemistry (Qi et al., 2013), near-infrared emitters (Sakamoto et al., 2012), and as photosensitizers and labeling tools in biomedicine (Turksoy et al., 2019).

**Scheme 1:**
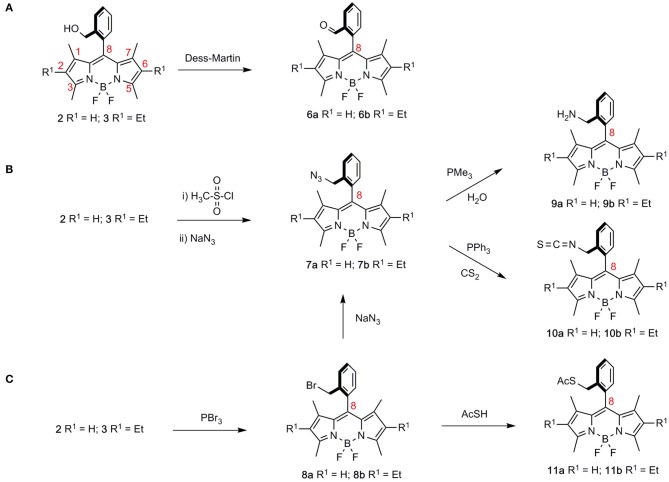
Derivatization of the hydroxymethyl group in BODIPYs **2** and **3**, leading to differently functionalized BODIPYs. **(A)** oxidation to formyl-BODIPYs **6**; **(B)** obtention of azidomethyl BODIPYs **7** as precursors of aminomethyl derivatives **9** and isothiocyanyl-BODIPYs **10**; **(C)** preparation of bromomethyl-BODIPYs **8** and their conversion to BODIPY thiolacetates **11**.

In this context, we have recently reported a straightforward synthetic approach from *ortho*-functionalized 8-aryl-BODIPYs, e.g., **2** and **3** (Del Río et al., 2017), to stable and luminescent urea-bridged symmetric bis-BODIPYs, i.e., **4a** and **5a** (López et al., 2017) (Figure 1). The fluorescence response of this covalent molecular assembly was sensible to the properties of the environment owing to the capability of the spacer moiety to induce “through-space” intramolecular charge transfer (ICT) processes. Herein, to achieve deeper insight into this ICT mechanism, which triggers the fluorescence efficiency of the whole molecular entanglement, we have synthesized a new battery of bis-BODIPYs, where the length and stereoelectronic properties of the spacer unit have been systematically modified (Scheme 1). Accordingly, the impact of the electron-releasing ability of the urea spacer was assessed by replacing the urea oxygen by a less electronegative moiety such as a sulfur atom (thiourea-bridged, i.e., **4b** and **5b**, Scheme 1). On the other hand, the effect of the distance between BODIPY subunits has been studied by incorporating a phenyl tether in bis-thiourea-derived BODIPYs, e.g., **4c** (Figure 1). Furthermore, by taking advantage of the synthetic potential of the *ortho*-hydroxymethyl group in BODIPYs **2** and **3**, an additional collection of dimers with spacers that incorporate phosphorous, i.e., **4e** and **5d**, nitrogen, i.e., **4f**, **5f**, **4g**, **5g**, and **4h**, sulfur, i.e., **4i**, **5i**, and **4j**, and oxygen, i.e., **4k** and **5k**, atoms, which differed in their electronic properties and/or the tether lengths have also been efficiently obtained (Figure 1). The computationally aided photophysical and laser study of this new set of bis-BODIPYs have contributed to the understanding of the structural controls behind the fluorescence response of these multichromophoric laser dyes.

## Results and Discussion

### Synthesis of Bridged bis-BODIPYs

**Scheme 2:**
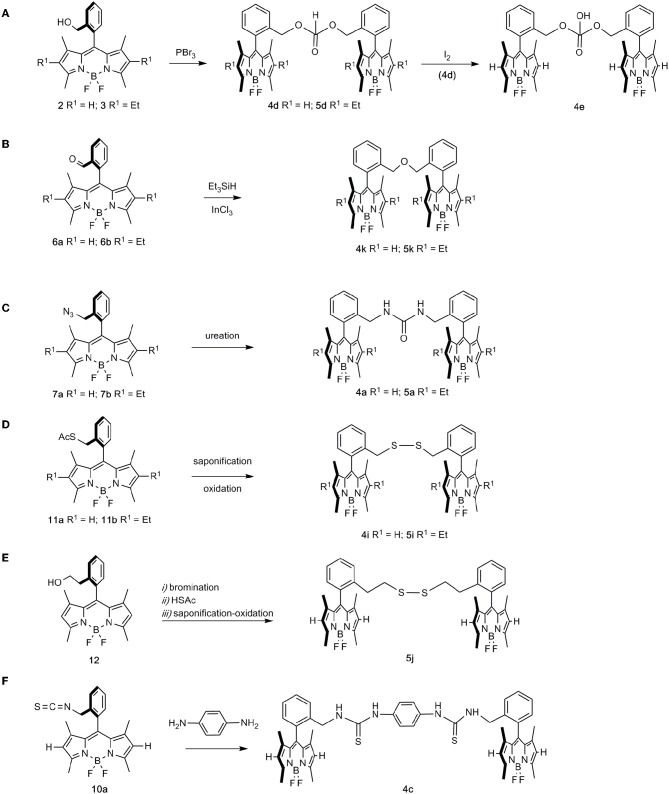
Generation of dimeric BODIPY species from single BODIPY units. **(A)** esterification of PBr3 by hydroxymethyl BODIPYs to dialkyl H-phosphonates **4d** and **5d**, followed by oxidation to phosphate **4e**; **(B)** triethylsilane-mediated reductive etherification of formyl-BODIPYs **6a** and **6b** to ether-linked dimers **4k** and **5k**; **(C)** ureation-dimerization of azidomethyl BODIPYs **7a** and **7b**, to urea-bridged bis-BODIPYs **4a** and **5a**; **(D)** saponification-oxidation on thiolacetates **11a** and **11b**, to disulfide-bridged bis-BODIPYs **4i** and **5i**; **(E)** access to “homologated” bis-disulfide **5j** from BODIPY **12**; **(F)** coupling reaction of isothiocyanyl-BODIPY **10a** with 1,4-phenylenediamine, leading to bis-thiourea derivative **4c**.

**Scheme 3:**
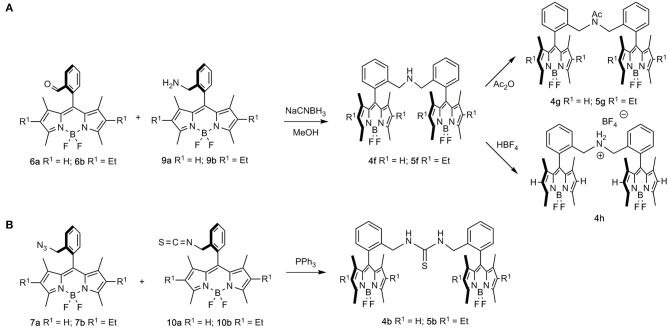
Combination of two BODIPY units leading to symmetrical bis-BODIPYs. **(A)** Reductive amination of formyl-BODIPYs **6** with aminomethyl-BODIPYs **9**, leading to bridged-aminomethyl dimers **4f** and **5f**, and their transformation to acetamido-BODIPYs **4g** and **5g** or ammonium-bridged BODIPY **4h**; **(B)** combination of azidomethyl BODIPYs **7a** and **7b** with isothiocyanyl-BODIPYs **10a** and **10b**, respectively, leading to thiourea-bridged BODIPY dimers **4b** and **5b**.

As previously mentioned, symmetric bis-BODIPYs **4a–k** and **5a–k** were prepared from BODIPYs **2** and **3**, respectively, following standard synthetic procedures that are highlighted in Schemes 1–**3** (see also Supplementary Material for detailed experimental conditions). In this context, the divergent sequences to all of the BODIPY dimers used in this study serve to illustrate the synthetic potential of *ortho*-hydroxymethyl 8-aryl BODIPYs **2** and **3**, available through a one-pot transformation from phthalide and differently substituted pyrroles (Del Río et al., 2017).

Accordingly, synthetic transformations on the hydroxymethyl group in BODIPYs **2**, **3** gave access to a “second generation” of BODIPY derivatives comprising formyl-BODIPYs **6a** and **6b** (**a** R^1^ = H, **b** R^1^ = Et, throughout the series; Scheme 1A) (Dess and Martin, 1983), azidomethyl-BODIPYs **7a** and **7b**, (Scheme 1B), and bromomethyl-BODIPYs **8a** and **8b** (Godoy et al., 2015) (Scheme 1C). The latter was also a precursor of azidomethyl BODIPYs **7a** and **7b** by nucleophilic displacement with sodium azide.

Azidomethyl and bromomethyl BODIPYs **7** and **8**, respectively, were next used as starting materials for a “third generation” of *ortho*-methyl functionalized BODIPYs, **9**–**11**. Accordingly, azidomethyl-BODIPYs **7a** and **7b** could be transformed into aminomethyl derivatives **9a** and **9b** (PMe_3_, H_2_O) or isothiocyanyl-BODIPYs **10a** and **10b** (PPh_3_, CS_2_) by way of reactions that involved BODIPY-iminophosphorane intermediates (Scheme 1B). Alternatively, nucleophilic displacement on bromomethyl-BODIPYs **8a** and **8b** with thiolacetic acid led to BODIPY thiolacetates **11a** and **11b**, respectively (Scheme 1C).

Regarding the formation of the dimeric structures, some were produced by dimerization of these BODIPY monomers (Scheme 2), whereas the rest of the dimers were obtained by a combination of two differently functionalized BODIPYs (Scheme 3).

Thus, hydroxymethyl-BODIPYs **2** and **3** were efficiently transformed into H-phosphonate-bridged bis-BODIPYs **4d** and **5d**, respectively, upon treatment with PBr_3_ (Kotlarska et al., 2013) (Scheme 2A). The oxidation of phosphonate **4d** (I_2_) (Li et al., 2014) then paved the way to phosphate-bridged bis-BODIPY **4e** (Scheme 2A). On the other hand, triethylsilane-mediated reductive etherification of formyl-BODIPYs **6a** and **6b** (Huo et al., 2018) was used in the preparation of ether-linked dimers **4k** and **5k**, respectively (Scheme 2B). Urea-bridged bis-BODIPYs **4a** and **5a** were conveniently prepared by a ureation-dimerization protocol from azidomethyl BODIPYs **7a** and **7b** (Del Río et al., 2017) (Scheme 2C). A saponification-oxidation protocol on thiolacetates **11a** and **b** allowed the synthesis of disulfide-bridged bis-BODIPYs **4i** and **5i** (Scheme 2D). Likewise, the “homologated” bis-disulfide **5j** was prepared via an intermediate thiolacetate obtained from *ortho*-hydroxyethyl BODIPY **12**, followed by a synthetic sequence related to that mentioned above (Scheme 2E).

Finally, the “elongated” 1,1′-(1,4-phenylene)-bis-(3-BODIPY-thiourea) derivative **4c**, was prepared by reaction of isothiocyanyl-BODIPY **10a** with 1,4-phenylenediamine (Scheme 2F).

Alternatively, the combination of aminomethyl-BODIPYs **9** with formyl-BODIPYs **6** (reductive-amination conditions) led to bridged-aminomethyl dimers **4f** and **5f**, which were uneventfully transformed into the corresponding acetamido- (**4g** and **5g**) or ammonium-bridged (**4h**) BODIPYs (Scheme 3A). Along this line, the combination of azidomethyl BODIPYs **7a** and **7b** with isothiocyanyl-BODIPYs **10a** and **10b** led to thiourea-bridged BODIPY dimers **4b** and **5b** (Scheme 3B).

### Photophysical Properties of Bridged bis-BODIPYs

**Figure 2:**
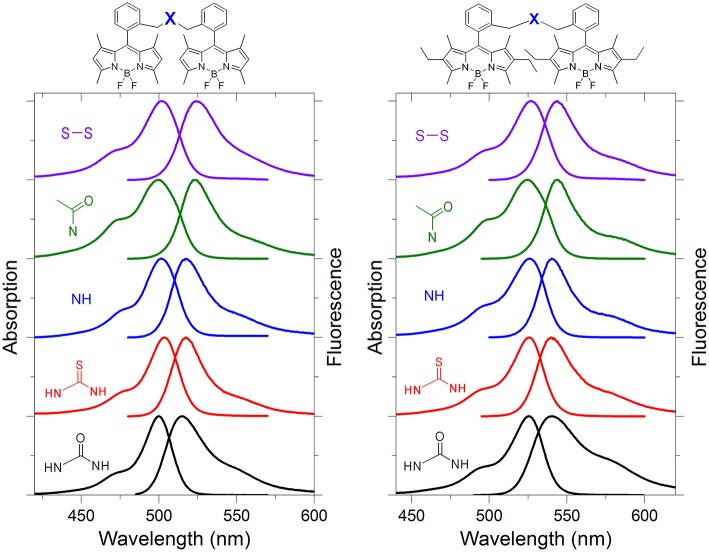
Normalized absorption and fluorescence spectra of representative bis-BODIPYs derived from building blocks **2** and **3** and linked by urea (**4a** and **5a**), thiourea (**4b** and **5b**), amino (**4f** and **5f**), acetylamino (**4g** and **5g**), and disulfur (**4i** and **5i**) bridges in an apolar environment. All the spectra in different solvents are collected in Figures S60, S61.

The conducted and joined computational-spectroscopic characterization revealed that the spacer bridging the chromophoric cores in the designed library of bis-BODIPYs played a key role in the final photophysical properties of the dyads. Therefore, hereafter, we thoroughly describe the interplay between the molecular structure and the photophysical signatures, with special attention to the fluorescence response and the ongoing non-radiative channels related to intramolecular charge transfer and excitonic couplings. Toward this aim, the photophysical properties of the new symmetric bis-BODIPYs (whose structures are shown in Figure 1) were systematically analyzed in dilute solutions (see details in experimental section Spectroscopic Properties) in polar (dimethylformamide (DMF), acetonitrile, and ethanol) and apolar (cyclohexane) solvents (Table 1; Tables S1, S2). With the exception of the ether-bridged dyads, whose particular photophysics will be discussed in detail below (section Excitonic Coupling Induced by an Ether Spacer), the spectral absorption and emission properties of all the other dyes followed a common behavior, which was also similar to that previously described for urea-based derivatives (López et al., 2017). The absorption profile of these dyads peaked at wavelengths similar to those of the corresponding single counterpart precursors (**2** and **3**, Del Río et al., 2017), while the absorption probability increased significantly (up to 23 × 10^4^ M^−1^ cm^−1^), becoming roughly twice that of each single chromophore (Figure 2, Table 1). Indeed, the theoretical simulation revealed that the absorption transition resulted from the contribution of two configurations that were energetically close (just separated by 0.03 eV), with the electronic density allocated on each dipyrrin chromophoric unit. Moreover, after molecular assembly, both BODIPY subunits were held apart (the distance between the center of masses range from merely 8 Å to around 20 Å, depending on the kind of spacer bridging the chromophores), hampering any intramolecular interaction between them. In this configuration, each BODIPY moiety was electronically decoupled, retaining its identity, and photophysical properties and contributing additionally to the global transition. Furthermore, the orthogonal disposition between the 8-aryl unit and the BODIPY core (≈90° twisting dihedral angle) owing to the steric hindrance exerted by its *ortho*-substituent and the methyl groups at C1 and C7 avoids any resonant interaction among the building blocks of these bridged bis-BODIPYs. The absorption and fluorescence spectral band positions became hypsochromically shifted by increasing the solvent polarity (Tables S1, S2), according to the behavior of the corresponding parent monomeric dyes.

**Table 1:** Photophysical properties of the bis-BODIPYs based on **2** and **3** as building blocks and linked through different bridges (urea, thiourea, phosphonate, amine, acetylamine, ammonium, and disulfur) in an apolar solvent (cyclohexane, except those not soluble, marked as ^*^, whose data are provided in diethyl ether).

	**λ_**ab**_ (nm)**	**ε_**max**_**·**10^**−4**^ (M^**−1**^ cm^**−1**^)**	**λ_**fl**_ (nm)**	**φ**	**τ (ns)**
**4a**	502.5	13.4	515.0	0.83	5.66
**4b**	503.5	10.0	517.5	0.51	1.86 (46%)−6.29 (54%)
**4c**	504.5	16.0	511.0	0.40	0.22 (31%)−1.86 (12%)−5.12 (57%)
4e^*^	500.5	13.3	514.0	0.43	0.55 (28%)−6.41 (72%)
**4f**	501.5	14.0	517.5	0.50	2.34 (56%)−6.59 (44%)
**4g**	499.0	12.3	522.5	0.89	7.51
**4h**	504.5	11.5	513.0	0.90	4.98
4i^*^	503.0	21.0	524	0.85	6.76
**4j**	503.5	14.5	518.0	0.92	6.68
**5a**	525.5	16.2	540.5	0.98	6.76
**5b**	525.5	15.5	540.0	0.76	6.79
**5d**	526.5	22.6	542.5	0.79	7.28
**5f**	526.0	15.9	540.5	0.77	7.22
**5g**	524.5	13.2	544.0	0.90	8.37
**5i**	527.0	19.6	542.5	0.84	7.04

*Full data in more solvents of different polarities are listed in Tables S1, S2. Absorption (λ_ab_) and fluorescence (λ_fl_) wavelength, molar absorption at the maximum (ε_max_), fluorescence quantum yield (φ), and lifetime (τ)*.

**Figure 3:**
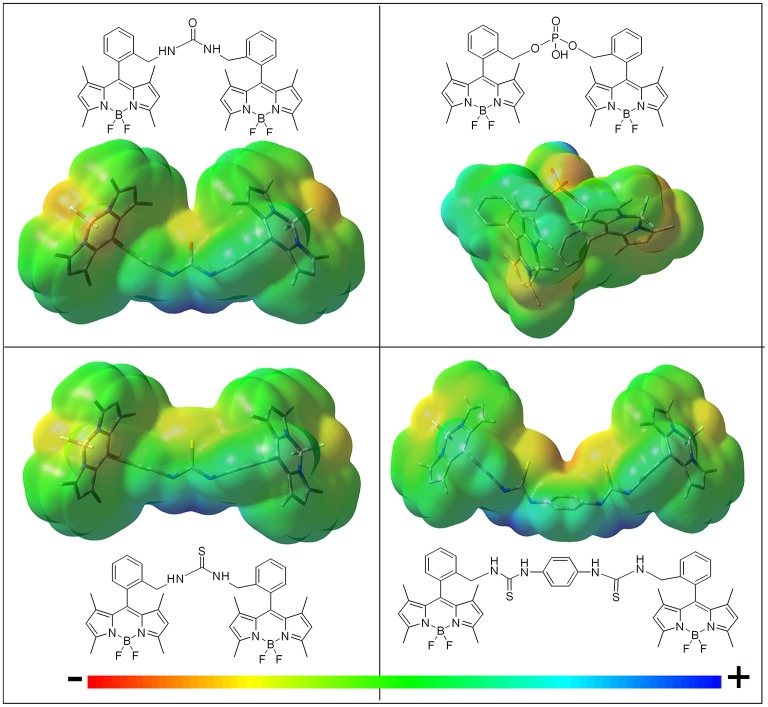
Molecular electrostatic potential (MEP) maps of the bis-BODIPYs derived from building block **2** bearing different urea-based and phosphonate-based spacers (negative charge in red and positive charge in blue). Similar MEPs are computed for the corresponding analogs built on scaffold **3**.

Regarding the emission, and owing to the claimed electronic isolation of the chromophoric units in the dyads, the high fluorescence efficiency distinctive of BODIPY dyes was retained by these bis-BODIPYs built from scaffolds **2** and **3** in apolar media (Table 1). Unlike the parent dyes, whose fluorescence was nearly solvent-independent (Bañuelos, 2016), the emission efficiency from the new dyads was markedly influenced by the solvent (discussed below in detail in sections Effect of Solvent Polarity on ICT Stabilization and The Special Case of DMF). As previously stated, while analyzing the photophysics behavior of the urea-bridged BODIPY dyes (López et al., 2017), the emission process in these dyads took place by an effective “*through-space*” ICT mechanism, with the urea spacer acting as donor unit and the BODIPY core behaving as the electron acceptor. In fact, the computed molecular electrostatic potential surfaces (MEP in Figure 3) placed remarkable negative charge at the oxygen atom of the urea bridge, showing its electron donor ability, which was even amplified by the flanking amines as well as by its proximity to the dipyrrin planes of the BODIPY skeleton. Consequently, the fluorescence emission from the herein-synthetized dyads became markedly dependent on the polarity of the media, and, even more interestingly, this solvent dependence was unambiguously modulated by the alkylation of the BODIPY core and especially by the length and the stereoelectronic properties of the spacer unit.

#### Spacer Effect on ICT Mechanism and Probability

**Figure 4:**
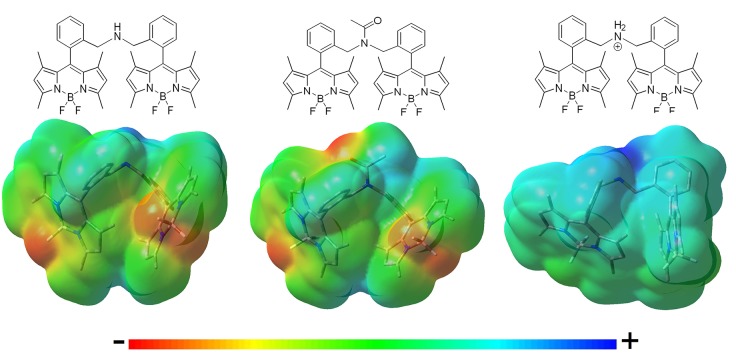
Molecular electrostatic potential (MEP) maps of the bis-BODIPYs derived from building block **2** linked by different amine-based spacers (negative charge in red and positive charge in blue). Similar MEPs are computed for the corresponding analogs built on scaffold **3**.

Actually, the photophysical signatures of the new dyads, even in an apolar solvent such as cyclohexane (Table 1), were determined by this ongoing ICT mechanism, with the spacer playing a key role:

- The higher the electron donor ability of the linker, the lower became the fluorescence efficiency. Thus, the mere replacement of the urea moiety (**4a** and **5a**) by a thiourea bridge (**4b** and **5b**) reduced the fluorescence quantum yield (i.e., from 83 to 51% in Table 1). The phosphorous-bridged dyads based on two different oxidation states (valence III in **5d** with a “pendant” hydrogen atom and valence V in **4e** with a “pendant” hydroxyl group) further supported this behavior. With respect to the urea linker, the phosphorous was less electronegative than the nitrogen, therefore increasing the negative charge on the oxygen atoms, as reflected in the corresponding MEP maps (more intense red color around the phosphonyl in Figure 3). This was especially noticeable for the spacer bearing a “pendant” hydroxyl group in dyad **4e**, where its higher electron-donor ability enhanced the ICT probability even more, leading consequently to one of the lowest fluorescence quantum yields recorded in cyclohexane (43% in Table 1).
- Following the same argument, reducing the electron-releasing ability of the spacer enhanced the fluorescence efficiency of the bis-BODIPY dyes significantly. Trying to nullify the contribution of the spacer-induced ICT, we designed BODIPY dyads with the chromophoric units linked through electronically inert moieties such as disulfur groups (**4i**, **5i**, and **4j**), which led to one of the highest fluorescence efficiencies recorded herein (up to 92% in Table 1). Similar behavior was observed on the ammonium salt-linked BODIPYdyad **4h**, which exhibited a 90% fluorescence efficiency. Thus, the ammonium salt in **4h** acted as an effective electron-withdrawing moiety according to the MEP, which located a high positive charge on the spacer (Figure 4).
- The smaller the distance between the BODIPY cores interposed by the spacer, the lower became the fluorescence performance, even reducing the electron-releasing ability of the connector. This dependence was well-illustrated by analyzing the behavior of the bis-BODIPYs linked through the shortest bridges tested herein, such as the amino-linked (**4f** and **5f)** and *N*-acetylamino-linked (**4g** and **5g)** dyads. Thus, the connection of the 8-benzyl groups of the BODIPYs through an amine group implied a shortening of the spacer length and hence the disposition of the electronic clouds of the BODIPY subunits closer than in other synthetized dyads. In spite of this geometrical arrangement, no evidence of excitonic interaction was detected in the ground state, as supported by the unaltered profile of the absorption spectra (Figure 2), but it led to an effective deactivation on the fluorescence emission (down to 50% Table 1). This trend could be understood in terms of a higher probability of the spacer-induced ICT owing to the closer proximity of the electron-donor amine to the electron-acceptor BODIPY subunits (Figure 4). Nevertheless, this drastic decrease of the fluorescence signal could demonstrate an additional pathway of non-radiative deactivation, since an ICT could also be promoted between the electronic clouds of the BODIPYs (Yu et al., 2015; Li et al., 2016) due to the mentioned proximity imposed by the amine-based spacers. This intramolecular deactivation process was what is known as photoinduced symmetry-breaking charge transfer (SBCT). According to the literature focusing on ICT processes in BODIPY dimers (Cakmak et al., 2011; Whited et al., 2012; Zou et al., 2017), the SBCT pathway has been seen to be characteristic of orthogonally disposed and directly linked BODIPY dyads, but also took place in non-orthogonal and electronically decoupled subunits through sterically hindered phenyl spacers (Liu et al., 2018). Moreover, these authors claimed that SBCT in bridged dimers did not lead to a triplet state population, as in directly linked and orthogonal dimers. In fact, no singlet oxygen generation from the triplet state of the bridged bis-BODIPYs tested herein was detected under any experimental conditions. Collectively, all these strands of evidence allowed us to state that the fluorescence deactivation in the amine-bridged bis-BODIPYs could be driven by the spacer-mediated ICT mechanism along with the aforementioned SBCT process.
- The insertion of more than one electron-donor group in the linker quenched the fluorescence more effectively, even if the connector imposed the highest distance between BODIPYs among all the structures synthesized herein. Accordingly, the increase in the number of thiourea groups at the linker on going from **4b** to **4c** reinforced the extension and effectiveness of the spacer-induced ICT process, reducing the fluorescence quantum yield from 51 to 40% (Table 1), in spite of the consequent lengthening of the linking unit, which separates the BODIPY electronic clouds further (the distance between molecular centers in the optimized geometries increased from 13.5 to 20.5 Å; Figure 3).
- Compared to the dyads arising from the C2,C6-non-alkylated BODIPY **2**, the alkylation (ethylation) at positions C2 and C6 of each dipyrrin unit promoted a further enhancement of the fluorescence efficiency [e.g., dyads linked by thiourea moieties (**4b** vs. **5b**), from 51% on the non-ethylated derivatives (**4b**) to 76% for the fully substituted (**5b**) derivatives; Table 1]. In the dyads derived from the C2,C6-diethyl scaffold **3**, all the decay curves are properly analyzed as monoexponentials. Conversely, in the bis-BODIPYs based on non-ethylated building block **2**, where the fluorescence efficiency decreased owing to the higher impact of the ICT, up to three exponentials were required to fit the corresponding decay curves (Table 1). This behavior was attributed to the inductive electron donor effect exerted by the alkyl moieties grafted to the BODIPY, which decreased the electron-withdrawing ability of the chromophoric core and consequently hampered the ICT probability from the corresponding spacer.

### Effect of Solvent Polarity on ICT Stabilization

**Figure 5:**
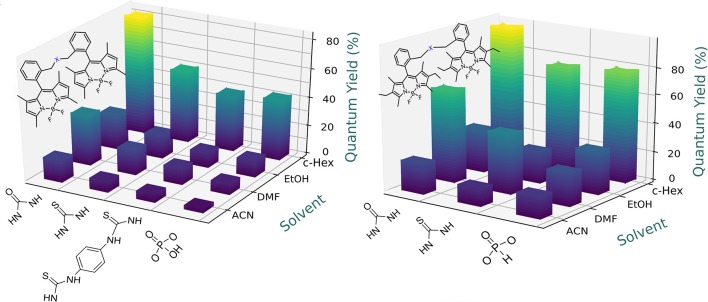
Dependence of the fluorescence efficiency on the solvent polarity for the urea-based and phosphonate-based bridged bis-BODIPYs built on chromophoric scaffolds **2 (Left)** and fully alkylated **3 (Right)**. Full data are reported in Tables S1, S2.

**Figure 6:**
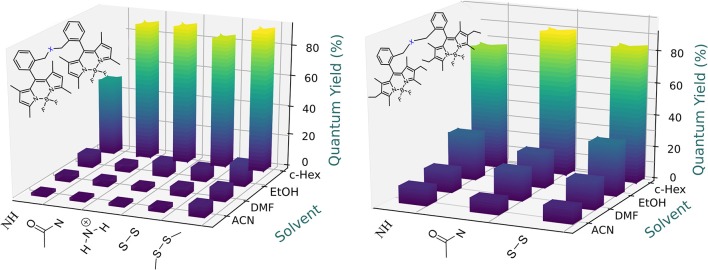
Dependence of the fluorescence efficiency on the solvent polarity for the amine- and disulfur-based bridged bis-BODIPYs built on chromophoric scaffolds **2** (left) and **3** (right). Full data are reported in Tables S1, S2.

Owing to the capability of the spacer moieties to induce effective ICT processes, especially in combination with the electron-withdrawing character of the BODIPY core, the fluorescence emission of the new dyads depended markedly on the solvent polarity (Figures 5, 6). Without exception, an increase of the solvent polarity led to a drastic decrease in the emission efficiency of all the dyads, so that in the most polar solvents such as acetonitrile, these systems could be considered as non-fluorescent, with quantum yields as low as 2%. Moreover, the fluorescence-quenching correlated with a drastic change in the fluorescence decay curves, since the time-resolved emission profile acquired a multi-exponential character, with the contribution of the shorter-lived component gaining prominence as the solvent polarity increased (Tables S1, S2). This trend also became modulated by the electron donor character of the molecular structure of these dyads. As we mentioned above, the full alkylation of the BODIPY core reduced its electron-withdrawing character, thereby weakening the solvent-sensitivity of the fluorescence emission (Figures 5, 6). As a matter of fact, the fluorescence quantum yield of the thiourea-bridged bis-BODIPYs derived from **2** (i.e., **4b**) decreased from 51% in cyclohexane to just 8% in a more polar media such as acetonitrile, while a similar dyad derived from the fully-alkylated scaffold **3** (i.e., **5b**) retained an efficiency of 13% in the same polar solvent. Likewise, two structural factors imposed by the spacer moiety enhanced this fluorescence deactivation: (i) a further increase of the electron donor ability of the spacer, for instance, the emission quantum yield in acetonitrile on going from urea-bridged dyad **4a** to double thiourea-linked bis-BODIPY **4c** decreased from 16% to just 6% (Figure 5), and (ii) a shortening of the spacer bridge, activating both charge transfer mechanisms mentioned above (ICT and SBCT). Thus, the lowest fluorescence quantum yield in acetonitrile was recorded from amine-bridged bis-BODIPY dyads (2% for **4f** and **4g** arising from BODIPY **2**, and ≈10% for **5f** and **5g** derived from BODIPY **3;** Figures 5, 6).

#### The Special Case of DMF

To obtain additional insight into the solvent-sensitive fluorescence of these dyads, we also analyzed their photophysical signatures in an electron-donating solvent such as DMF. Owing to high polarity of DMF [described by the Catalán polarity solvent scale (Catalán, 2009) as SdP = 0.977, similar to that of acetonitrile, 0.974], low fluorescence efficiency and a bi-exponential decay curve, dominated by a short lifetime component, should be expected as result of a further stabilization of the ICT process. However, the bis-BODIPYs obtained from both **2** and **3** skeletons and based on urea (**4a** and **5a**), thiourea (**4b** and **5b**), and double thiourea (**4c**) linkers exhibited fluorescence quantum yields higher than those recorded in less polar solvents like ethanol (Figure 5). This unusual behavior prompted by DMF should be related not only to its polarity but also to its electron-donor ability [basicity scale (Catalán, 2009) SB = 0.613], the highest among the solvents selected herein. The basicity of DMF could induce specific interactions between this hydrogen-bond-acceptor solvent and the proton of these linkers so long as the electron lone pair of the latter was mainly located on the amine moiety and less shifted toward the oxygen atom. In agreement with this, the positive charge (see the blue color in Figure 3) was mostly located around the nitrogen atoms of the spacer, highlighting this position as the most suitable for interaction with electron donor solvents. These interactions must have decreased the electron donor capacity of the urea-based bridges and, hence, have efficiently hampered the probability of the ICT process. Therefore, the fluorescence recorded in DMF arose from the balance between two opposite effects: the intrinsic polarity-induced stabilization of the charge separation counterbalanced by the high basicity of DMF. This last specific interaction hindered the ICT population and yielded higher fluorescence efficiencies and larger lifetimes than those expected in this polar solvent (Tables S1, S2). Two further experimental trends confirmed this hypothesis: on the one hand, the enhancement of the fluorescence efficiency induced by DMF decreased in the bis-BODIPYs grafted by urea > thiourea > double thiourea bridges (Figure 5) and, on the other hand, this enhancement was no longer recorded with the other spacer moieties selected herein (Figure 6). In these latter dyads, the corresponding fluorescence quantum yield decreased according to the polar character of the solvent once the specific interaction of the DMF with the spacer was no longer taking place, probably due to the absence of ionizable hydrogen atoms flanking the spacer moiety, as happened in the urea-based linkers.

### Excitonic Coupling Induced by an Ether Spacer

**Figure 7:**
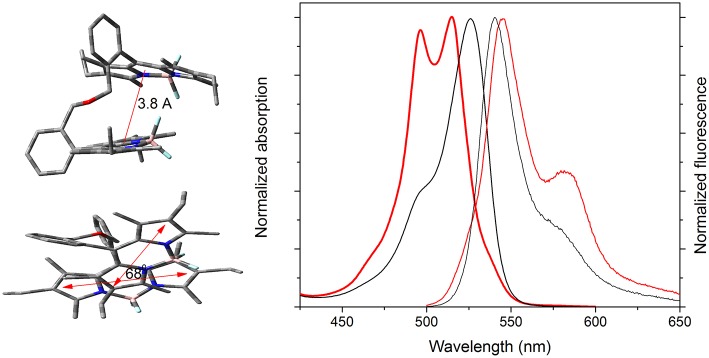
Normalized absorption (thick line) and fluorescence (thin line) spectra of the ether-bridged bis-BODIPY **5k** and its corresponding amino-bridged analog **5f** (black line) in cyclohexane. The optimized ground state geometry of the former bis-BODIPY is also included in different views to highlight the feasible excitonic coupling between the transition dipole moments oriented along the longitudinal molecular axis.

**Figure 8:**
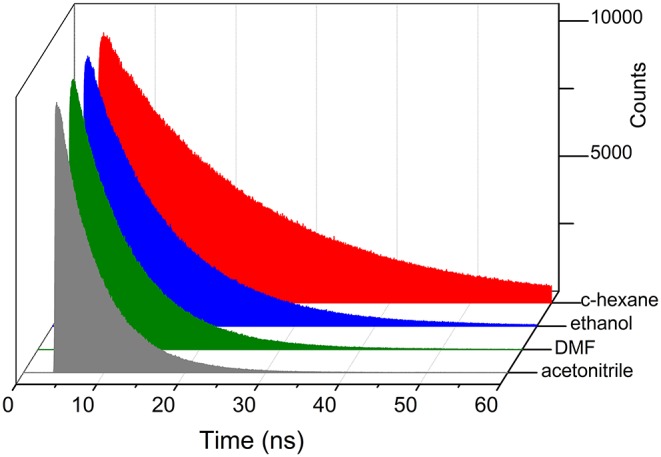
Fluorescence decay curves of **5k** with increasing solvent polarity.

The absorption profiles of the ether-bridged bis-BODIPYs (**4k** and **5k**) were noticeably different with respect to those recorded from their analog amine-bridged dyads (Figure 7, Figure S1). Regardless of the solvent, the absorption spectrum was hypsochromically shifted with respect to the rest of the bis-BODIPYs and also split into two peaks of similar intensity (Figures S60, S61). This deep disruption of the absorption profile pointed to intramolecular excitonic interaction between the BODIPY subunits. Indeed, the optimized geometry (Figure 7) revealed that the oxygen hybridization left the chromophoric subunits very close together (around 3.8 Å) and almost in a twisted cofacial arrangement (a dihedral angle between the transition dipole moments of 68°). This geometrical arrangement could promote excitonic interactions between the BODIPY electronic clouds (see the feasible overlap between one pyrrole and the central ring of the other BODIPY, Figure 7). According to the exciton theory, the growth of new absorption bands at higher energies is indicative of head-to-head interactions between the transition dipole moment (intramolecular H type aggregate), favored by the stated mutual disposition of the chromophoric units. However, this excitonic coupling provides forbidden transitions from the excited state (H aggregates are usually non-emissive). In contrast, these bis-BODIPYs displayed strong fluorescence bands, slightly red-shifted with respect to other dyads and with a marked shoulder at 580 nm (Figure 7). Moreover, in an apolar solvent like cyclohexane, the fluorescence turned out to be highly efficient (around 80%, Table 2) with surprising long lifetimes (up to 19 ns in both dyads; Figure 8, Figure S2). In this regard, the excitation and fluorescence spectra, as well as the fluorescence quantum yield and lifetime, became almost independent on the emission or/and the excitation wavelengths. All these photophysical properties could be consistent with excimer formation (long lifetimes are a fingerprint of excimer emission) upon excitation of these BODIPY dyads. Therefore, the mutual parallel and cofacial arrangement of the BODIPY units inside these dyads could enable a π-π stacking in the ground state, which led to the recorded split of the absorption bands at higher energies and, upon excitation, to subsequent geometrical rearrangement leading to the emission from intramolecular excimer species.

**Table 2:** Photophysical properties of the ether-bridged bis-BODIPYs in different solvents.

	**λ_**ab**_ (nm)**	**ε_**max**_**·**10^**−4**^ (M^**−1**^ cm^**−1**^)**	**λ_**fl**_ (nm)**	**φ**	**τ (ns)**
**4k**					
ACN	475.0/490.5	14.6/13.4	520.5	0.01	0.02 (81%)−2.26 (19%)
DMF	478.0/493.0	14.7/13.2	522.5	0.01	0.03 (81%)−2.09 (11%)−3.00 (8%)
EtOH	476.5/497.5	15.1/12.8	521.0	0.03	0.13 (63%)−2.00 (37%)
c-hex	477.5/492.5	16.0/12.6	521.5	0.82	19.29
**5k**					
ACN	495.5/514.0	13.6/12.7	545.0	0.06	3.36 (26%)−5.52 (74%)
DMF	497.0/515.5	13.0/13.1	546.0	0.15	7.00
EtOH	496.0/514.5	14.0/13.3	545.5	0.29	10.11
c-hex	496.5/515.0	14.1/14.9	545.0	0.84	19.34

*c-hex, cyclohexane; EtOH, ethanol; DMF, dimethylformamide; ACN, acetonitrile. Absorption (λ_ab_) and fluorescence (λ_fl_) wavelength, molar absorption at the maximum (ε_max_), fluorescence quantum yield (φ), and lifetime (τ)*.

Regarding the fluorescence efficiency and lifetime, the ether-bridged dyads **4k** and **5k** exhibited similar dependence on the solvent polarity to that previously described for the other BODIPY dyads herein synthesized (Table 2 vs. Table 1). Therefore, in spite of the excimer formation, the emission of the ether-dyads was still mediated by an ICT process. However, and with respect to other moieties acting as BODIPY linkers, the lower electron release of the ether bridge should significantly reduce its ability to promote ICT itself. Hence, we hypothesized that the intramolecular charge transfer had to take place mainly through other mechanisms, such as an SBCT process and the so-called intervalence charge transfer (IVCT), invoked by some authors to account for the emission behavior recorded from closely packed intramolecular BODIPY dimers (Benniston et al., 2010), the cofacial arrangement of which led also to effective excimer formation. The significant influence of the bridging moiety on the emission behavior of BODIPY dyads, as well as its solvent-sensitivity, highlighted the complexity of the molecular dynamics upon excitation, which involved an effective excitonic coupling and the ensuing formation of an emissive excimer coexisting with an effective ICT process in polar media.

### Laser Properties of Bridged bis-BODIPYs

According to the absorption properties of the new bis-BODIPYs, their lasing properties were studied under pumping at 355 nm (dyads derived from scaffold **2**) and 532 nm (dyads derived from scaffold **3**). All the dyes studied in this work exhibited broad-line-width laser emission, with a pump threshold energy of ~0.8 mJ, divergence of 5 mrad, and a pulse duration of 8 ns full-width at half maximum (FWHM), placed in a simple plane-plane non-tunable resonator cavity. The laser emission peaked at ca. 541 and 563 nm for the dyads derived from scaffolds **2** and **3**, respectively. Following the photophysical analysis, the actual effect of the solvent on the dye laser action was analyzed for solutions of polar and apolar solvents. Although the photophysical studies showed that the new derivatives exhibited their highest fluorescence capacity when dissolved in apolar solvents such as cyclohexane (Figures 5, 6), the low solubility of BODIPY dyes in this solvent prevented the concentrated solutions required for laser experiments from being attained. To analyze the dependence of the laser action on the medium polarity, we then carried out the experiments in solvents of increasing polarity enabling at the same time good solubility of the new dyes, such as ethanol, DMF, and acetonitrile.

**Figure 9:**
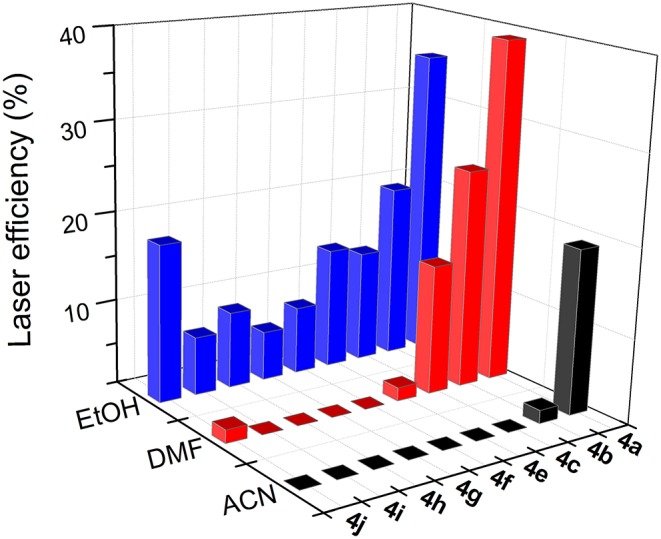
Dependence of lasing efficiency on solvent polarity in the bis-BODIPYs derived from building block **2**.

**Figure 10:**
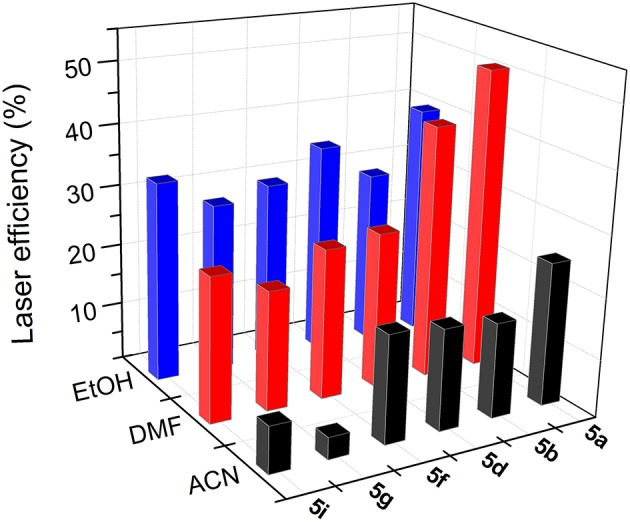
Dependence of lasing efficiency on solvent polarity in the bis-BODIPYs derived from building block **3**.

To optimize the laser action of the new dyes in the different solvents, we first analyzed the dependence of their lasing properties on dye concentration in an ethanolic solution by varying the optical densities over an order of magnitude while keeping all other experimental parameters constant (see experimental section Laser Properties). It should be noted that the optimal concentration for these bis-BODIPYs was about three-fold lower than those required to induce effective laser action in similar commercial BODIPYs (PM546 and PM567) as well as in their mono-BODIPY precursors (scaffolds **2** and **3**) (Del Río et al., 2017). The lasing behavior of the new compounds (Figures 9, 10) agreed with their photophysical properties, with the fluorescence quantum yield and the lasing efficiency showing a similar dependence on the solvent polarity and the substitution pattern of the BODIPY core as well as the spacer moiety:

- The higher the polarity of the solvent, the lower the lasing efficiency became; for the same skeleton and spacer, the lowest laser efficiency was always registered in acetonitrile.
- The higher the degree of substitution in the BODIPY core, the higher the emission efficiency became. Therefore, for the same solvent and spacer, the laser efficiencies recorded for dyads derived from scaffold **2** were consistently lower than those displayed by dyads built on the fully alkylated BODIPY **3**.
- The ability of DMF to induce specific interactions with the dyads linked by urea and thiourea bridges led to a significant increase in the laser efficiency with respect to those recorded in a less polar solvent such as ethanol. In fact, the highest lasing efficiencies among all the targets synthesized herein were recorded from the dyads **5a** (52%) and **5b** (42%) built on scaffold **3** with urea and thiourea as linkers.
- Shortening the spacer and/or reducing its electron donor ability impaired the laser action significantly. Thus, dyads built on scaffold **2** and linked by moieties **d-j** only showed effective laser emission in ethanol, not becoming laser emitters in more polar solvents such as acetonitrile. This is not the case of the derivatives from skeleton **3**, which maintained laser emission, even in acetonitrile, although, depending on the **d-j** spacer, the efficiency could be just 4%.

An important parameter for any practical applications of these bis-BODIPYs was their lasing photostability under hard radiation conditions and long operation times. A reasonable evaluation of the photostability of these dyads can be obtained by irradiating a small amount of ethanolic solution with exactly the same pumping energy and geometry as used in the laser experiments and monitoring the evaluation of the laser-induced fluorescence intensity with respect to the number of pump pulses under transversal excitation at 355 and 532 nm, with 5 mJ/pulse and a 10-Hz repetition rate after 100,000 pump pulses (see experimental section Laser Properties). To properly compare the behavior of the new dyads, the lasing photostabilities of commercial PM546 and PM567 as scaffolds of the new bis-BODIPY derivatives were also analyzed under otherwise identical experimental conditions. Both commercial dyes displayed good photostability under drastic pumping conditions, with the laser emission losing 50 and 25% of its efficiency, respectively, after 100,000 pump pulses at a 10-Hz repetition rate. Nevertheless, the laser action of the bis-BODIPYs derived from such commercial dyes was superior, with high lasing efficiencies and no signs of degradation under the same pumping conditions.

The main result that draws our attention is the capacity of the bis-BODIPYs to lase with high efficiency and photostability, although their fluorescence quantum yields in ethanol never exceeded 30% (see Figures 5, 6). Rational design linking the BODIPY units in these bis-BODIPYS is key in accounting for their lasing behavior. On the one hand, it enables an absorption increase at the pumping wavelength, which leads to a significant decrease in the dye concentration in the active medium, drastically reducing deleterious effects such as reabsorption/reemission and aggregation processes, which becomes particularly important when highly concentrated solutions are required to induce efficient laser emission. On the other hand, the ongoing ICT process leads to a short fluorescence lifetime, which allows radiative rate constants similar to that observed for other BODIPY dyes to be reached. These facts account for the origin and unique features of the laser behavior of these bis-BODIPYs.

## Conclusions

The length and stereoelectronic properties of the spacer linking the chromophoric building blocks in the symmetric bridged bis-BODIPYs designed herein played a key role in their photophysical and laser signatures. The computationally assisted spectroscopic characterization carried out herein unambiguously revealed the complex and intriguing excited state dynamics induced in these new multichromophoric systems. In fact, the photonic behavior (fluorescence and lasing efficiency) of all the tested BODIPY dyads became highly sensitive to the solvent polarity owing to the activation of ICT processes, whose effectiveness and mechanism strongly depended on the moiety acting as a spacer. We highlight that even with non-polar solvents, the connector moiety modulated the emission efficiency as follows: the higher the electron donor ability of the linker, and/or the smaller the distance between the BODIPY cores interposed by the spacer, the lower the emission efficiency and photostability became. Furthermore, up to three potential charge transfer mechanisms could be promoted by the different moieties acting as linkers in these BODIPY dyads: direct ICT between the spacer and the BODIPY core and/or a photoinduced symmetry breaking charge transfer (SBCT) and/or an intervalence charge transfer (IVCT) in closely-packed dyads, the extension and balance of which defined the final properties of these symmetric BODIPY dyads. Besides, some spacers were also able to promote intramolecular excitonic coupling, leading to an excimer-like emission. Collectively, the present work represents a breakthrough in the complex relationship between the molecular structure and the photophysical signatures of multichromophoric systems, providing key guidelines to rationalize the design of tailored photonic materials for advanced applications.

## Experimental Section

### General Experimental Methods

All solvents and reagents were obtained commercially and used as received unless stated otherwise. Residual water was removed from starting compounds by repeated coevaporation with toluene. Reactions were executed at ambient temperatures unless stated otherwise. All moisture-sensitive reactions were performed in dry flasks fitted with glass stoppers or rubber septa under a positive pressure of argon. Anhydrous MgSO_4_ or Na_2_SO_4_ was used to dry organic solutions during workup, and evaporation of the solvents was performed under reduced pressure using a rotary evaporator. Flash column chromatography was performed using 230–400 mesh silica gel. Thin-layer chromatography was conducted on a Kieselgel 60 F254. Spots were observed under UV irradiation (254 nm). ^1^H, ^13^C, ^19^F, and ^31^P NMR spectra were recorded in CDCl_3_ or CD_3_OD at 300, 400, or 500 MHz, 75, 101, or 126, 376, and 161.97 MHz, respectively. Chemical shifts are expressed in parts per million (δ scale) downfield from tetramethylsilane and are referenced to residual protium in the NMR solvent (CHCl_3_: δ 7.25 ppm; CD_3_OD: 4.870 ppm). Mass spectra were recorded by direct injection with an Accurate Mass Q-TOF LC/MS spectrometer equipped with an electrospray ion source in positive mode.

Compounds **2**, **3, 6a, 7a**, and **7b** were prepared following the methods described in Del Río et al. (2017). A full detailed description of the synthesis protocols for the preparation of the required monomeric BODIPY units **6b**, **8a**, **8b**, **9a**, **9b**, **10b**, **11a**, **11b**, and **12**, along with the characterization and copies of NMR spectra of all products, is included in the Supplementary Material.

### General Procedures for the Preparation of Symmetrical bis-BODIPYs

#### Urea-Bridged bis-BODIPYs

The appropriate azidomethyl-BODIPY **7** (0.2 mmol) was added at room temperature to a mixture of 1M triethylammonium hydrogen carbonate buffer (0.44 mmol) and 1,4-dioxane (1.0 mL). Next, triphenylphosphine (0.026 mmol) was added, and the resulting mixture was stirred at room temperature. The reaction progress was monitored by TLC. After the disappearance of the starting material, the solvent was evaporated *in vacuo* to dryness. The residue was then purified by flash chromatography on silica gel (hexane-ethyl acetate 8:2).

#### Thiourea-Bridged bis-BODIPYs

The appropriate BODIPY-isothiocyanate **10** (0.1 mmol) was dissolved in 1,4-dioxane (2 mL) and treated with the corresponding azidomethyl-BODIPY **7** (0.1 mmol), water (0.5 mL), and triphenylphosphine (0.15 mmol). The resulting solution was stirred under argon at room temperature for 24 h and then concentrated. The ensuing residue was then purified by chromatography on silica gel (hexane-ethyl acetate 7:3).

#### Phosphonate-Bridged bis-BODIPYs

The appropriate BODIPY-isothiocyanate **10** (0.1 mmol) was dissolved in 1,4-dioxane (2 mL) and treated with the corresponding azidomethyl-BODIPY **7** (0.1 mmol), water (0.5 mL), and triphenylphosphine (0.15 mmol). The resulting solution was stirred under argon at room temperature for 24 h and then concentrated. The ensuing residue was then purified by chromatography on silica gel (hexane-ethyl acetate 7:3).

#### Phosphate-Bridged bis-BODIPYs

Iodine (0.15 mmol) and water (30 μL) were added to a cooled solution (0°C) of H-phosphonate (0.05 mmol) in pyridine (1 mL). The reaction mixture was allowed to warm to room temperature and stirred for 1 h. The crude was then poured into a saturated solution of sodium sulfite (10 mL) and extracted with AcOEt (3 × 20 mL). The combined organic solutions were washed with HCl 5%, dried, and concentrated. The resulting crude mixture was purified by chromatography on silica gel (CH_2_Cl_2_-MeOH; 9:1).

#### Amine-Bridged bis-BODIPYs

A mixture of the corresponding aldehyde **6** (0.06 mmol) and the appropriate amine **9** (1 equiv.) was refluxed overnight in methanol (3 mL) under argon. The mixture was then concentrated *in vacuo*. The residue was dissolved in acetic acid (3 mL), and sodium cyanoborohydride (3 equiv.) was added. The reaction mixture was stirred at room temperature for 24 h and then concentrated, and the residue was purified by chromatography on silica gel (hexane-ethyl acetate 85:15).

#### Acetamide-Bridged bis-BODIPYs

Ac_2_O (10 equiv.) was added to a stirred solution of aminomethyl dimer **4f** or **5f** (0.012 mmol) in pyridine (2 mL). The reaction mixture was stirred at room temperature overnight and then concentrated. The resulting crude mixture was purified by chromatography on silica gel (hexane-ethyl acetate; 8:2).

#### Ammonium-Bridged bis-BODIPYs

The corresponding ***amine-bridged bis-BODIPYs*** dissolved in diethyl ether under argon was added to a solution of tetrafluoroboric acid (0.034 mmol) in diethyl ether (3 mL). The mixture was stirred for 20 min. The precipitate was filtered and extensively washed with diethyl ether.

#### Disulfide-Bridged bis-BODIPYs

The appropriate thioacetate, **11a**, **11b**, or **12SAc** (see Supplementary Material) (0.11 mmol), was dissolved in ^i^PrOH (3 mL), and K_2_CO_3_ (2 equiv.) was added. After stirring for 24 h, the mixture was poured into 10 mL of water and extracted with CH_2_Cl_2_ (3 × 20 mL); the organic layer was dried over sodium sulfate and concentrated. The crude reaction mixture was purified by flash chromatography (hexane-ethyl acetate 8:2).

#### Ether-Bridged bis-BODIPYs

The appropriate formyl-BODIPY **6** (0.07 mmol), dissolved in anhydrous CH_2_Cl_2_ (3 mL) under an argon atmosphere, was treated with triethylsilane (0.035 mmol) and indium trichloride (0.07 mmol). After stirring for 24 h, the mixture was washed with sodium bicarbonate and extracted with CH_2_Cl_2_ (3 × 20 mL), and the organic layer was dried over sodium sulfate and concentrated. The crude was purified by flash chromatography (hexane-ethyl acetate 98:2).

### Characterization Data of bis-BODIPYs

**Urea-dimer (4a)**: Obtained from azide **7a** according to procedure A. (Yield = 36 mg, 95 %;) m. p. 92–93°C ^1^H NMR (300 MHz, CDCl_3_) δ 7.57–7.29 3 (m, 6H), 7.21–7.07 (m, 2H), 5.94 (s, 4H), 4.59 (t, *J* = 6.2 Hz, 2H), 4.14 (d, *J* = 6.1 Hz, 4H), 2.51 (s, 12H), 1.33 (s, 12H). ^13^C NMR (75 MHz, CDCl_3_) δ 158.1, 156.1, 143.4, 140.8, 137.6, 133.2, 131.2, 130.0, 128.8, 128.8, 128.5, 128.3, 121.8, 42.5, 14.9, 14.3. HRMS (ESI-TOF): calcd for C_41_H_42_B_2_F_4_ONaN_6_: [M + Na]^+^ 755.3440, found 755.3382.

**Urea-dimer (5a)**: Obtained from azide **7b** according to procedure A. (Yield = 38 mg, 90 %); m. p. 96–98°C; ^1^H NMR (400 MHz, CDCl_3_) δ 7.49–7.30 (m, 4H), 7.28–7.24 (m, 2H), 7.14–7.00 (m, 2H), 4.67–4.48 (m, 2H), 4.16–3.96 (m, 4H), 2.40 (s, 12H), 2.17 (q, *J* = 7.5 Hz, 8H), 1.16 (s, 12H), 0.86 (t, *J* = 7.6 Hz, 12H); ^13^C NMR (101 MHz, CDCl_3_) δ 157.7, 153.9, 138.8, 138.3, 137.5, 133.6, 133.0, 130.1, 129.3, 128.4, 127.8, 42.2, 17.0, 14.5, 11.1. HRMS (ESI-TOF): calcd for C_49_H_58_B_2_F_4_N_6_NaO: [M + Na]^+^ 867.47022, found 867.46969.

**Thiourea-dimer (4b)**: Obtained from azide **7a** and isothiocyanate **10a** according to procedure B. (Yield = 24 mg, 65%); m. p. 152–153°C; ^1^H NMR (300 MHz, CDCl_3_) δ 7.44–7.39 (m, 6H), 7.19–7.12 (m, 2H), 6.02 (t, *J* = 6.3 Hz, 2H), 5.95 (s, 4H), 4.49 (d, *J* = 5.9 Hz, 4H), 2.49 (s, 12H), 1.34 (s, 12H); ^13^C NMR (75 MHz, CDCl_3_) δ 183.6, 155.9, 143.2, 140.1, 135.8, 133.1, 130.9, 129.7, 129.1, 128.4, 128.3, 121.6, 46.0, 14.6, 14.2. HRMS (ESI-TOF): calcd for C_41_H_42_B_2_F_4_SNaN_6_: [M + Na]^+^ 771.3212, found 771.3177.

**Thiourea-dimer (5b)**: Obtained from azide **7b** and isothiocyanate **10b** according to procedure B. (Yield = 25 mg, 58%); ^1^H NMR (400 MHz, CDCl_3_) δ 7.48–7.31 (m, 6H), 7.17 (dt, *J* = 7.3, 1.0 Hz, 2H), 5.80 (t, *J* = 6.3 Hz, 2H), 4.45 (d, *J* = 6.2 Hz, 4H), 2.45 (s, 6H), 2.25 (q, *J* = 7.5 Hz, 4H), 1.26 (s, 6H), 0.94 (t, *J* = 7.5 Hz, 6H); ^13^C NMR (126 MHz, CDCl_3_) δ 179.1, 154.3, 154.1, 138.9, 138.7, 138.4, 137.6, 133.7, 133.4, 133.2, 130.3, 130.2, 129.5, 128.9, 128.8, 128.6, 128.1, 128.0, 42.3, 29.5, 17.2, 14.7, 11.3. HRMS (ESI-TOF): calcd for C_49_H_58_B_2_F_4_N_6_NaS: [M + Na]^+^ 883.4464, found 883.4437.

**Thiourea-bridged bis-BODIPY (5c**). Obtained from BODIPY-isothiocyanate **10a** and 1,4-phenylenediamine (1.5 equiv). (Yield = 25 mg, 45%); m. p. 175–176°C; ^1^H NMR (500 MHz, CDCl_3_) δ 7.74–7.69 (m, 2H), 7.48–7.41 (m, 6H), 7.38 (ddd, *J* = 7.8, 7.6,1.3 Hz, 2H), 7.15 (dd, *J* = 7.6, 1.4 Hz, 2H), 6.86–6.80 (m, 2H), 6.63–6.57 (m, 2H), 6.06–5.98 (m, 2H), 5.90 (s, 4H), 4.76 (d, *J* = 6.2 Hz, 4H), 2.54 (s, 12H), 1.28 (s, 12H); ^13^C NMR (75 MHz, CDCl_3_) δ 181.8, 156.0, 146.4, 142.8, 139.4, 135.2, 133.8, 130.8, 130.0, 129.7, 128.6, 127.5, 125.8, 116.0, 46.4, 14.8, 14.2. HRMS (ESI-TOF): calcd for C_48_H_49_B_2_F_4_S_2_N_8_: [M + H]^+^ 899.3644, found 899.3680.

**H-phosphonate-dimer (4d)**: Obtained from alcohol **2** and PBr_3_ according to procedure C. (Yield = 175 mg, 48%); ^1^H NMR (500 MHz, CDCl_3_) δ 7.58–7.44 (m, 6H), 7.27–7.22 (m, 2H), 6.59 (d, *J* = 707 Hz, 1H), 5.95 (s, 4H), 4.96–4.82 (m, 4H), 2.54 (s, 12H), 1.30 (s, 6H), 1.29 (s, 6H); ^13^C NMR (125 MHz, CDCl_3_) δ 156.1, 142.9, 138.4, 133.7, 133.1, 130.9, 129.8, 129.6, 129.4, 128.5, 121.6, 121.5, 64.5, 64.4, 14.6, 13.8; ^31^P NMR (161.97 MHz, CDCl_3_) δ 9.18.

**H-phosphonate-dimer (5d)**: Obtained from alcohol **3** and PBr_3_ according to procedure C. (Yield = 189 mg, 52%); ^1^H NMR (500 MHz, CDCl_3_) 7.54–7.41 (m, 6H), 7.26–7.20 (m, 2H), 6.61 (d, *J* = 707.0 Hz, 1H), 5.00–4.85 (m, 4H), 2.52 (s, 12H), 2.26 (q, *J* = 7.5 Hz, 8H), 1.20 (s, 6H), 1.19 (s, 6H), 0.95 (t, *J* = 7.5 Hz, 12H). HRMS (ESI-TOF): calcd for C_48_H_61_B_2_F_4_N_5_O_3_P: [M+NH_4_]^+^ 884.46442, found: 884.46722. calcd for C_48_H_57_B_2_F_4_N_4_NaO_3_P: [M+Na]^+^ 889.41981, found: 889.41976.

**Phosphate-dimer (4e**). Obtained from H-phosphonate bis-BODIPY **4d** according to procedure D. (Yield = 13.5 mg, 65%). ^1^H NMR (500 MHz, CDCl_3_) δ 7.62–7.51 (m, 3H), 7.34–7.24 (m, 3H), 7.13–7.05 (m, 2H), 5.81 (m, 4H), 4.69–4.49 (m, 4H), 2.47 (s, 12H), 1.18 (s, 12H); ^31^P NMR (161.97 MHz, CDCl_3_) δ −2.16.

**Amine-dimer (4f)**: Obtained from aldehyde **6a** and amine **9a** according to procedure E. (Yield = 97.3 mg, 45%); ^1^H NMR (500 MHz, CDCl_3_) δ 7.47–7.31 (m, 6H), 7.15–7.13 (m, 2H), 5.94 (s, 4H), 3.62 (s, 4H), 2.54 (s, 12H), 1.27 (s, 12H); ^13^C NMR (125 MHz, CDCl_3_) δ 155.6, 142.9, 140.6, 137.8, 133.7, 131.2, 129.5, 128.3, 128.2, 127.9, 121.4, 50.8, 14.7, 13.9. HRMS (ESI-TOF): calcd for C_40_H_42_B_2_F_4_N_5_: [M+H]^+^ 690.35625, found: 690.35814.

**Amine-dimer (5f)**: Obtained from aldehyde **6b** and amine **9b** according to procedure E. (Yield = 15.4 mg, 40%); ^1^H NMR (500 MHz, CDCl_3_) 7.45–7.30 (m, 6H), 7.14–7.12 (m, 2H), 3.62 (s, 4H), 2.51 (s, 12H), 2.27 (q, *J* = 7.5 Hz, 8H), 1.18 (s, 12H), 0.94 (t, *J* = 7.5 Hz, 12H); ^13^C NMR (125 MHz, CDCl_3_) 153.8, 138.9, 138.1, 138.0, 134.5, 132.9, 130.4, 129.3, 128.5, 128.0, 127.7, 50.6, 17.2, 14.7, 12.6, 11.2). HRMS (ESI-TOF): calcd for C_48_H_58_B_2_F_4_N_5_: [M+H]^+^ 802.48243, found: 802.48314.

**Acetamide-dimer (4g)**: Obtained from amine-bridged bis-BODIPY **4f** according to procedure F. (Yield = 4.5 mg, 85%); ^1^H NMR (500 MHz, CDCl_3_) δ 7.50 (m, 1H), 7.44–7.38 (m, 2H), 7.34 (m, 1H), 7.20 (m, 1H), 7.14–7.11 (m, 2H), 5.95 (s, 2H), 5.93 (s, 2H), 4.36 (s, 2H), 4.31 (s, 2H), 2.54 (s, 6H), 2.51 (s, 6H), 1.96 (s, 3H), 1.28 (s, 6H), 1.21 (s, 6H); ^13^C NMR (125 MHz, CDCl_3_) δ 171.6, 156.4, 155.8, 143.1, 142.3, 139.8, 139.0, 134.2, 133.9, 133.1, 132.8, 130.7, 130.6, 130.5, 129.9, 129.2, 128.8, 128.7, 128.1, 126.5, 126.2, 121.7, 121.4, 51.5, 49.6, 21.0, 14.8, 14.7, 14.0, 13.9. HRMS (ESI-TOF): calcd for C_40_H_43_B_2_F_4_N_5_NaO: [M+Na]^+^ 754.34957, found: 754.35211.

**Acetamide-dimer (5g)**: Obtained from amine-bridged bis-BODIPY **5f** according to procedure F. (Yield = 9.1 mg, 90%); ^1^H NMR (500 MHz, CDCl_3_) δ 7.47 (td, *J* = 7.6, 1.4 Hz, 1H), 7.41 (td, *J* = 7.6, 1.3 Hz, 1H), 7.37 (dd, *J* = 7.5, 1.6 Hz, 1H), 7.34 (td, *J* = 7.5, 1.5 Hz, 1H), 7.22 (dd, *J* = 7.4, 1.4 Hz, 1H), 7.19 (dd, *J* = 7.8, 1.2 Hz, 1H), 7.16 (dd, *J* = 7.3, 1.6 Hz, 1H), 7.10 (dd, *J* = 7.7, 1.4 Hz, 1H), 4.43 (s, 2H), 4.36 (s, 2H), 2.53 (s, 6H), 2.52 (s, 6H), 2.30–2.24 (m, 8H), 1.86 (s, 3H), 1.23 (s, 6H), 1.16 (s, 6H), 0.95 (t, *J* = 7.6 Hz, 12H). HRMS (ESI-TOF): calcd for C_50_H_59_B_2_F_4_N_5_NaO_2_: [M+Na]+ 882.46991, found: 882.47366.

**Ammonium-dimer (4h**). Obtained from amine-bridged bis-BODIPY **4f** according to procedure G. (Yield = 10 mg, 80%); ^1^H NMR (300 MHz, CD_3_OD) δ 7.70 (m, 6H), 7.52 (d, *J* = 7.5 Hz, 2H), 6.12 (s, 4H), 4.21 (bs, 4H), 3.32 (s, 12H), 2.99 (d, *J* = 8.7 Hz, 2H), 2.53 (s, 12H). HRMS (ESI-TOF) (positive mode): calcd for C_40_H_42_B_2_F_4_N_5_: [M+H]^+^ 690.35625, found: 690.35822; HRMS (ESI-TOF) (negative mode): [BF_4_]^−^ 87.00326, found: 86.00634.

**Disulfide-dimer** (**4i)**: Obtained from thiolacetate **11a** according to procedure H. (Yield = 18 mg, 40%); ^1^H NMR (500 MHz, CDCl_3_) δ 7.40–7.35 (m, 6H), 7.19–7.17 (m, 2H), 5.97 (s, 4H), 3.77 (s, 4H), 2.54 (s, 12H), 1.35 (s, 12H); ^13^C NMR (125 MHz, CDCl_3_) δ 155.9, 143.3, 139.5, 134.9, 134.3, 131.3, 131.0, 129.7, 128.7, 128.5, 121.5, 41.1, 14.8, 14.5. HRMS (ESI-TOF): calcd for C_40_H_41_B_2_F_4_N_4_S_2_: [M+H]^+^ 739.29027, found: 739.28922. calcd for C_40_H_44_B_2_F_4_N_5_ S_2_: [M+NH_4_]^+^ 756.31682, found: 756.31544; calcd for C_40_H_40_B_2_F_4_N_4_NaS_2_: [M+Na]^+^ 761.27222, found: 761.27138.

**Disulfide-dimer** (**5i)**: Obtained from thiolacetate **11a** according to procedure H. (Yield = 21 mg, 45%); ^1^H NMR (500 MHz, CDCl_3_) δ 7.40–7.33 (m, 6H), 7.19–7.17 (m, 2H), 3.76 (s, 4H), 2.51 (s, 12H), 2.29 (q, *J* = 7.5 Hz, 4H), 1.24 (s, 12H), 0.96 (t, *J* = 7.5 Hz, 6H).^13^C NMR (125 MHz, CDCl_3_) δ 154.2, 138.6, 137.9, 135.2, 135.0, 133.1, 130.8, 130.6, 129.4, 129.0, 128.3, 41.2, 17.2, 14.7, 12.7, 11.8. HRMS (ESI-TOF): calcd for C_48_H_57_B_2_F_4_N_4_S_2_: [M+H]^+^ 851.41569, found: 851.41495; calcd for C_48_H_60_B_2_F_4_N_5_S_2_: [M+NH_4_]^+^ 868.44224, found: 868.44135; calcd for C_48_H_56_B_2_F_4_N_4_NaS_2_: [M+Na]^+^ 873.39764, found: 873.39923.

**Elongated disulfide-dimer (5j)**: Obtained from thiolacetate **12Sac** (see Supplementary Material) according to procedure H. (Yield = 14.5 mg, 42%); ^1^H NMR (500 MHz, CDCl_3_) 7.41(dt, *J* = 7.5, 1.5 Hz, 2H), 7.35–7.31 (m, 4H), 7.18–7.16 (m, 2H), 5.95 (s, 4H), 2.84 (dd, J = 8.9, 6.5 Hz, 4H), 2.60 (dd, *J* = 8.7, 6.7 HZ, 4H), 2.52 (s, 12H), 1.34 (s, 12H).^13^C NMR (125 MHz, CDCl_3_) δ 155.7, 142.8, 140.6, 137.4, 134.4, 131.3, 130.3, 129.5, 128.5, 127.5, 121.4, 37.2, 32.5, 14.7, 14.3. HRMS (ESI-TOF): calcd for C_42_H_45_B_2_F_4_N_4_S_2_: [M+H]^+^ 767.32163, found 767.32171; calcd for C_42_H_48_B_2_F_4_N_5_S_2_: [M+NH_4_]^+^ 784.34818, found 784.34983.

**Ether-dimer** (**4k)**: Obtained from aldehyde **6a** according to procedure I. (Yield = 8 mg, 56%); ^1^H NMR (300 MHz, CDCl_3_) δ 8.07–7.96 (m, 2H), 7.53–7.41 (m, 2H), 7.30 (t, *J* = 7.5 Hz, 2H), 7.07 (dd, *J* = 7.5, 1.3 Hz, 2H), 5.80 (s, 4H), 3.78 (s, 4H, 2.46 (s, 12H), 1.21 (s, 12H). HRMS (ESI-TOF): calcd for C_40_H_41_B_2_F_4_N_4_O: [M+H]^+^ 691.3403, found: 691.3389.

**Ether-dimer** (**5k)**: Obtained from aldehyde **6b** according to procedure I. (Yield = 5 mg, 48%); ^1^H NMR (300 MHz, CDCl_3_) δ 7.99 (d, *J* = 7.7 Hz, 2H), 7.54–7.39 (m, 2H), 7.33–7.29 (m, 2H), 7.11–7.04 (m, 2H), 3.63 (s, 4H), 2.44 (s, 12H), 2.14 (q, J = 7.5 Hz, 8H), 1.08 (s, 12H), 0.84 (t, *J* = 7.5 Hz, 12H). HRMS (ESI-TOF): calcd for C_48_H_57_B_2_F_4_N_4_O: [M+H]^+^ 803.4655, found: 803.4639.

### Spectroscopic Properties

Spectroscopic properties were registered in diluted solutions (around 2 × 10^−6^ M) prepared by adding the corresponding solvent (spectroscopic grade) to the residue from an adequate amount of a concentrated stock solution in acetone after vacuum evaporation of this solvent. UV-Vis absorption spectra were recorded on a Varian model CARY 4E spectrophotometer, whereas the fluorescence and excitation spectra were registered in an Edinburgh Instruments spectrofluorimeter (model FLSP 920), as were the decay curves. Fluorescence quantum yields (φ) were obtained using commercial BODIPYs in ethanol (PM546 φ_r_ = 0.85 and PM567 φ_r_ = 0.84) as a reference. The values were corrected by the refractive index of the solvent. Radiative decay curves were registered with the time-correlated single-photon counting technique using a multichannel plate detector with picosecond time resolution. Fluorescence emission was monitored at the maximum emission wavelength after excitation by means of a wavelength-tunable Fianium Supercontinuum laser. The fluorescence lifetime (τ) was obtained after the deconvolution of the instrumental response signal from the recorded decay curves by means of an iterative method. The decay curve was essentially the same regardless of the excited visible absorption band. The goodness of the exponential fit was controlled by statistical parameters (chi-square and the analysis of the residuals).

### Quantum Mechanical Calculations

Ground state geometries were optimized at the Density Functional Theory (DFT) level using the range-separated hybrid wB97XD method and the triple-valence basis set with one polarization functions (6–311 g^*^). To check that the optimized geometries correspond to a true energy minimum, the corresponding frequency analysis was conducted (no negative value). The solvent effect (ethanol) was considered in the theoretical simulations by means of the Polarizable Continuum Model (PCM). All the calculations were performed using Gaussian 16 software as implemented in the computational cluster “arina” of the UPV/EHU.

### Laser Properties

Liquid solutions of dyes were contained in 1-cm optical-path rectangular quartz cells carefully sealed to avoid solvent evaporation during the experiments. The liquid solutions were transversely pumped with 5-mJ, 8-ns FWHM pulses from the second harmonic (532 nm) and the third harmonic (355 nm) of a Q-switched Nd:YAG laser (Lotis TII 2134) at a repetition rate of 1 Hz. The exciting pulses were line-focused onto the cell using a combination of positive and negative cylindrical lenses (f = 15 cm and f = −15 cm, respectively) perpendicularly arranged. The plane-parallel oscillation cavity (2 cm length) consisted of a 90%-reflectivity aluminum mirror acting as the back reflector and the lateral face of the cell acting as output coupler (4% reflectivity). The pump and output energies were detected by a GenTec powermeter. The photostability of the dyes in ethanol was evaluated by using a pumping energy and geometry exactly equal to that of the laser experiments. We used spectroscopic quartz cuvettes with a 0.1 cm optical path to allow for the minimum solution volume (40 μL) to be excited. The lateral faces were grounded, whereupon no laser oscillation was obtained. Information about photostability was obtained by monitoring the decrease in laser-induced fluorescence (LIF) intensity after 100,000 pump pulses at a 10 Hz repetition rate to speed up the running of the experiment. The fluorescence emission and laser spectra were monitored perpendicular to the exciting beam, collected by an optical fiber, imaged onto a spectrometer (Acton Research corporation), and detected with a charge-coupled device (CCD) (SpectruMM:GS128B). The fluorescence emission was recorded by feeding the signal to the boxcar (Stanford Research, model 250) to be integrated before being digitized and processed by a computer. The estimated error in the energy and photostability measurements was 10%.

## Data Availability Statement

All datasets generated for this study are included in the article/Supplementary Material.

## Author Contributions

AO-S conducted the photophysical measurements, whereas RS-L carried out the theoretical calculations. JB supervised the joint experimental and theoretical study and drafted the manuscript. IG-M performed the laser measurements and provided a critical review of the whole manuscript. JL participated in the design of the BODIPY dimers and carried out some of the synthetic work. CU participated in the design of the BODIPY dimers and carried out most of the synthetic work. AG designed the BODIPY dimers, participated in the synthetic work, and contributed to the final writing of the manuscript.

### Conflict of Interest

The authors declare that the research was conducted in the absence of any commercial or financial relationships that could be construed as a potential conflict of interest.
