# Supplementary material for: Tuning the Photonic Behavior of Symmetrical bis-BODIPY Architectures: The Key Role of the Spacer Moiety
Source: Front Chem. 2019 Dec 3;7:801. doi: 10.3389/fchem.2019.00801 (PMC6902057; doi:10.3389/fchem.2019.00801)
Supplement: Supplementary file 1 [file Data_Sheet_1.PDF]

Supplementary Material

Tuning the photonic behavior of symmetrical bis-BODIPY architectures: the key role of the spacer moiety

Ainhoa Oliden[1], Rebeca Sola-Llano[1], Jorge Bañuelos[1*], Inmaculada García-Moreno[2], Clara Uriel[3], J. Cristobal Lopez[3] and Ana M. Gomez[3*]

[1]Molecular Spectroscopy Laboratory, Science and Technology Faculty, Physical Chemistry Department, Basque Country University (UPV/EHU), Bilbao, Spain

[2]Laser Materials Laboratory, "Rocasolano" Physical Chemistry Institute, Department of Low-Dimension Systems, Surfaces and Condensed Matter, CSIC, Madrid, Spain

[3]Instituto de Quimica Organica General (*IQOG-CSIC*), Bioorganic Chemistry Department, Juan de la Cierva 3, 28006, Madrid, Spain.

Table of Contents

1. General Information 2

2. Experimental procedures for the preparation of the monomeric BODIPY units.........................3-7

3. Table S1 8-9

4. Table S2 10-11

5. Figure S1: Absorption and fluorescence spectra of dimer **4k**…………….....……….…………….12

6. Figure S2: Fluorescence decay curves of dimer **4k**………………………………………….......12

7. Figures S3-S21: NMR spectra (^{1}H, ^{13}C, HSQC) of monomeric units **6-12**…………………..13-23

8. Figures S22-S34: NMR spectra (^{1}H, ^{13}C, HSQC) of dimers **4a-4k**…………………………....24-35

9. Figures S35-S60: NMR spectra (^{1}H, ^{13}C, HSQC) of dimers **5a-4k**…………………………....36-46

10. Figure S61: Absorption and fluorescence spectra of dimers **4a-4k**…………………………......47-48

11. Figure S62: Absorption and fluorescence spectra of dimers **5a-5k**……………………...…....49-50

1. General Information

Unless otherwise stated, all reactions were carried out under normal atmosphere in dried glassware. Commercially available reactants were used without further purification. All moisture-sensitive reactions were performed in dry flasks fitted with glass stoppers or rubber septa under a positive pressure of argon. Air- and moisture-sensitive liquids and solutions were transferred by syringe or stainless steel cannula. Anhydrous magnesium sulphate or sodium sulphate were used to dry organic solutions during workup, and evaporation of the solvents was performed under reduced pressure using a rotary evaporator. Flash column chromatography was performed using 230–400 mesh silica gel. Thin-layer chromatography was conducted on Kieselgel 60 F254. Spots were observed under UV irradiation (254 nm).^{1}H; ^{13}C; ^{19}F; and ^{31}P NMR spectra were recorded in $CDCl_3$ or CD_3OD at 300, 400 or 500 MHz 75, 101 or 126 MHz; 376 MHz and 161.97 MHz, respectively. Chemical shifts are expressed in parts per million (δ scale) downfield from tetramethylsilane and are referenced to residual protium in the NMR solvent ($CHCl_3$: δ 7.25 ppm). Coupling constants (*J*) are given in Hz. All presented ^{13}C NMR spectra are proton-decoupled. Mass spectra were recorded by direct injection with a *Accurate Mass Q-TOF LC/MS* spectrometer equipped with an electrospray ion source in positive mode. Compounds **2**, **3, 6a, 7a, 7b** were prepared following the described method (del Rio et al., 2017).

2. Experimental procedures for the preparation of monomeric BODIPY units

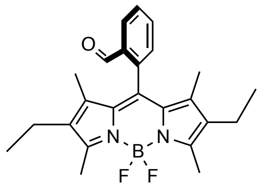

6b

8-(2-formylmethylphenyl)-2,6-diethyl-1,3,5,7-tetramethyl-4,4-difluoro-4-bora-3a,4a-diaza-*s*-indacene (6b). Dess Martin periodinane (1.03 mmol, 436 mg) was suspended in dry CH_2Cl_2 (4 mL). To this suspension was slowly added a solution of hydroxymethyl-BODIPY **3** (350 mg, 0.85 mmol) in dry CH_2Cl_2 (6 mL) at 0 °C under argon. After 10 min, the ice bath was removed and the reaction mixture was left stirring at room temperature for 3 h. The reaction mixture was washed with saturated aqueous $Na_2S_2O_3$ followed by saturated aqueous $NaHCO_3$ and brine. The combined organic solutions were dried. The solvent was evaporated, and the resulting crude mixture was purified by chromatography on silica gel (eluent: hexane–ethyl acetate 9:1) to give formyl-BODIPY **6a** (365 mg,

87%). ^{1}H NMR (500 MHz, $CDCl_3$) δ 10.0 (s, 1H), 8.12-8.09 (m, 1H), 7.77-7.71 (m, 1H), 7.67-7.61 (m, 1H), 7.41-7.38 (m, 1H), 2.55 (s, 6H), 2.29 (q, J = 7.6 Hz, 4H), 1.21 (s, 6H), 0.98 (t, J = 7.6 Hz, 6H). ^{13}C NMR (125 MHz, $CDCl_3$) δ 190.7, 154.8, 139.1, 138.0, 135.1, 134.8, 134.6, 133.5, 131.1, 129.8, 127.5, 17.1, 14.5, 12.6, 11.4. HRMS (ESI-TOF): calcd for $C_{24}H_{28}BF_2N_2O$: $[M+H]^+$ 409.22616, found: 409.22670; calcd for $C_{24}H_{27}BF_2N_2NaO$: $[M+Na]^+$ 431.20810, found: 431.20907.

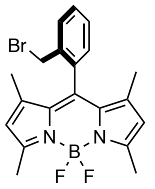

8a

8-(2-bromomethylphenyl)-1,3,5,7-tetramethyl-4,4-difluoro-4-bora-3a,4a-diaza-*s*-indacene (8a). To a stirred solution of hydroxymethyl-BODIPY **2** (100 mg, 0.28 mmol) in CH_2Cl_2 (25 mL) was added DMF (230 µL, 0.01 mmol). The solution was cooled to 0 ^{0}C and PBr_3 (40 µl, 0.42 mmol) was added. The mixture was allowed to warm to rt and then stirred for 2 h, after which the solvent was removed in vacuo. Purification by flash chromatography (gradient hexane–ethyl acetate 8:2, 7:3, 1:1) afforded compound **8a** (78 mg, 67 %). 1H NMR (500 MHz, $CDCl_3$) δ 7.66-7.64 (m, 1H), 7.50 (m, 1H), 7.42 (m, 1H), 7.21-7.19 (m, 1H), 5.99 (s, 2H), 4.42 (s, 2H), 2.57 (s, 6H), 1.38 (s, 6H). ^{13}C NMR (125 MHz, CDCl3) 156.2, 143.1, 138.5, 135.0, 134.3, 131.6, 131.2, 130.1, 129.3, 128.7, 121.6, 30.2, 14.8, 14.3. HRMS (ESI-TOF): calcd for $C_{20}H_{21}BBrF_2N_2$: [M+H]+ 417.09474, found: 417.09512.

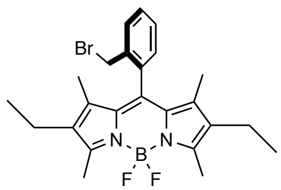

8b

8-(2-bromomethylphenyl)-2,6-diethyl-1,3,5,7-tetramethyl-4,4-difluoro-4-bora-3a,4a-diaza-*s*-indacene (8b). To a stirred solution of hydroxymethyl-BODIPY **3** (172 mg, 0.42 mmol) in CH_2Cl_2 (20 mL) was added DMF (345 µL, 0.015 mmol). The solution was cooled to 0 ^{0}C and PBr_3 (60 µl, 0.63 mmol) was added. The mixture was allowed to warm to room temperature and then stirred for 2 h, after which the solvent was removed in vacuo. Purification by flash chromatography (gradient hexane–ethyl acetate 8:2, 7:3, 1:1) afforded compound **8b** (119 mg, 60 %). 1H NMR (500 MHz, $CDCl_3$) δ 7.66 (dd, J = 7.8, 1.3 Hz, 1H), 7.50 (dt, J = 7.6, 1.5 Hz, 1H), 7.41 (td, J = 7.5, 1.4 Hz, 1H), 7.21 (dd, J = 7.6, 1.5 Hz, 1H), 4.42 (s, 2H), 2.54 (s, 6H), 2.30 (q, J = 7.6 Hz, 4H), 1.28 (s, 6H), 0.98 (t, J = 7.5 Hz, 6H). HRMS (ESI-TOF): calcd for $C_{24}H_{29}BBrF_2N_2$: $[M+H]^+$ 475.15563, found: 475.15786.

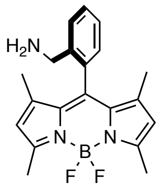

9a

8-(2-aminomethylphenyl)-1,3,5,7-tetramethyl-4,4-difluoro-4-bora-3a,4a-diaza-*s*-indacene (9a). To a stirred solution of azidomethyl-BODIPY **7a** (200 mg, 0.53 mmol) in dioxane (10 mL) was added triphenylphosphine (235 mg, 0.9 mmol) and NH_4OH (137 µL, 1.06 mmol). The reaction mixture was stirred at room temperature for 12 h and the solvent was concentrated. Purification by flash chromatography (dichloromethane-methanol, 95:5) afforded compound **9a** (153 mg, 82%). 1H NMR (500 MHz, CDCl3) δ 7.62-7.59 (m, 1H), 7.52-7.46 (m, 1H), 7.41-7.36 (m, 1H), 7.20-7.17 (m, 1H), 5.98 (s, 2H), 3.79 (bs, 2H), 2.55 (s, 6H), 1.36 (s, 6H). HRMS (ESI-TOF): calcd for $C_{20}H_{22}BF_2N_3Na$: $[M+Na]^+$ 376.17707, found: 376.17757.

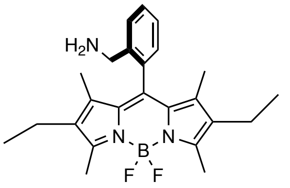

9b

8-(2-aminomethylphenyl)-2,6-diethyl-1,3,5,7-tetramethyl-4,4-difluoro-4-bora-3a,4a-diaza-*s*-indacene (9b). To a stirred solution of azidomethyl-BODIPY **7b** (160 mg, 0.37 mmol) in dioxane (8 mL) was added triphenylphosphine (164 mg, 0.63 mmol) and NH_4OH (96 µL, 0.76 mmol). The reaction mixture was stirred at room temperature for 12 h and the solvent was concentrated. Purification by flash chromatography (dichloromethane-methanol, 95:5) afforded compound **9b** (119 mg, 79%). 1H NMR (500 MHz, CDCl3) δ 7.61-7.58 (m, 1H), 7.51-7.46 (m, 1H), 7.39-7.34 (m, 1H), 7.20-7.16 (m, 1H), 3.78 (s, 2H), 2.53 (s, 6H), 2.30 (q, J = 7.5 Hz, 4H), 1.27 (s, 6H), 0.98 (t, J = 7.5 Hz, 6H). ^{13}C NMR (125 MHz, CDCl3) δ 154.0, 140.0, 138.6, 137.9, 134.1, 133.0, 130.4, 129.6, 128.5, 128.0, 127.7, 43.5, 17.1, 14.7, 12.6, 11.3. HRMS (ESI-TOF): calcd for $C_{24}H_{30}BF_2N_3Na$: $[M+Na]^+$ 432.23974, found: 432.23932.

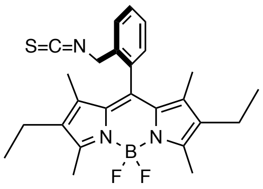

10b

8-(2-Isothiocyanatemethylphenyl)-2,6-diethyl-1,3,5,7-tetramethyl-4,4-difluoro-4-bora-3a,4a-diaza-*s*-indacene (10b). To a solution of azidomethyl-BODIPY **7b** (150 mg, 0.34 mmol) and CS_2 (63 μL, 1.02 mmol) in anhydrous THF (3 mL) was added triphenylphosphine (180 mg, 0.68 mmol, 2 equiv.). The reaction mixture was stirred at rt, under argon, for 24 h, then concentrated and the residue purified by chromatography on silica gel (eluent: hexane–ethyl acetate = 9:1) to give isothiocyanate-BODIPY **10b**. (133 mg, 87%); ^{1}H-NMR (400 MHz, $CDCl_3$) δ (ppm): 7.63 – 7.60 (m, 1H), 7.57 (td, *J* = 7.5, 1.4 Hz, 1H), 7.50 – 7.45 (m, 1H), 7.26 (s, 1H), 4.64 (s, 2H), 2.53 (s, 6H), 2.30 (q, *J* = 7.6 Hz, 4H), 1.24 (s, 6H), 0.99 (t, *J*=7.6, 6H); ^{19}F NMR (376 MHz, $CDCl_3$) δ = -146.5, -146.6, -146.7, -146.8; ^{13}C NMR (101 MHz, $CDCl_3$) δ = 154.9, 145.1, 145.0, 137.9, 136.3, 134.1, 133.5, 132.5, 132.4, 132.3, 132.3, 132.2, 130.1, 130.1, 129.4, 129.2, 128.7, 128.6, 128.2, 77.2, 46.4, 29.8, 17.2, 14.7, 12.7, 11.3. HRMS (ESI-QTOF) m/z: $[M+H]^+$ Calcd for $C_{25}H_{29}BF_2N_3S$: 452.2143; Found 452.2098.

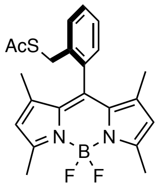

11a

8-(2-acetylthiomethylphenyl)-1,3,5,7-tetramethyl-4,4-difluoro-4-bora-3a,4a-diaza-*s*-indacene (11a). A solution of the bromomethyl-BODIPY **8a** (140 mg, 0.34 mmol) in a 1:1 mixture of ethanol-dichloromethane (4 mL) was treated with K_2CO_3 (94 mg, 0.68 mmol) and thioacetic acid (29 μL, 0.41 mmol). The mixture was allowed to react at room temperature for 12 h and then it was diluted with dichloromthane, washed with $NaHCO_3$ and the organic layer was dried over sodium sulfate, filtered and concentrated. The residue was purified by flash chromatography (hexane-ethyl acetate 9:1) to give **11a** (123 mg, 88 %).^{1}H NMR (500 MHz, $CDCl_3$) δ 7.52-7.49 (m, 1H), 7.43 (dt, J = 7.5, 1.7 Hz, 1H), 7.37 (dt, J = 7.4, 1.7 Hz, 1H), 7.20-7.17 (m, 1H), 5.99 (s, 2H), 4.06 (s, 2H), 2.56 (s, 6H), 2.29 (s, 3H), 1.38 (s, 6H). HRMS (ESI-TOF): calcd for $C_{22}H_{24}BF_2N_2OS$: [M+H]+ 413.16690, found: 413.16628; calcd for $C_{22}H_{23}BF_2N_2NaOS$: [M+Na]+ 435.14884, found: 435.14830.

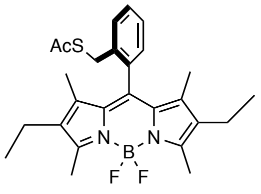

11b

8-(2-acetylthiomethylphenyl)-2,6-diethyl-1,3,5,7-tetramethyl-4,4-difluoro-4-bora-3a,4a-diaza-*s*-indacene (11b). A solution of the bromomethyl-BODIPY **8b** (250 mg, 0.53 mmol) in a 1:1 mixture of ethanol-dichloromethane (5 mL) was treated with K_2CO_3 (138 mg, 1 mmol) and thioacetic acid (45 µL, 0.64 mmol). The mixture was allowed to react at room temperature for 12 h and then it was diluted with dichloromethane, washed with $NaHCO_3$ and the organic layer was dried over sodium sulfate, filtered and concentrated. The residue was purified by flash chromatography (hexane-ethyl acetate 9:1) to give **11b** (229 mg, 92%). ^{1}H NMR (500 MHz, $CDCl_3$) δ 7.50 (dd, *J* = 7.5, 1.7 Hz, 1H), 7.42 (dt, *J* = 7.5, 1.6 Hz, 1H), 7.36 (td, *J* = 7.4, 1.6 Hz, 1H), 7.18 (dd, *J* = 7.6, 1.5 Hz, 1H), 4.06 (s, 2H), 2.54 (s, 6H), 2.31 (q, J = 7.6 Hz, 4H), 2.28 (s, 3H), 1.27 (s, 6H), 0.99 (t, *J* = 7.5 Hz, 6H). HRMS (ESI-TOF): calcd for C_{26} H_{32} B F_2 N_2 O S: $[M+H]^+$ 469.22957, found: 469.23094. calcd for $C_{26}H_{31}BF_2N_2NaOS$: $[M+Na]^+$ 491.21151, found: 491.21306.

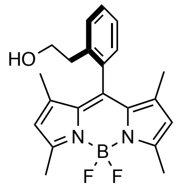

12

BODIPY 12: This compound was prepared according to the one-pot procedure described (del Rio et al., 2017) from isochroman-1-one (300 mg, 2 mmol) and 2,4 dimethylpyrrole (0.627 mL, 6 mmol). Yield (240 mg, 35%), ^{1}H NMR (400 MHz, $CDCl_3$) δ 7.41-7.28 (m, 3H), 7.15 (m, 1H), 5.96 (s, 2H), 3.69 (t, *J* = 7.0 Hz, 2H), 2.75 (t, *J* = 7.0 Hz, 2H), 2.52 (s, 6H), 1.36 (s, 6H); ^{13}C NMR (75 MHz, , $CDCl_3$) δ 155.5, 142.9, 140.8, 136.1, 134.4, 131.2, 130.2, 129.3, 128.4, 127.3, 121.4, 61.2, 36.0, 14.6, 14.0. HRMS (ESI-TOF): calcd for $C_{21}H_{24}BF_2N_2O$: $[M+H]^+$ 369.19481, found: 369.19616.

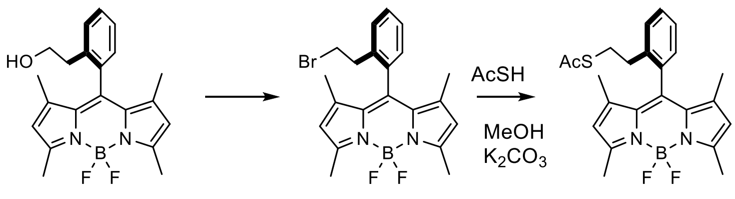

12 **12-Br** **12-SAc**

Scheme S1. Synthetic route to thioacetate **12S-Ac**

Compound 12-Br: To a stirred solution of compound **12** (155 mg, 0.42 mmol) in CH_2Cl_2 (20 mL) was added DMF (370 μL, 4.8 μmol). The solution was cooled to 0 °C and PBr_3 (60 μL, 0.63 mmol) was added. The mixture was allowed to warm to room temperature and then it was stirred for 30 min, and the solvent removed. Purification by flash chromatography (hexane – ethyl acetate 8:2 to 1:1) afforded compound **12-Br** (90 mg, 50%). ^{1}H NMR (400 MHz, $CDCl_3$) δ 7.46-7.19 (m, 5H), 5.99 (s, 2H), 3.49 (t, *J*= 6.9 Hz, 1H), 3.11 (t, *J*= 7.0 Hz, 1H), 2.56 (s, 6H), 1.37 (s, 6H); ^{13}C NMR (75 MHz, $CDCl_3$) δ 156.0, 142.8, 140.0, 135.9, 134.5, 131.5, 130.5, 129.4, 128.7, 128.0, 121.6, 35.9, 31.3, 14.7, 14.2. ^{19}F (376 MHz, $CDCl_3$) δ -147.1 (q, *J* = 33.2 Hz), -147.2 (q, *J* = 32.8 Hz). HRMS (ESI-TOF): calcd for $C_{21}H_{23}BBrF_2N_2$: $[M+H]^+$ 431.1104, found: 431.10932.). calcd for $C_{21}H_{22}BBrF_2N_2Na$: $[M+Na]^+$ 453.09235, found: 453.09091.

Compound 12-SAc: A solution of bromomethyl-BODIPY **12-Br** (80 mg, 0.19 mmol) in a 1:1 mixture of ethanol-dichloromethane (4 mL) was treated with K_2CO_3 (51 mg, 0.37 mmol) and thioacetic acid (16 μL, 0.41 mmol). The mixture was allowed to react at room temperature for 12 h and then it was diluted with dichloromethane, washed with $NaHCO_3$ and the organic layer was dried over sodium sulfate, filtered and concentrated. The residue was purified by flash chromatography (hexane-ethyl acetate 9:1) to give **12-SAc** (70 mg, 88%). ^{1}H NMR (500 MHz, $CDCl_3$) δ 7.48 – 7.31 (m, 3H), 7.20 – 7.14 (m, 1H), 5.97 (s, 2H), 3.02 (dd, *J* = 8.5, 6.6 Hz, 1H), 2.78 (dd, *J* = 8.7, 6.6 Hz, 1H), 2.55 (s, 6H), 2.28 (s, 3H), 1.35 (s, 6H). ^{13}C NMR (125 MHz, $CDCl_3$) δ 195.5, 155.8, 142.8, 140.4, 137.3, 134.3, 131.2, 129.9, 129.6, 128.5, 127.6, 121.5, 32.6, 30.7, 28.7, 14.7, 14.1. HRMS (ESI-TOF): calcd for $C_{23}H_{26}BF_2N_2OS$: $[M+H]^+$ 427.18257, found: 427.18267. calcd for $C_{23}H_{25}BF_2N_2NaOS$: $[M+Na]^+$ 449.16451, found: 449.16474.

Table S1. Photophysical properties of the bis-BODIPYs based on tetramethylated dipyrrin building blocks **2**, bearing different bridges (urea, thiourea, phosphonate, amine, acetylamine, amonium, and disulphur) as spacers and at different solvents.

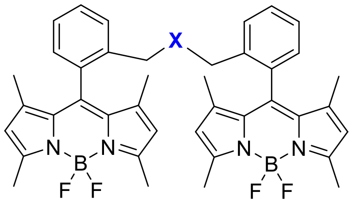

X	λ_{ab} (nm)	$\varepsilon_{max}\cdot 10^{-4}$ (M^{-1} cm^{-1})	λ_{fl} (nm)	ϕ	τ (ns)
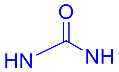 **4a** ACN	498.0	14.8	509.0	0.16	0.87
DMF	501.0	13.1	512.5	0.36	2.03(50%) – 2.87(50%)
EtOH	500.0	15.7	511.5	0.25	0.68(13%) – 2.14(87%)
c-hex	502.5	13.4	515.0	0.83	5.66
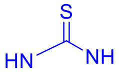 **4b** ACN	499.0	12.6	512.0	0.08	0.32(46%) – 0.79(54%)
DMF	501.5	11.3	512.5	0.18	0.44(39%) – 1.82(61%)
EtOH	501.5	14.0	511.0	0.17	0.17(25%) – 1.45(75%)
c-hex	503.5	10.0	517.5	0.51	1.86(46%) – 6.29(54%)
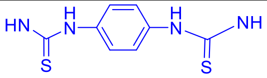 **4c** ACN	500.0	14.5	508.0	0.06	0.04(70%)-0.38(18%)-2.09(12%)
DMF	502.5	14.0	512.5	0.12	0.31(33%)-1.47(36%)-4.88(31%)
EtOH	502.0	15.0	510.5	0.11	0.23(56%)-1.19(25%)-5.38(19%)
c-hex	504.5	16.0	511.0	0.40	0.22(31%)-1.86(12%)-5.12(57%)
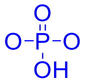 **4e** ACN	499.0	13.2	511.0	0.03	0.03(62%) – 0.36(38%)
DMF	502.5	14.0	515.0	0.08	0.69(96%) – 4.60(4%)
EtOH	501.0	13.5	513.0	0.12	1.18(96%) – 4.79(4%)
Et_2O*	500.5	13.3	514.0	0.43	0.55(28%) – 6.41(72%)
NH **4f** ACN	496.0	13.8	512.0	0.02	0.10(92%) – 0.35(8%)
DMF	500.5	13.7	515.5	0.04	0.13(83%) – 0.67(17%)
EtOH	498.5	14.0	514.5	0.09	0.40(80%) – 1.87(20%)
c-hex	501.5	14.0	517.5	0.50	2.34(56%) – 6.59(44%)
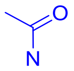					

**4g** ACN	496.5	12.1	515.0	0.02	0.09(94%) – 0.41(6%)
DMF	499.5	12.9	517.0	0.04	0.12(76%) – 0.40(24%)
EtOH	498.0	12.0	520.5	0.05	0.12(51%) – 0.92(49%)
c-hex	499.0	12.3	522.5	0.89	7.51
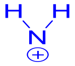 **4h** ACN	499.5	10.4	511.5	0.02	0.08(97%) – 0.40(3%)
DMF	503.0	10.7	513.5	0.03	0.13(92%) – 0.51(8%)
EtOH	502.0	11.8	511.5	0.10	0.46(47%) – 1.27(53%)
c-hex	504.5	11.5	513.0	0.90	4.98
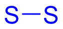 **4i** ACN	499.5	17.0	517.5	0.03	0.15(55%)–0.32(44%)–4.56(1%)
DMF	502.5	21.2	518.5	0.06	0.53(97%) – 3.73 (3%)
EtOH	501.0	20.9	519	0.09	0.27 (29%)–0.90(69%)–4.91(%2)
Et_2O*	503.0	21.0	524	0.85	6.76
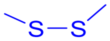 **4j** ACN	499.5	17.6	512.5	0.08	0.42(93%) – 5.61(7%)
DMF	502.0	17.7	515.5	0.09	0.39(37%)–0.92(62%)–4.16(1%)
EtOH	501.0	17.5	516.5	0.15	1.08(52%) – 1.92(48%)
c-hex	503.5	14.5	518.0	0.92	6.68

*not soluble in cyclohexane. Diethyleter (Et_2O) was taken as a solvent of low polarity but able to dissolve the dye

c-hex: cyclohexane; EtOH: ethanol; DMF: dimethylformamide; ACN: acetonitrile

Absorption (λ_{ab}) and fluorescence (λ_{fl}) wavelength, molar absorption at the maximum (ε_{max}), fluorescence quantum yield (ϕ) and lifetime (τ)

Table S2. Photophysical properties of the bis-BODIPYs based on fully alkylated dipyrrin building blocks **3**, bearing different bridges (urea, thiourea, phosphonate, amine, acetylamine, disulphur) as spacers and at different solvents.

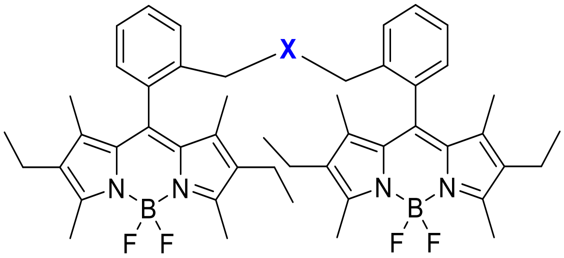

X	λ_{ab} (nm)	$\varepsilon_{max}\cdot10^{-4}$ (M^{-1} cm^{-1})	λ_{fl} (nm)	ϕ	τ (ns)
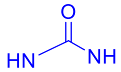 **5a** ACN	522.5	14.2	537.0	0.22	0.05(48%) – 1.47(52%)
DMF	524.5	14.2	539.0	0.66	4.08
EtOH	524.0	13.0	537.5	0.31	0.49(36%) – 3.42(64%)
c-hex	525.5	16.2	540.5	0.98	6.76
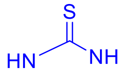 **5b** ACN	522.0	17.7	537.0	0.13	1.26(94%) – 5.63(6%)
DMF	524.5	17.5	539.5	0.43	2.61(26%) – 4.21(74%)
EtOH	524.0	18.2	538.5	0.23	0.56(26%)–2.85(66%)–5.61(8%)
c-hex	525.5	15.5	540.0	0.76	6.79
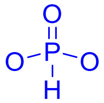 **5d** ACN	523.5	22.6	539.5	0.16	0.82(85%) – 6.44(15%)
DMF	526.0	22.8	542.5	0.24	1.42(81%) – 5.71(19%)
EtOH	525.0	23.6	541.0	0.31	1.89(71%) – 6.40(29%)
c-hex	526.5	22.6	542.5	0.79	7.28
NH **5f** ACN	521.5	13.3	538.5	0.11	0.44(75%)–1.40(16%)–6.39(10%)
DMF	524.5	14.7	541.5	0.14	0.33(25%)–1.48(58%)–5.24(17%)
EtOH	523.5	13.9	539.0	0.29	2.21(73%) – 6.65(27%)
c-hex	526.0	15.9	540.5	0.77	7.22
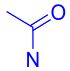 **5g** ACN	523.5	13.7	540.5	0.09	0.21(50%) – 1.49(50%)
DMF	525.5	14.2	542.5	0.19	0.12(40%)–2.30(52%)–5.17(8%)
EtOH	524.5	10.1	541.5	0.22	0.13(21%)–0.87(43%)–4.30(31%)

c-hex	524.5	13.2	544.0	0.90	8.37
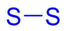 **5i** ACN	524.0	10.3	540.5	0.10	0.80(95%) – 6.20(5%)
DMF	526.5	10.9	543.0	0.18	1.52(93%) – 5.22(7%)
EtOH	525.5	11.4	542.0	0.33	2.31(35%) – 4.07(65%)
c-hex	527.0	19.6	542.5	0.84	7.04

c-hex: cyclohexane; EtOH: ethanol; DMF: dimethylformamide; ACN: acetonitrile

Absorption (λ_{ab}) and fluorescence (λ_{fl}) wavelength, molar absorption at the maximum (ε_{max}), fluorescence quantum yield (ϕ) and lifetime (τ)

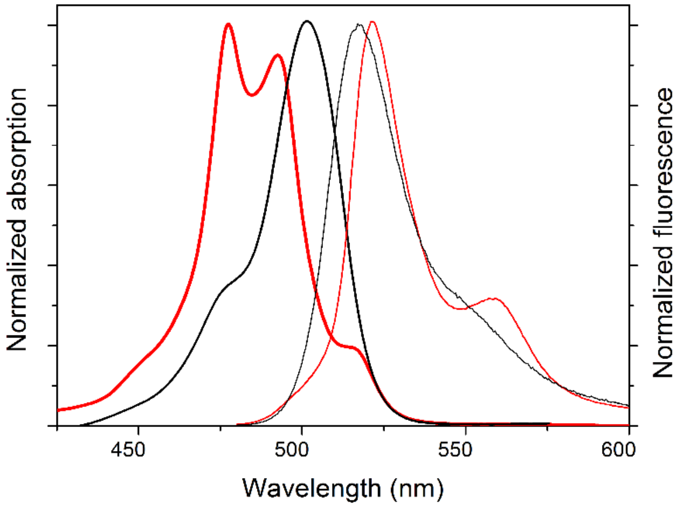

Figure S1. Normalized absorption and fluorescence spectra of the ether-bridged bisBODIPY **4k** and its corresponding analog **4f** with amino-bridge (dotted line) in cyclohexane.

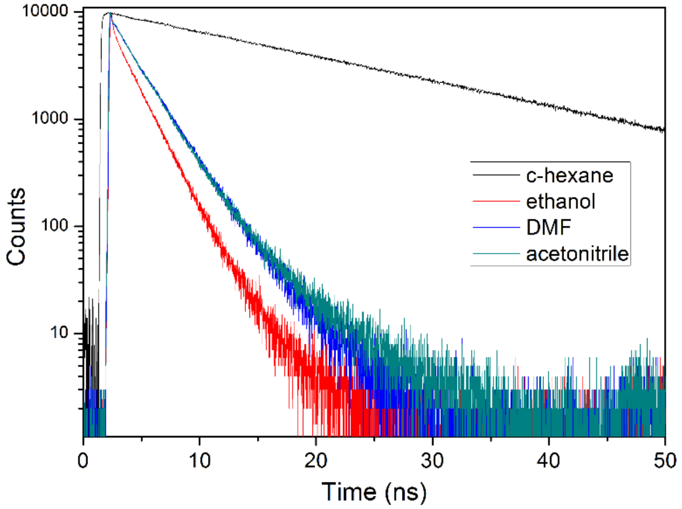

Figure S2. Fluorescence decay curves of **4k** dyad as the solvent polarity increases.

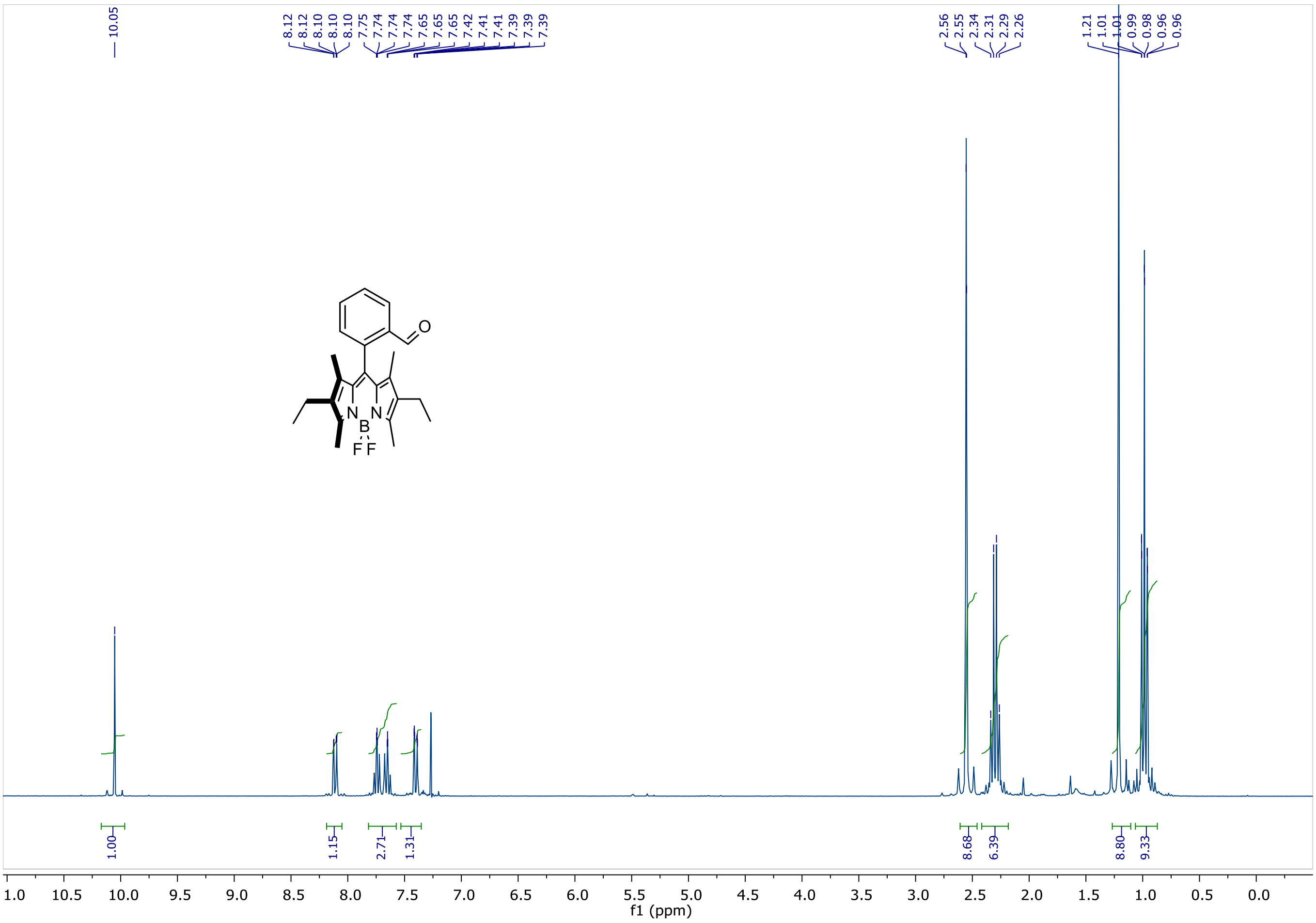

Figure S3. ^{1}H NMR spectrum of compound **6b**

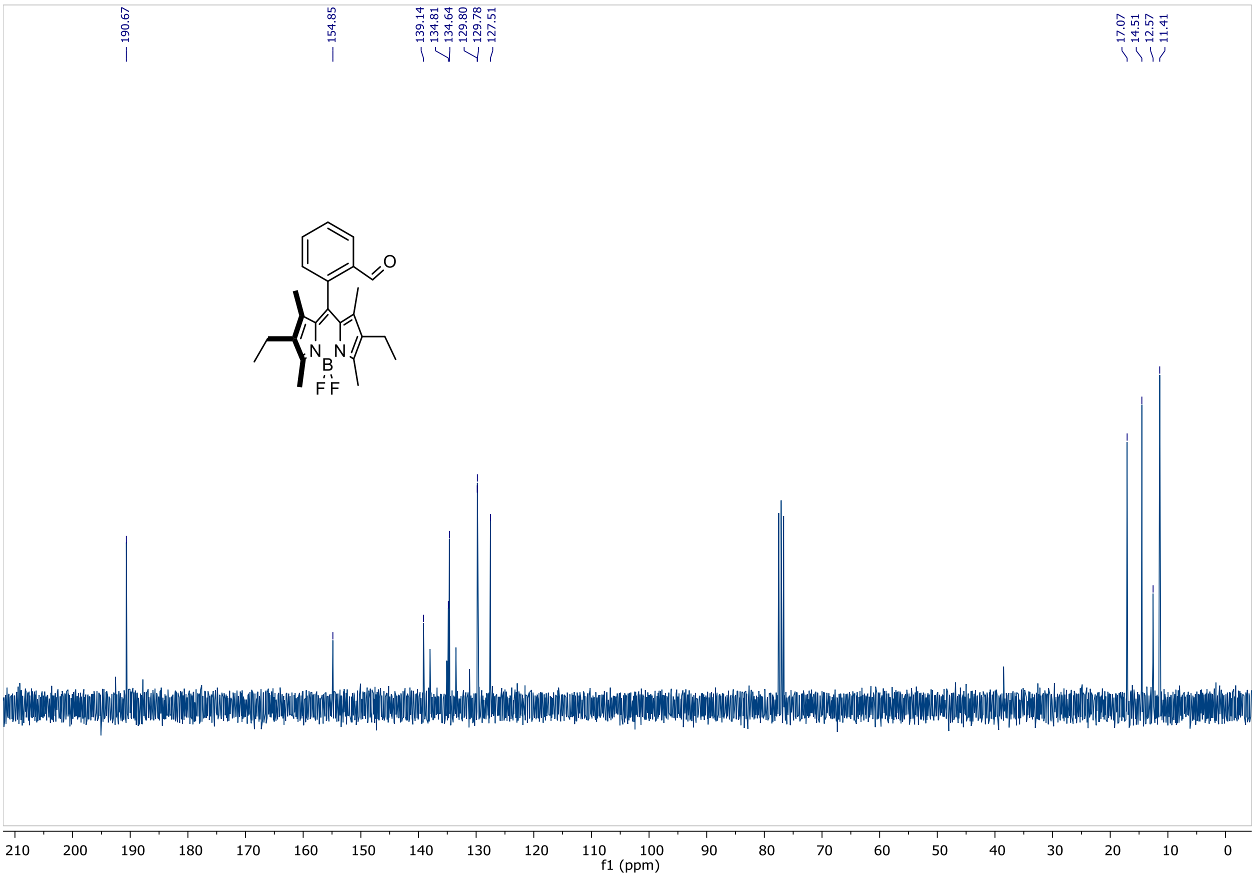

Figure S4. ^{13}C NMR spectrum of compound **6b**

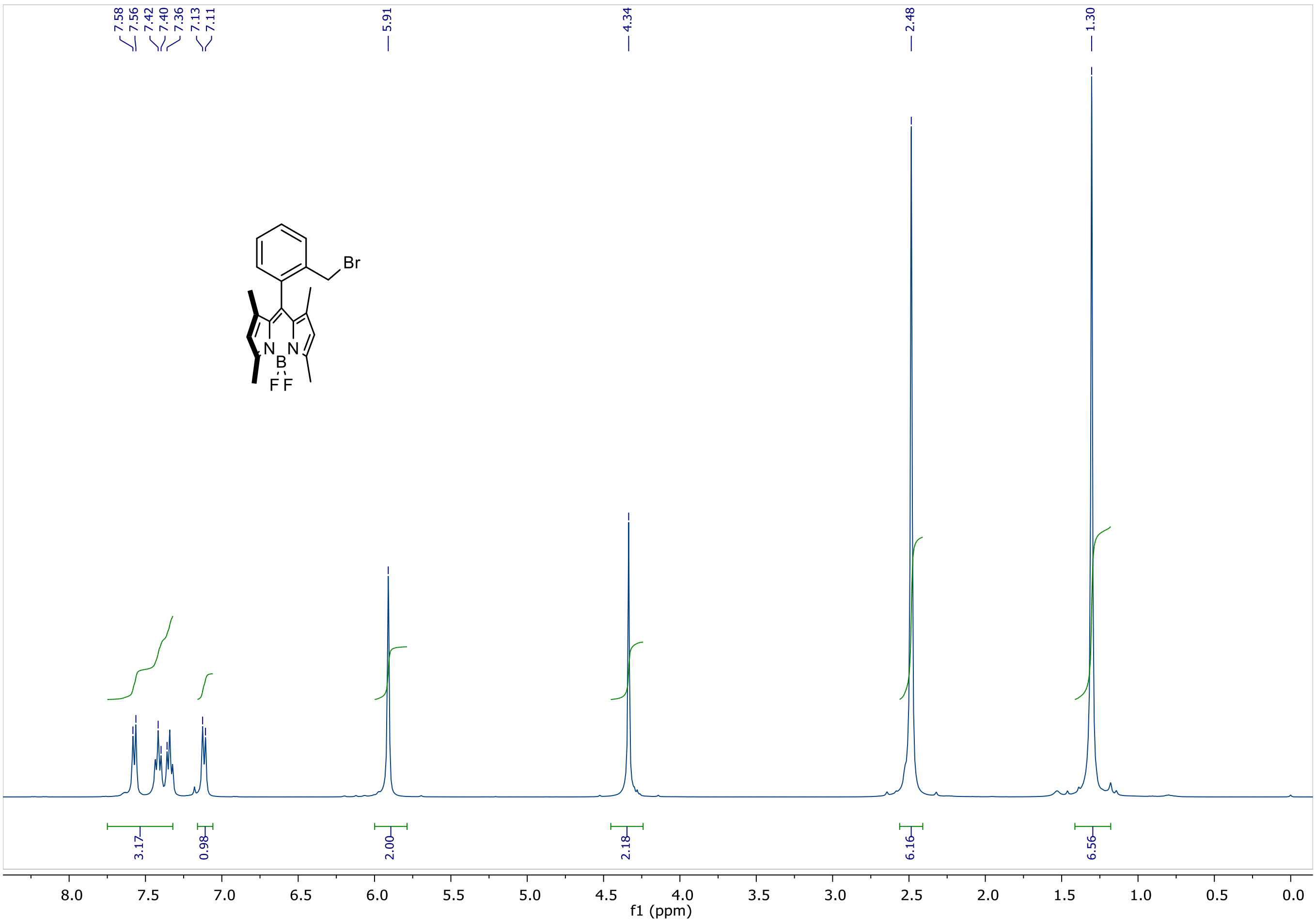

Figure S5. ^{1}H NMR spectrum of compound **8a**

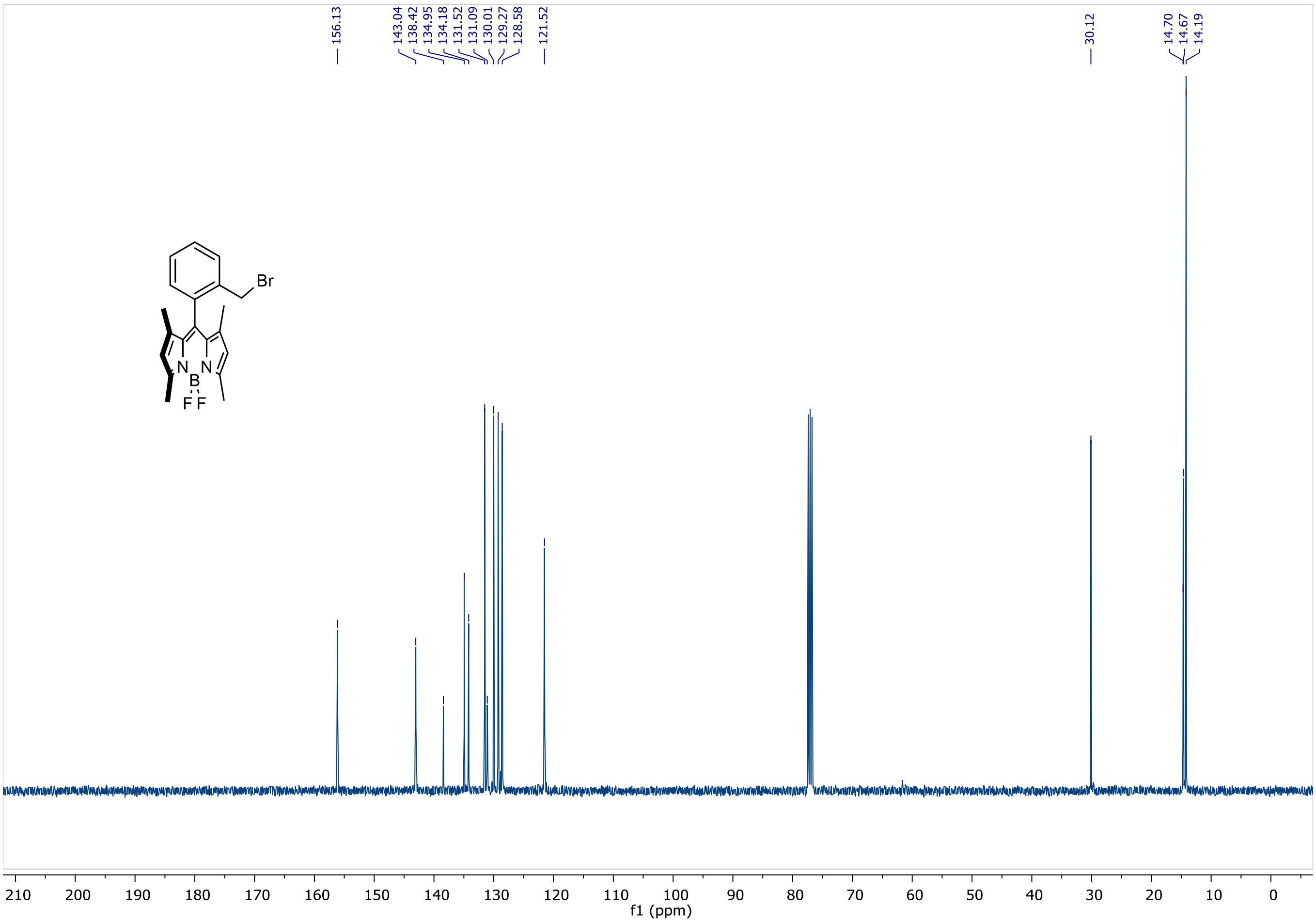

Figure S6. ^{13}C NMR spectrum of compound **8a**

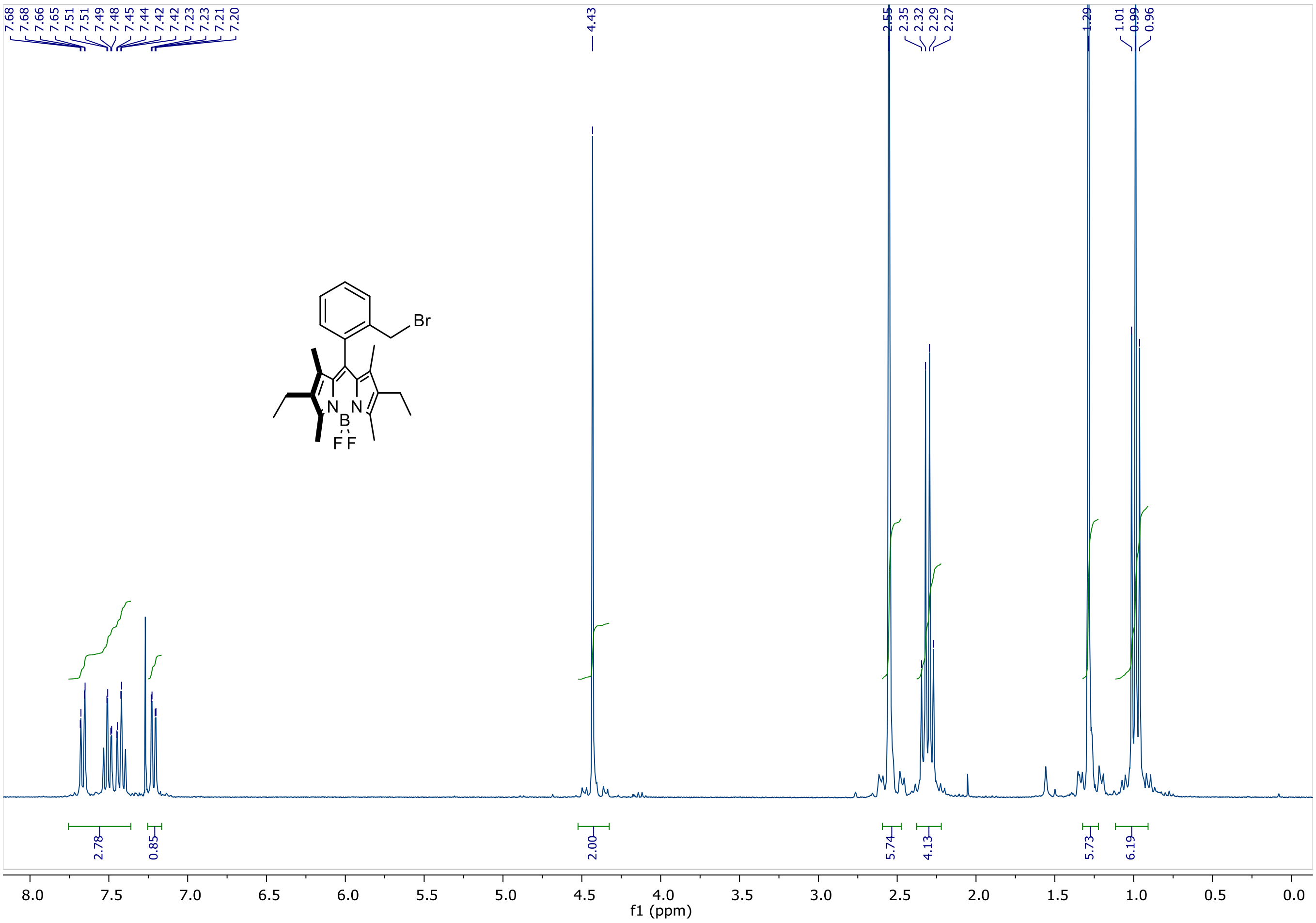

Figure S7. ^{1}H NMR spectrum of compound **8b**

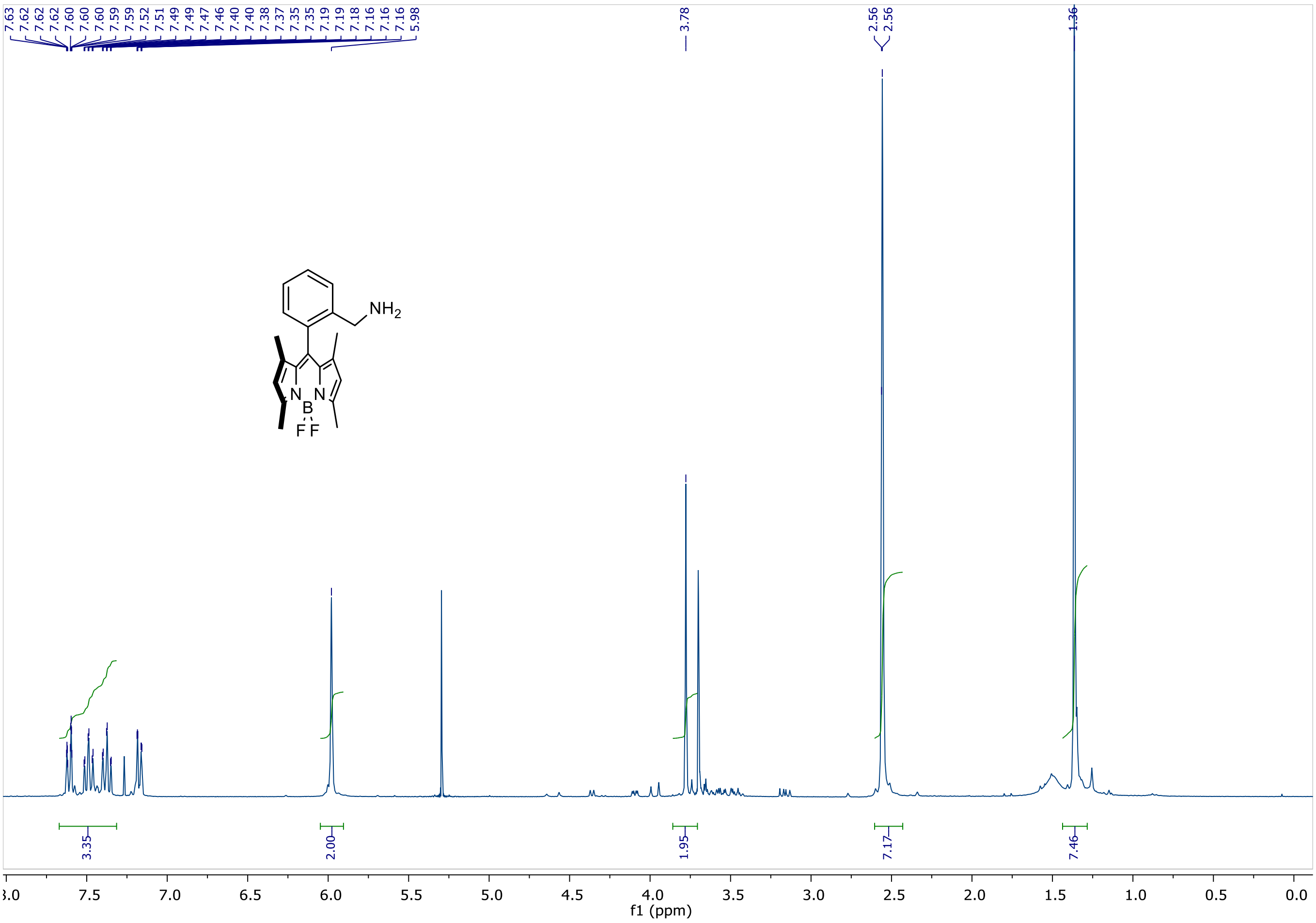

Figure S8. ^{1}H NMR spectrum of compound **9a**

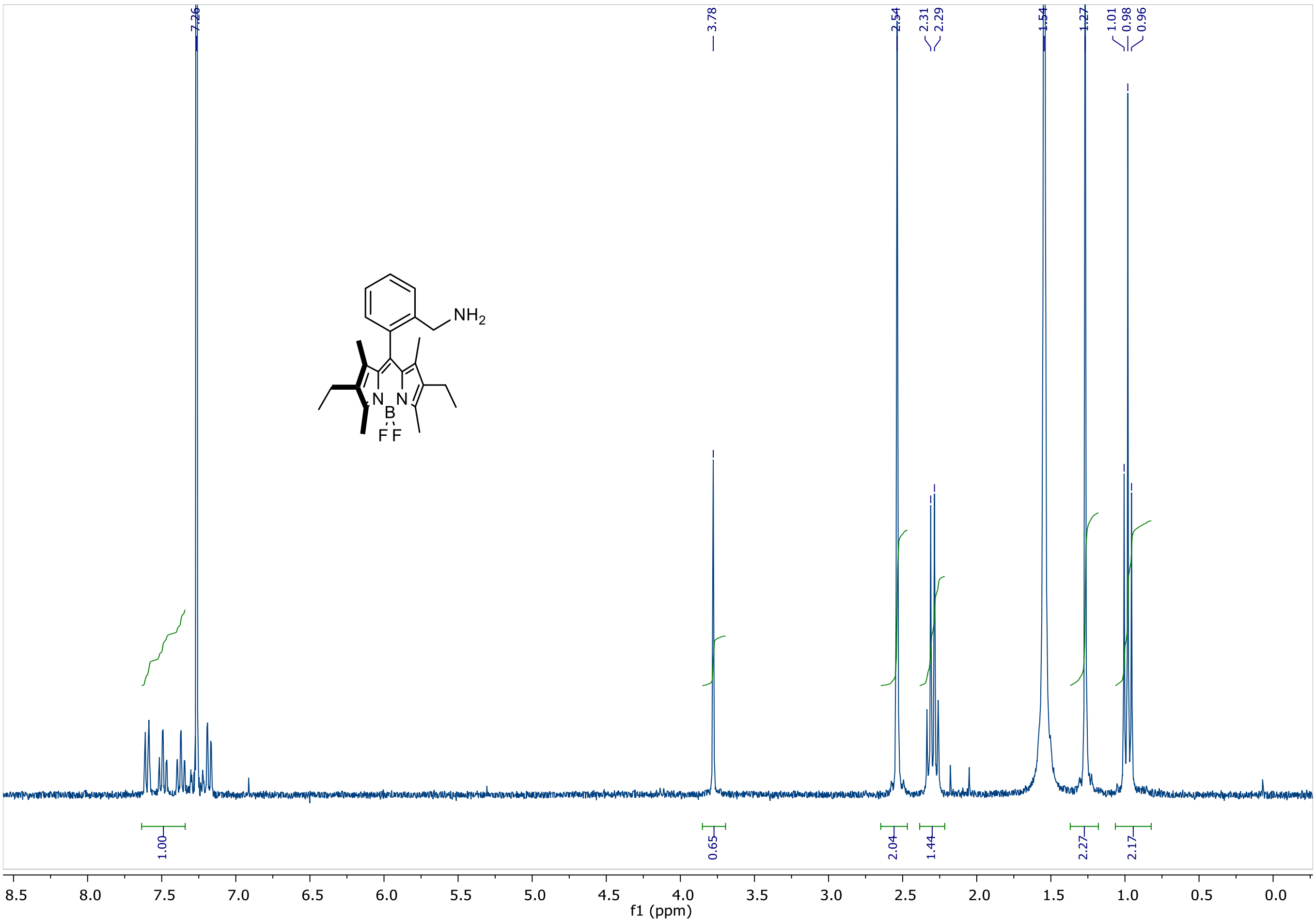

Figure S9. ^{1}H NMR spectrum of compound **9b**

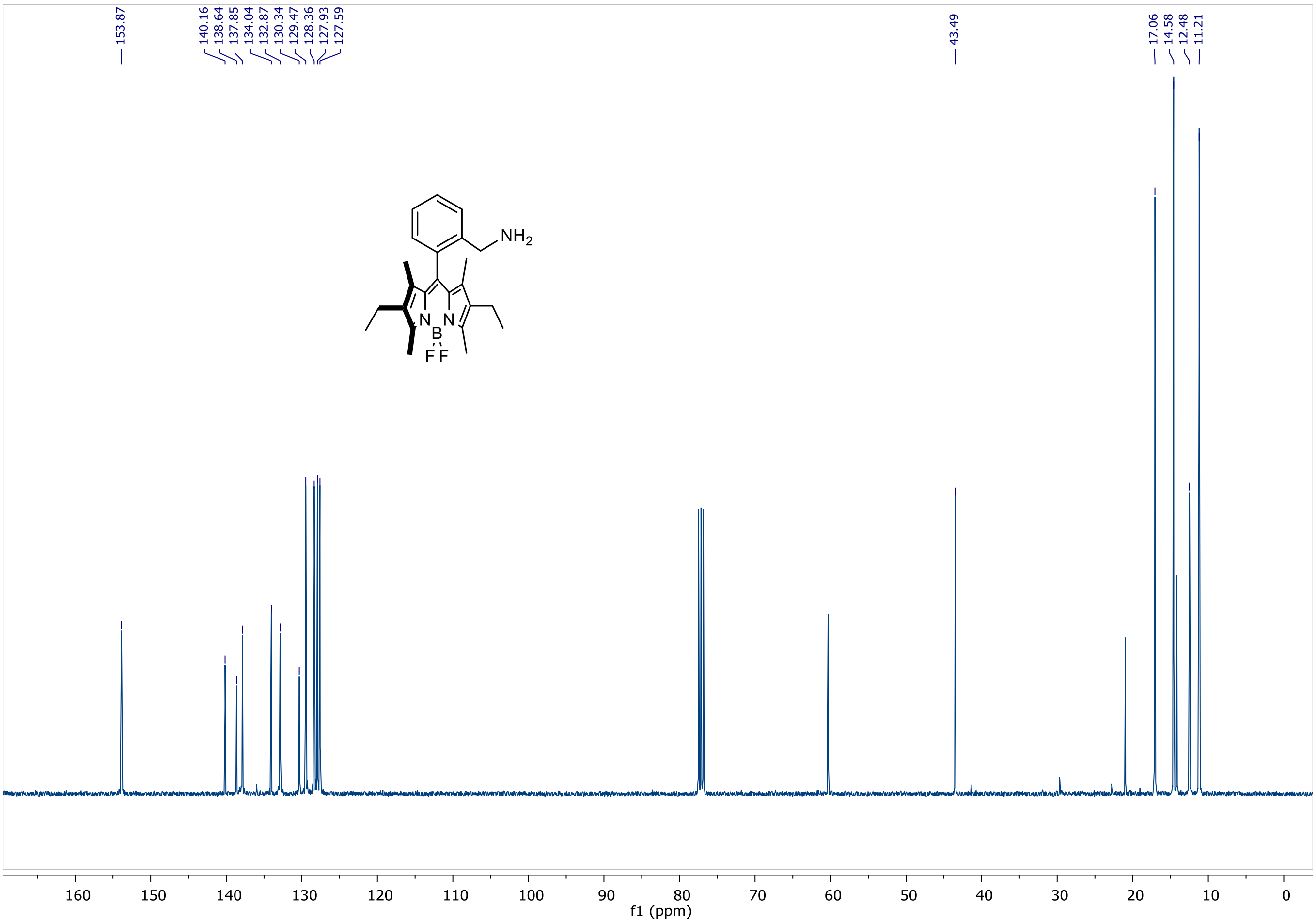

Figure S10. ^{13}C NMR spectrum of compound **9b**

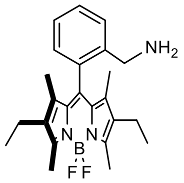

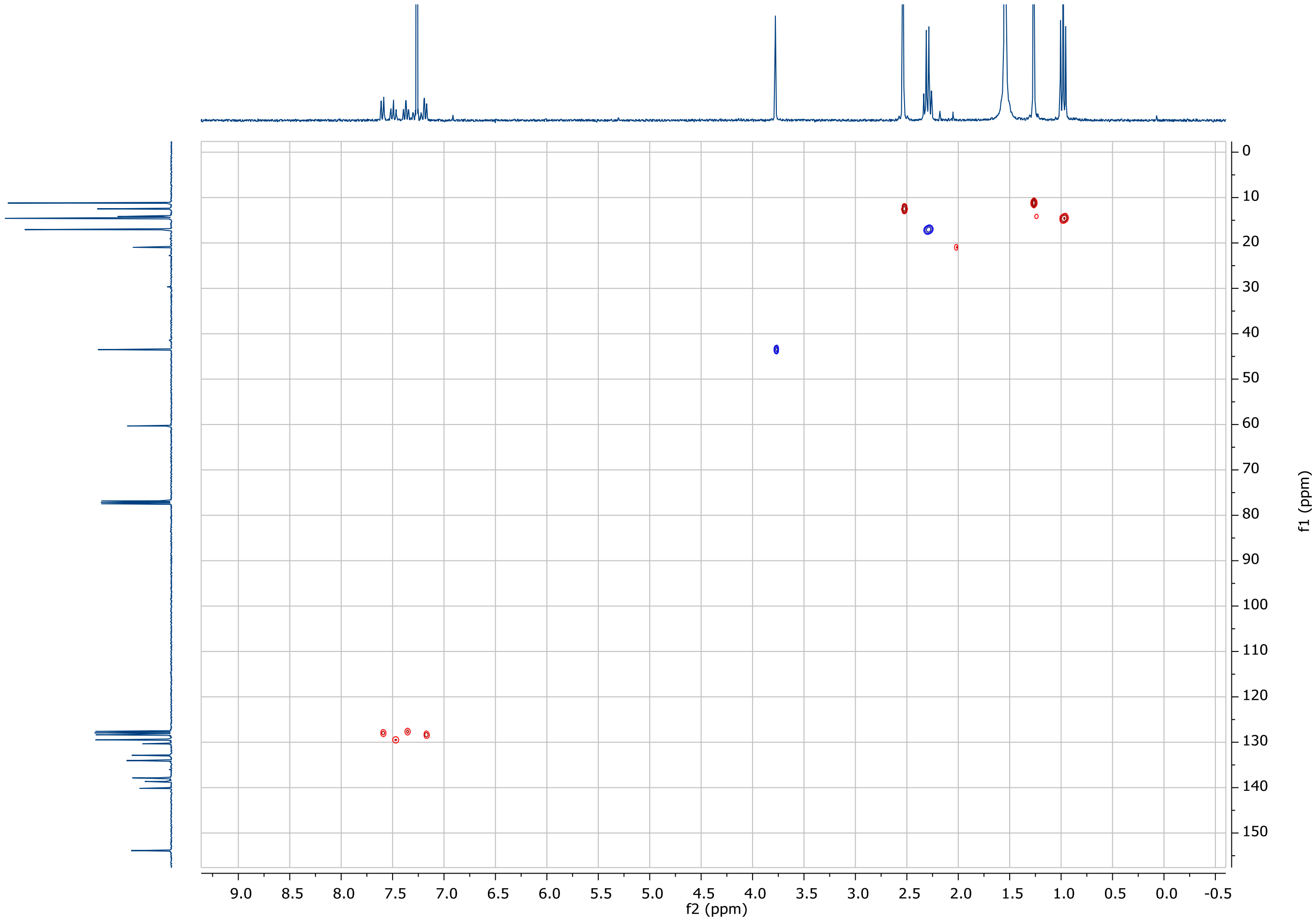

Figure S11. HSQC NMR spectrum of compound **9b**

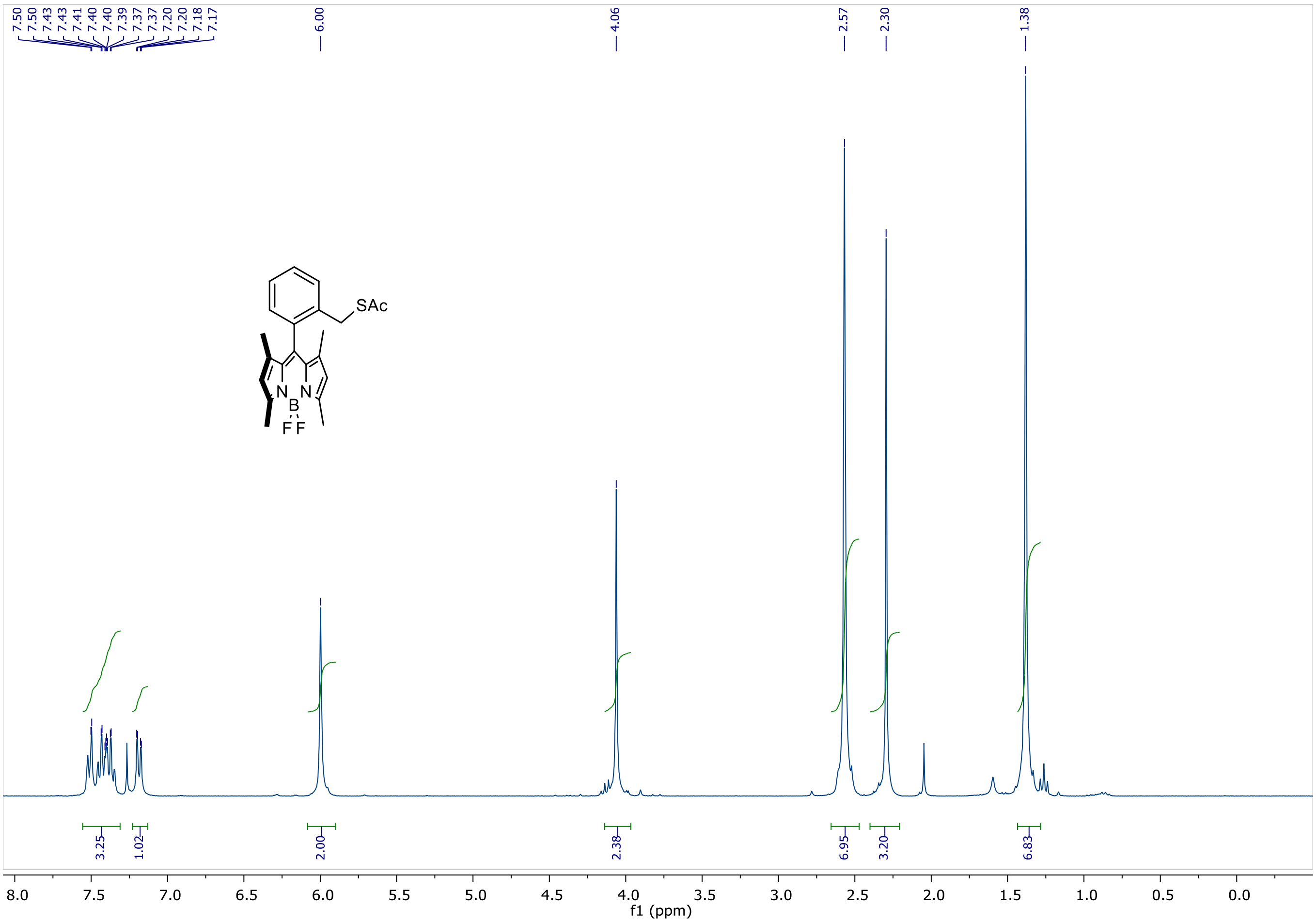

Figure S12. ^{1}H NMR spectrum of compound **11a**

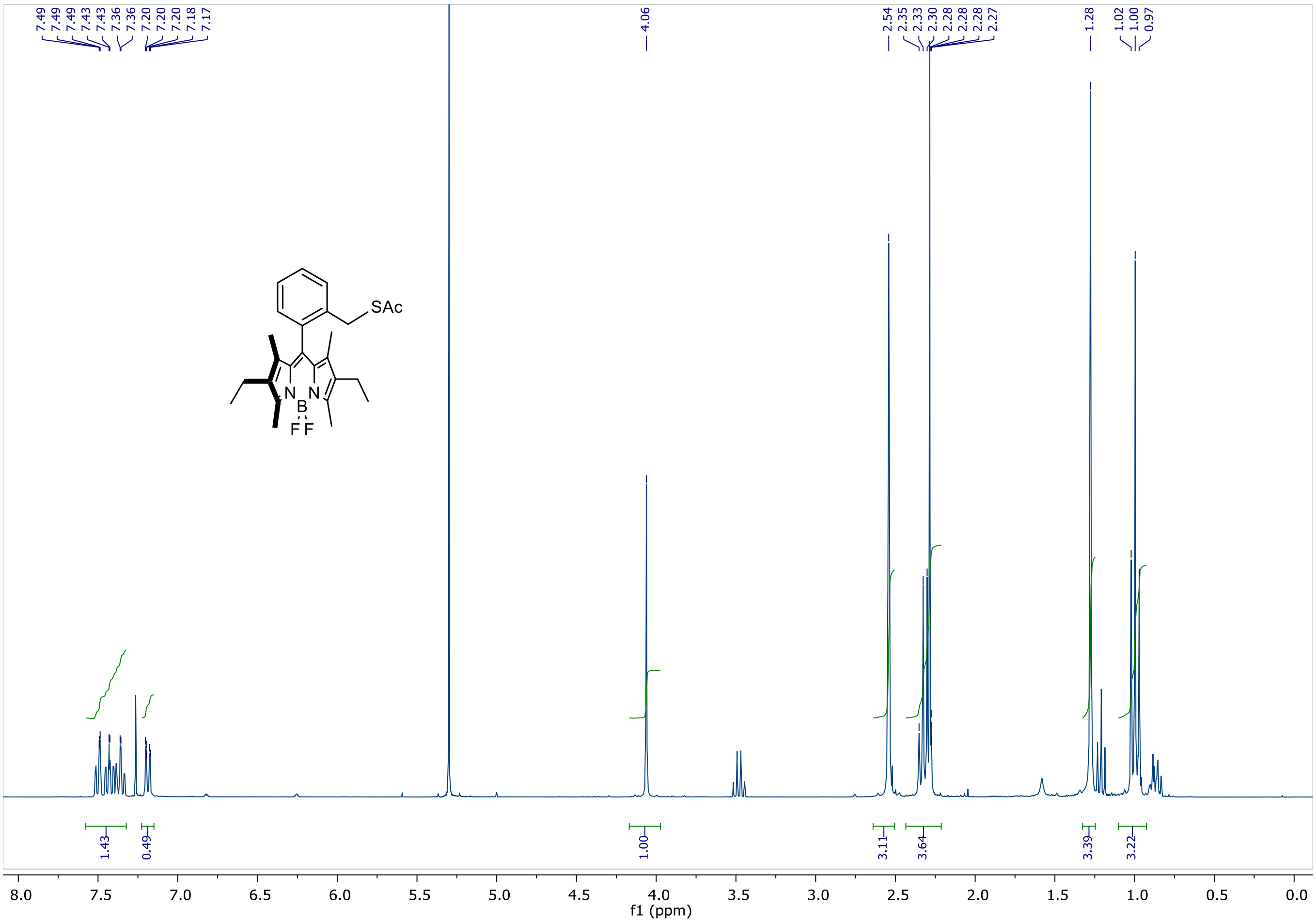

Figure S13. ^{1}H NMR spectrum of compound **11b**

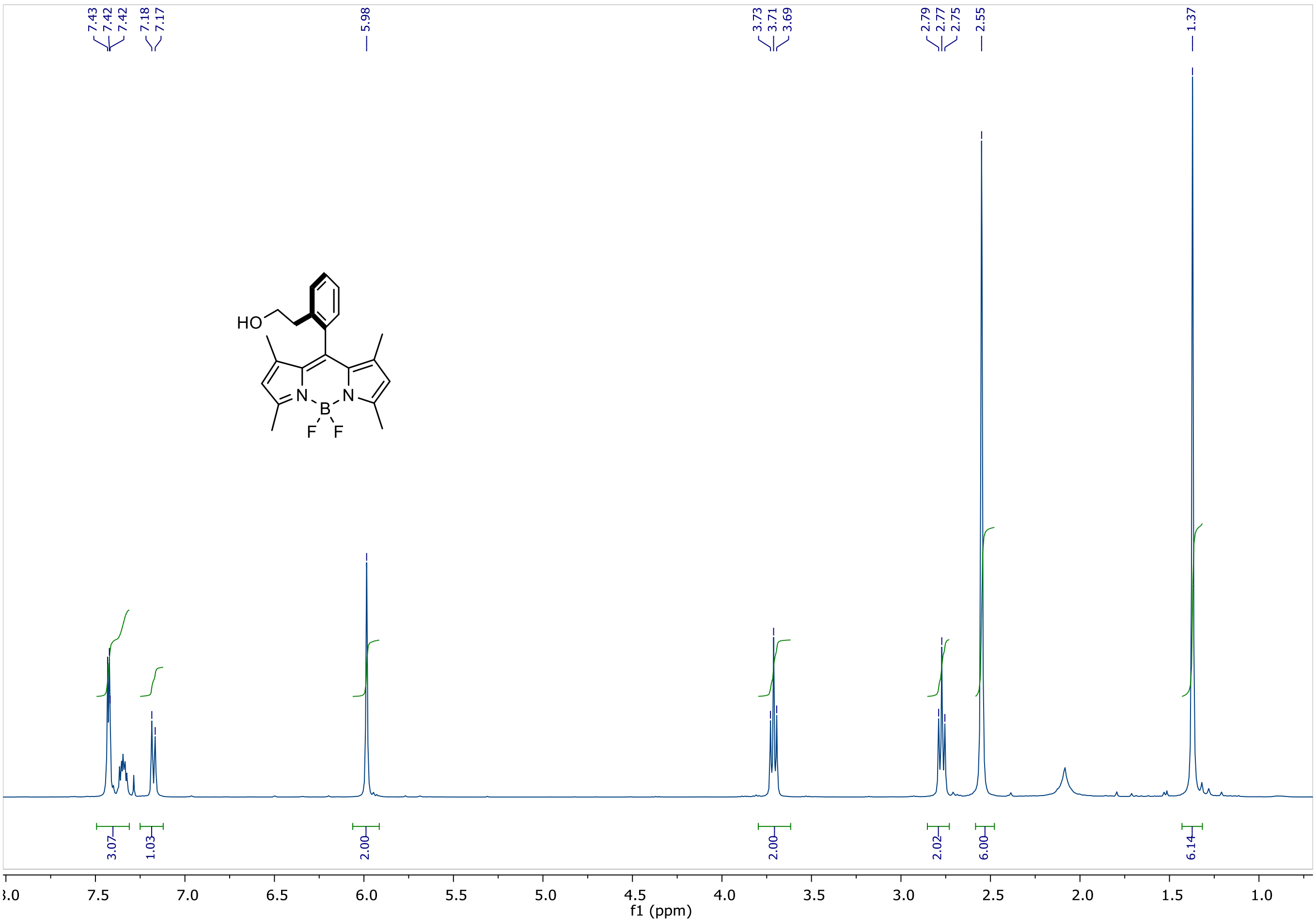

Figure S14. ^{1}H NMR spectrum of compound **12**

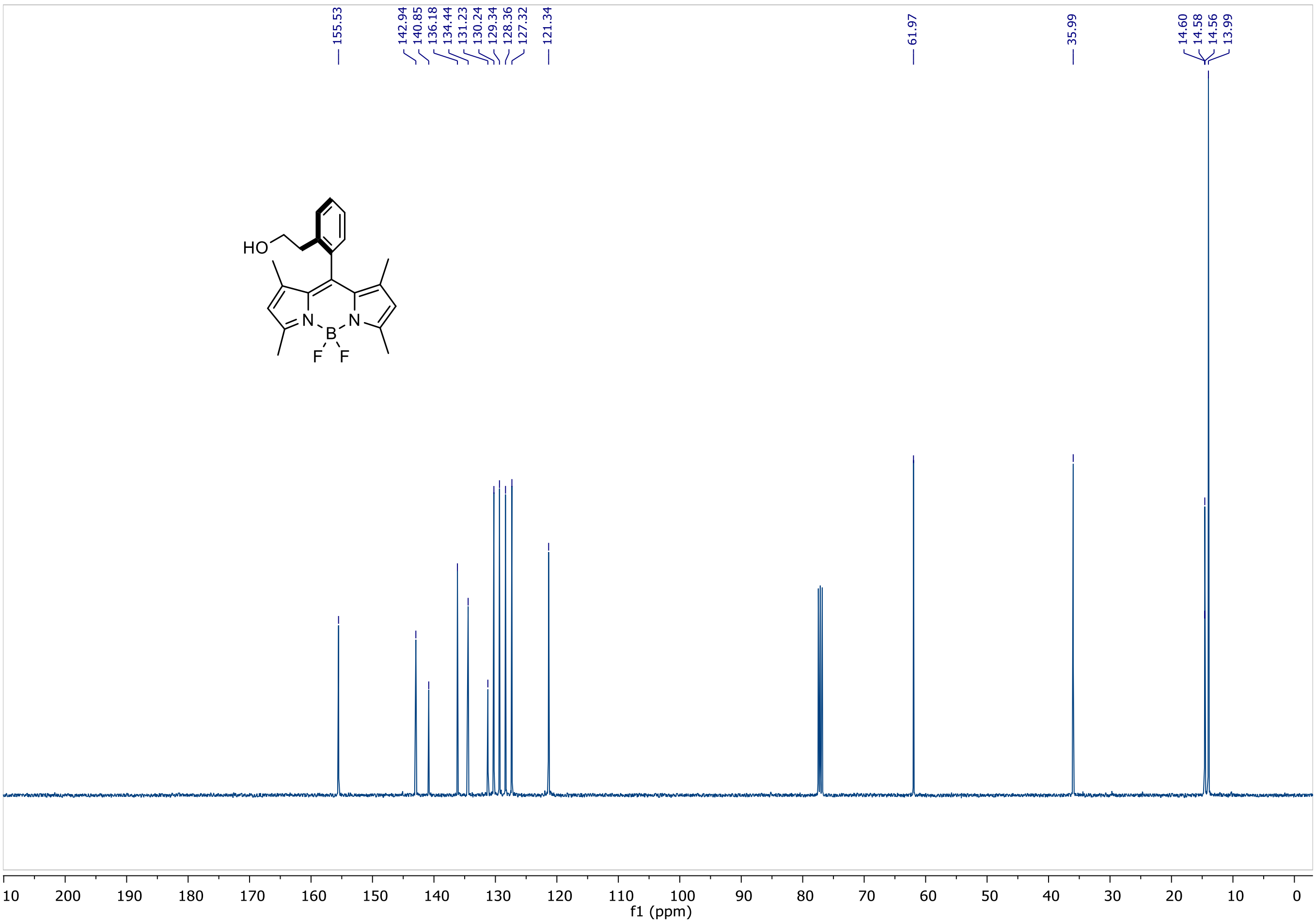

Figure S15. ^{13}C NMR spectrum of compound **12**

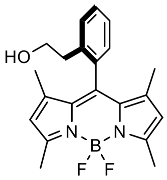

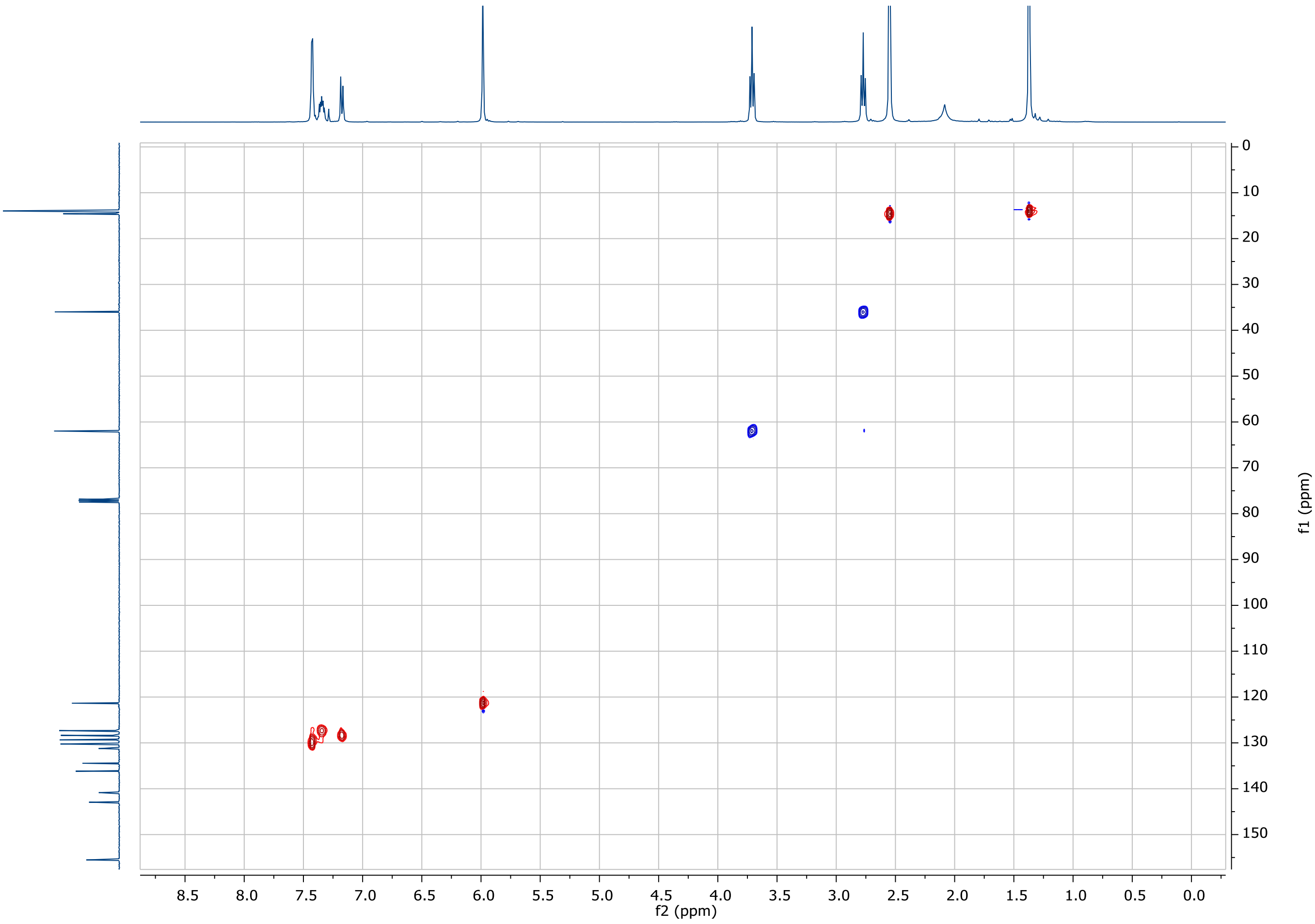

Figure S16. HSQC NMR spectrum of compound **12**

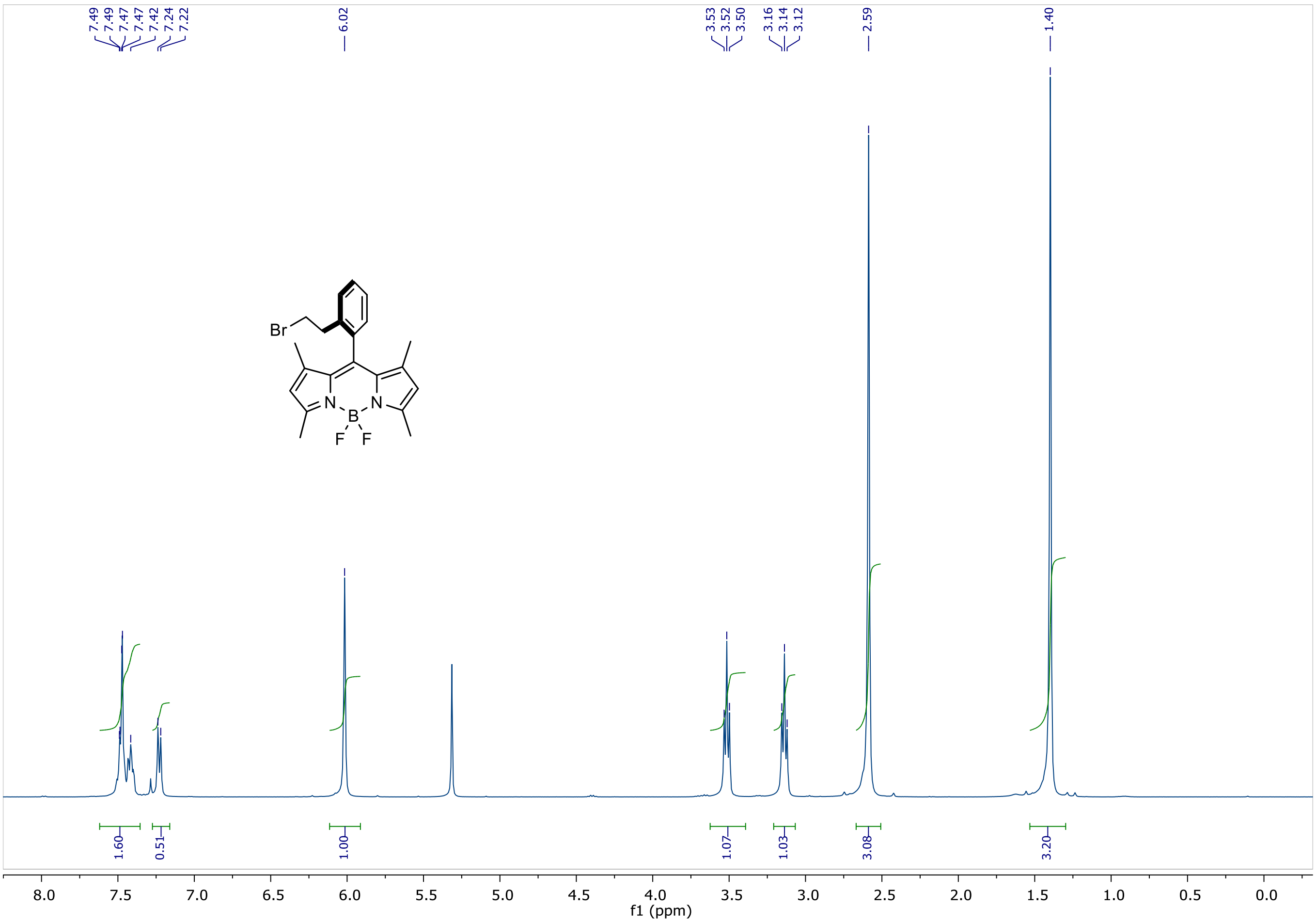

Figure S17. ^{1}H NMR spectrum of compound **12-Br**

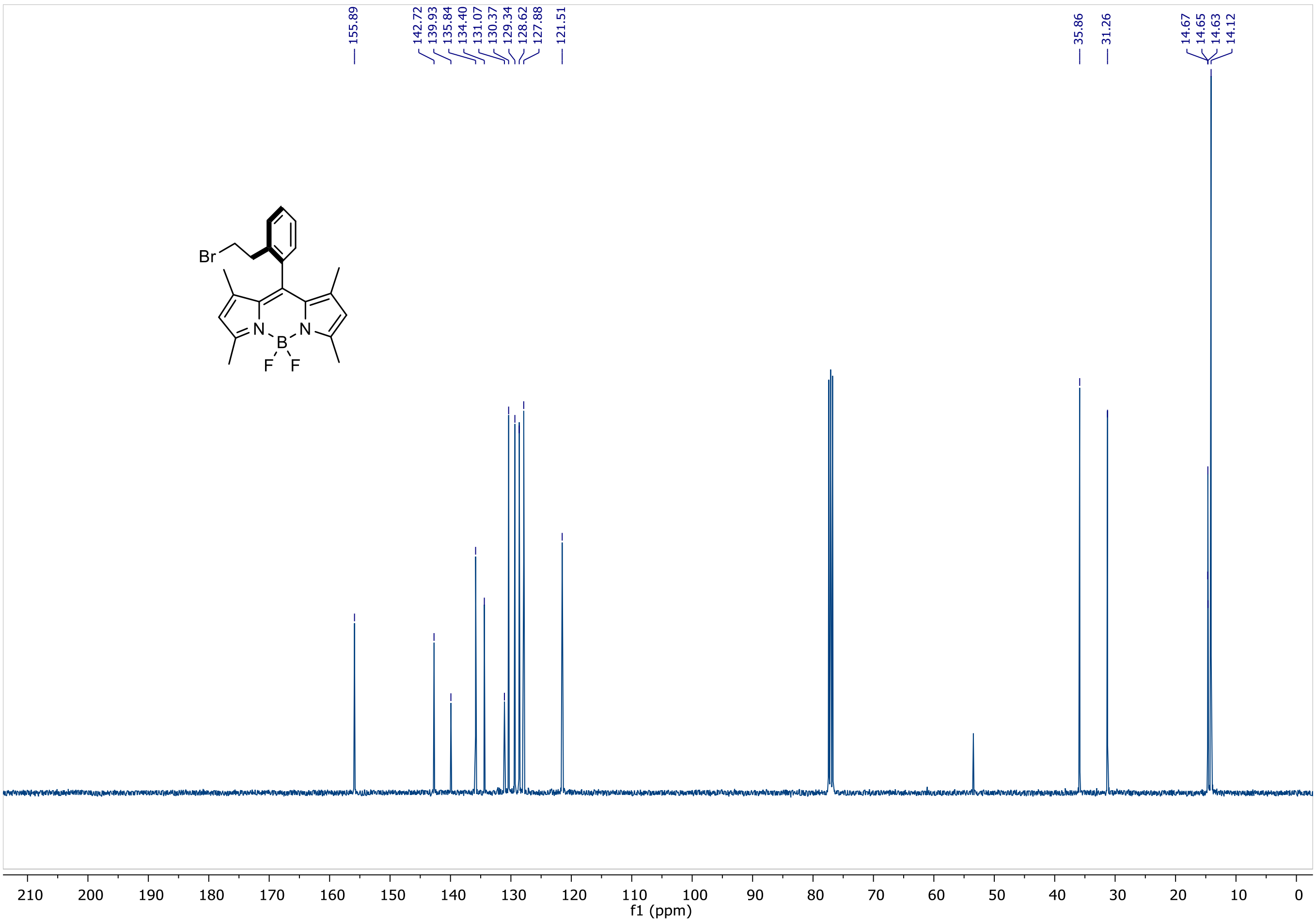

Figure S18. ^{13}C NMR spectrum of compound **12-Br**

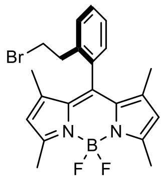

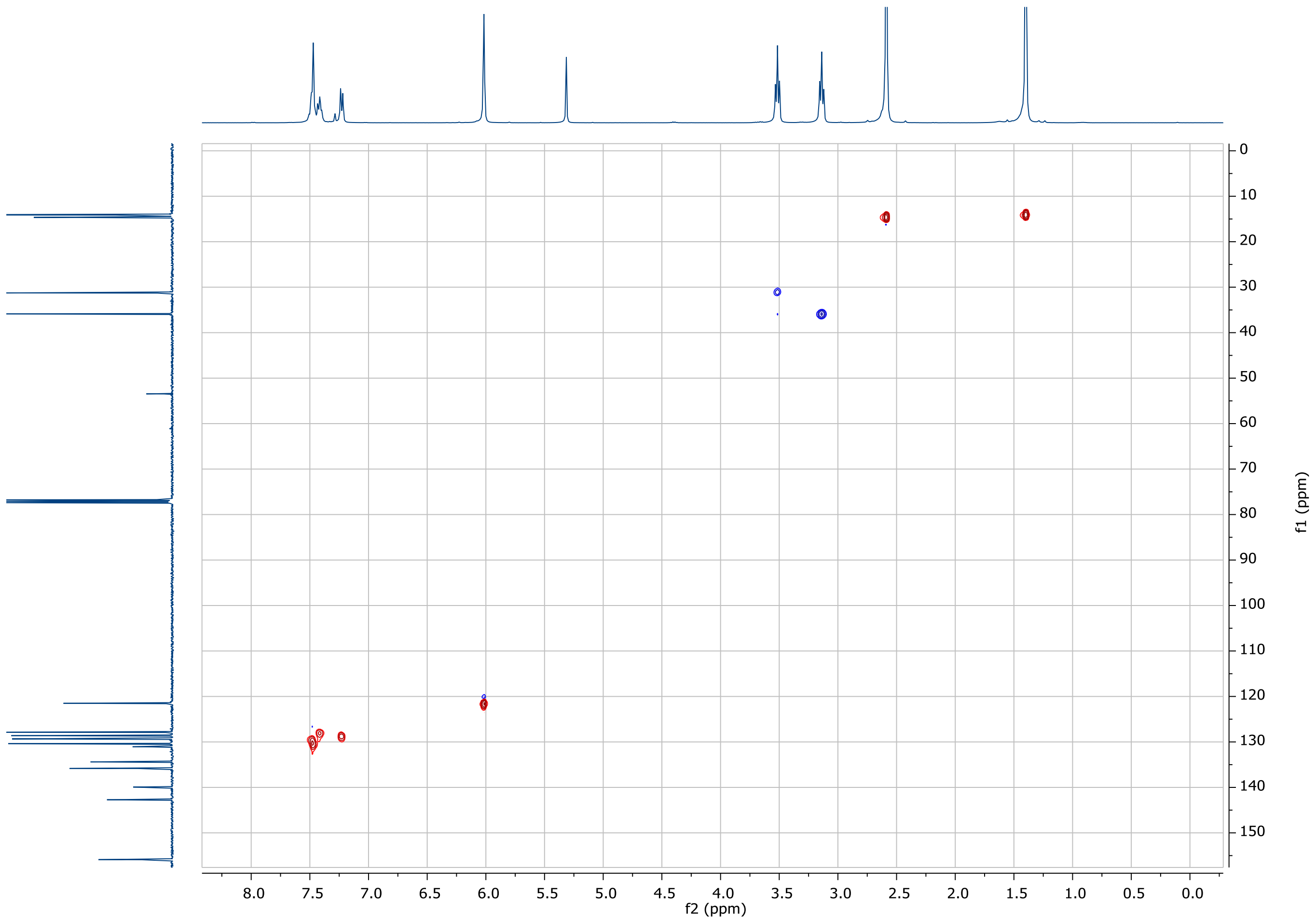

Figure S19. HSQC NMR spectrum of compound **12-Br**

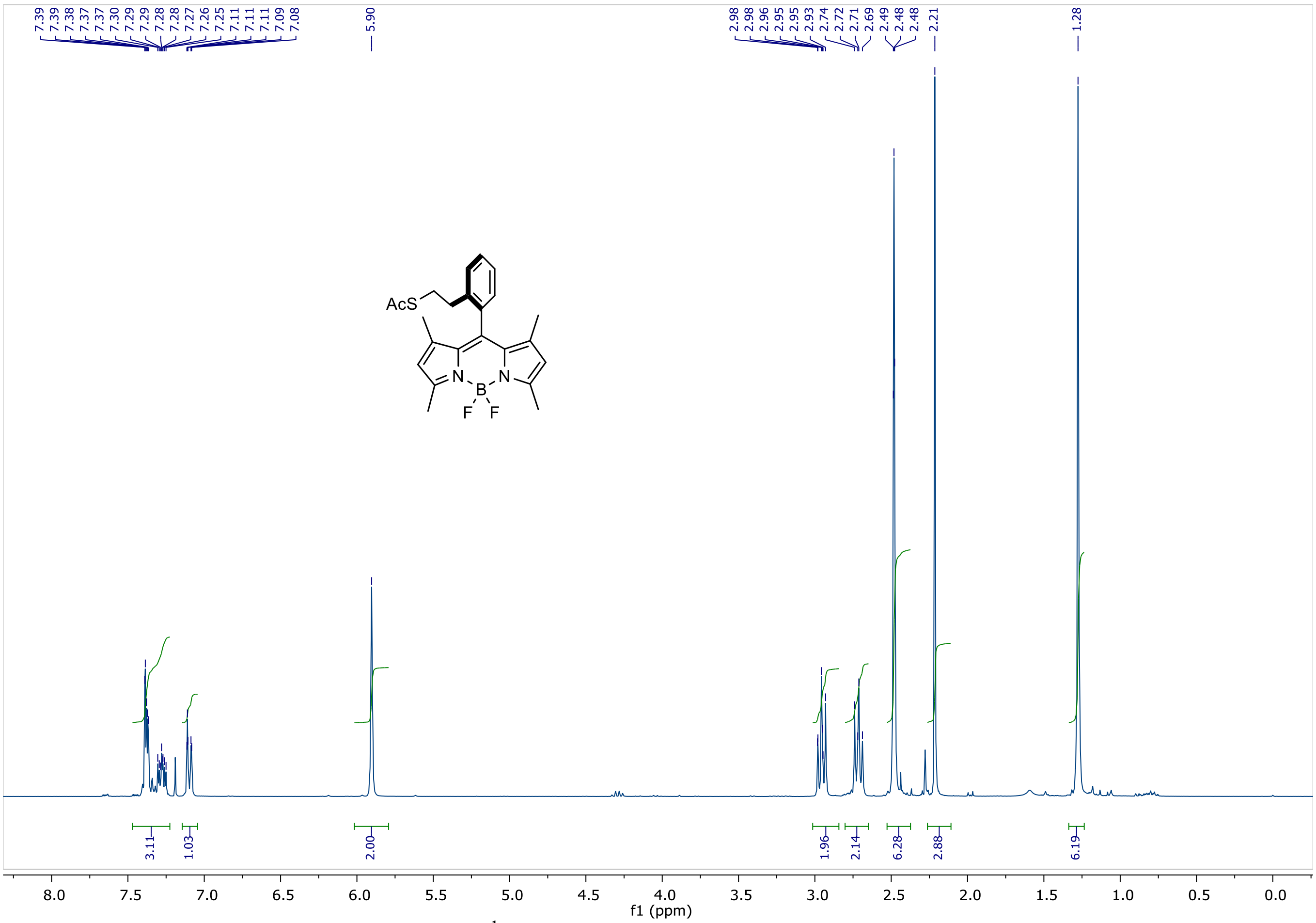

Figure S20. ^{1}H NMR spectrum of compound **12-SAc**

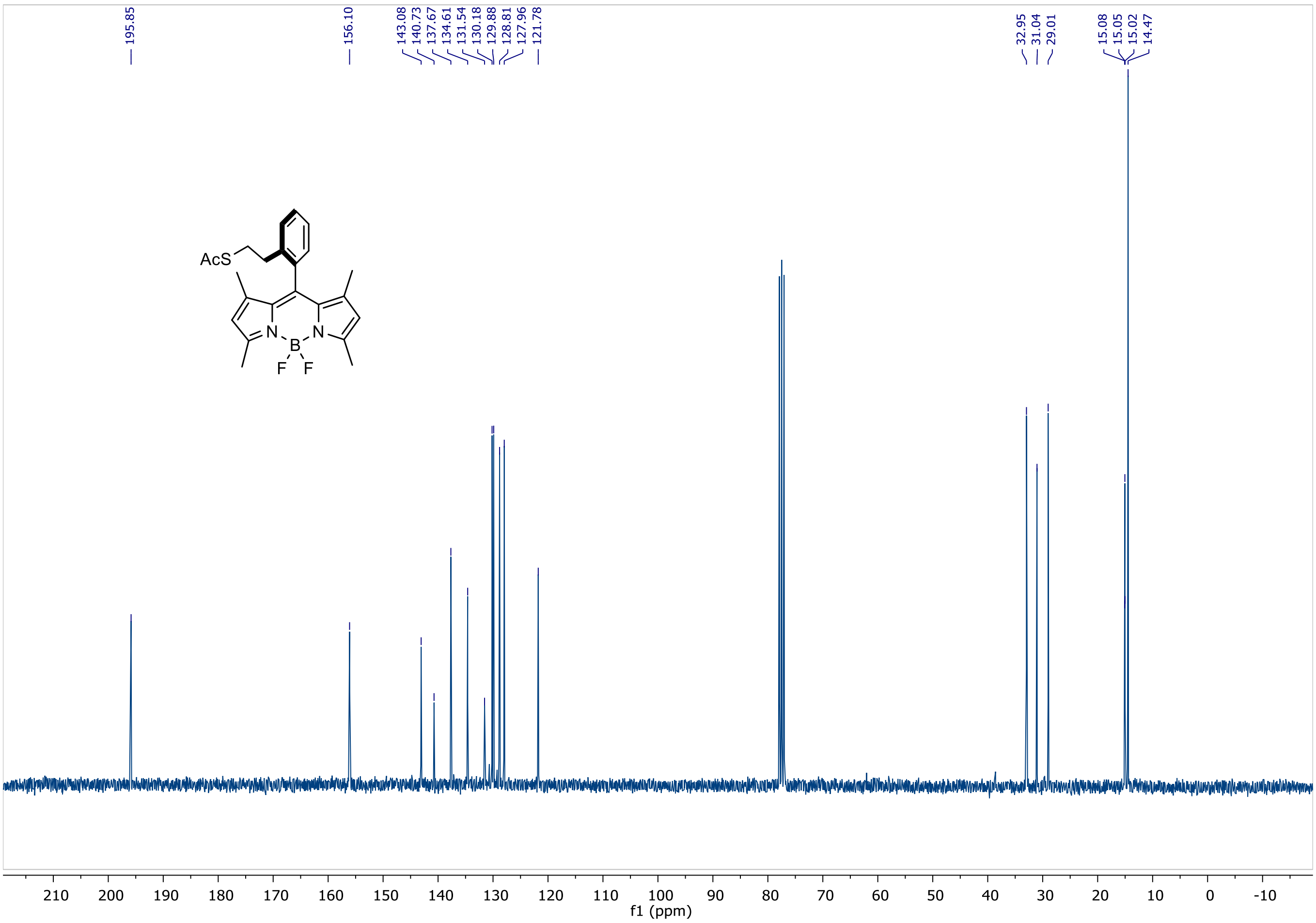

Figure S21. ^{13}C NMR spectrum of compound **12-Sac**

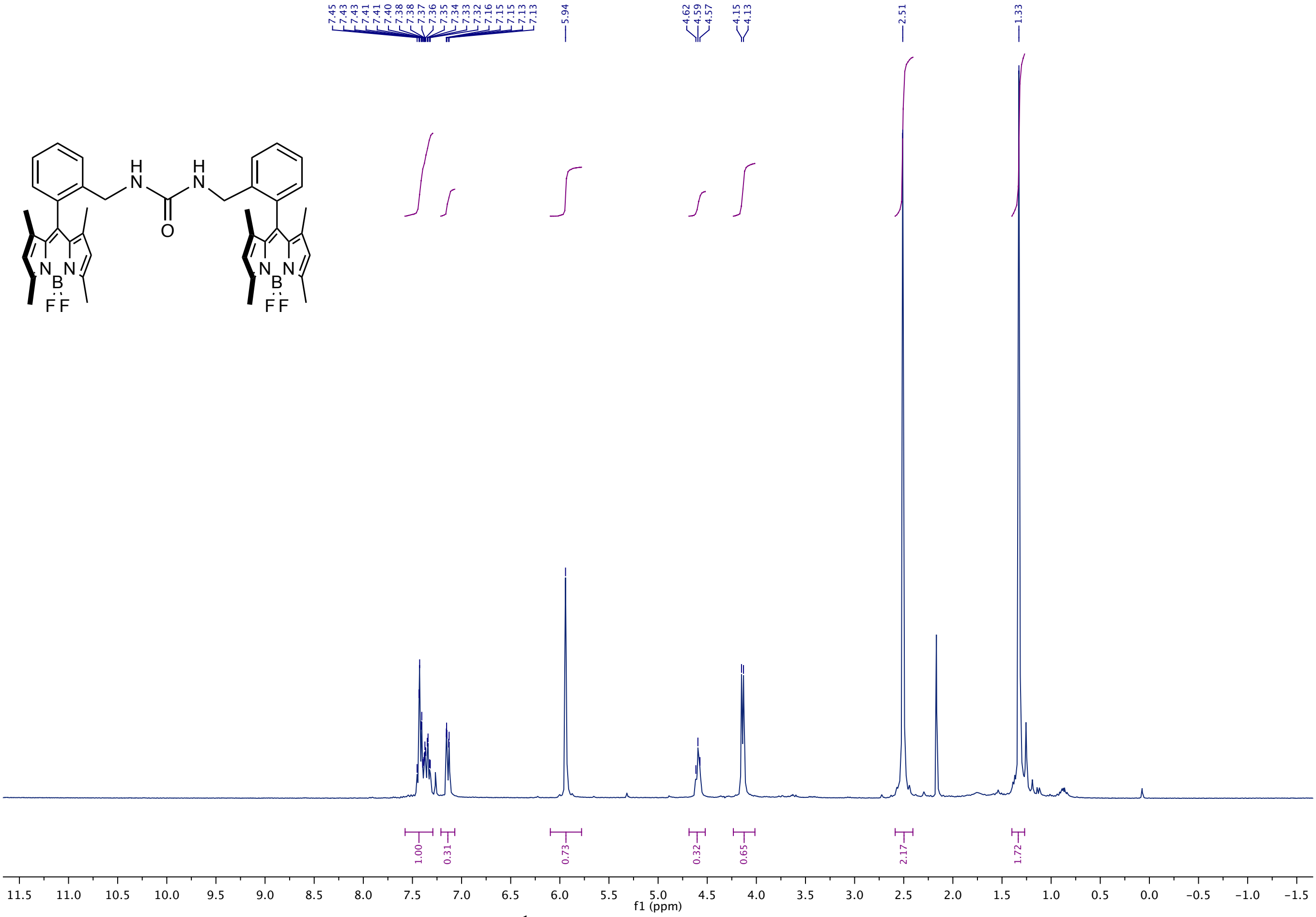

Figure S22. ^{1}H NMR spectrum of compound **4a**

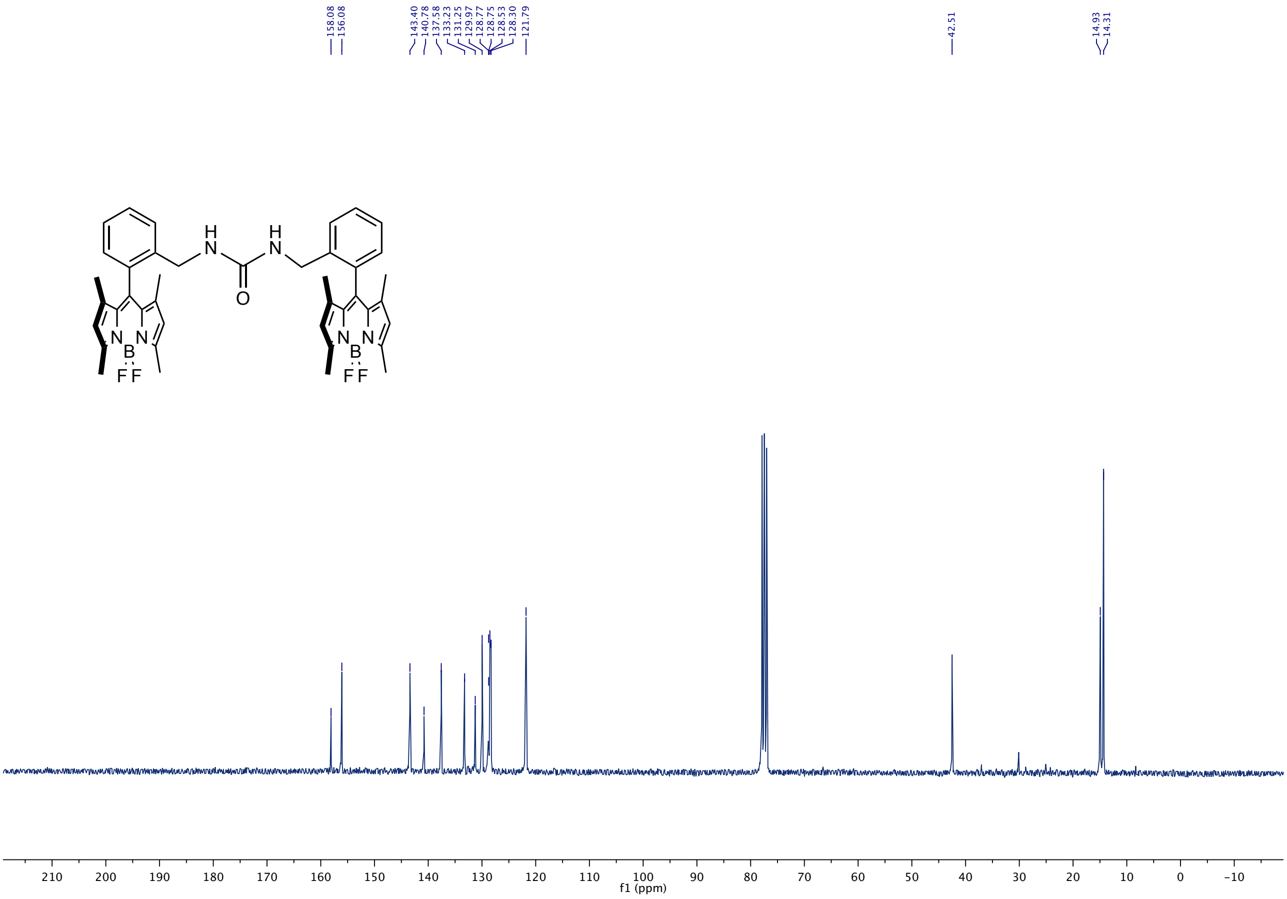

Figure S23. ^{13}C NMR spectrum of compound **4a**

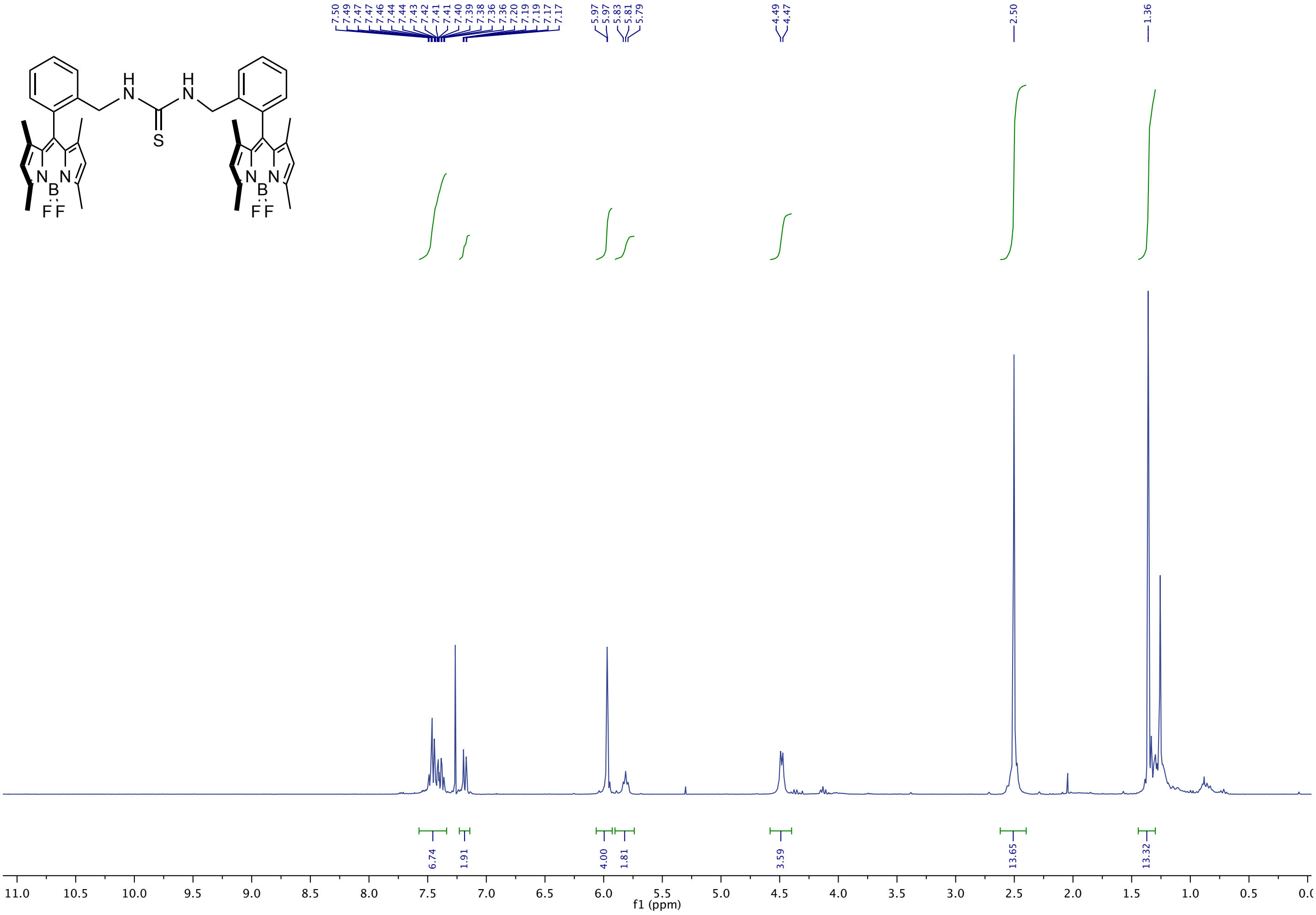

Figure S24. ^{1}H NMR spectrum of compound **4b**

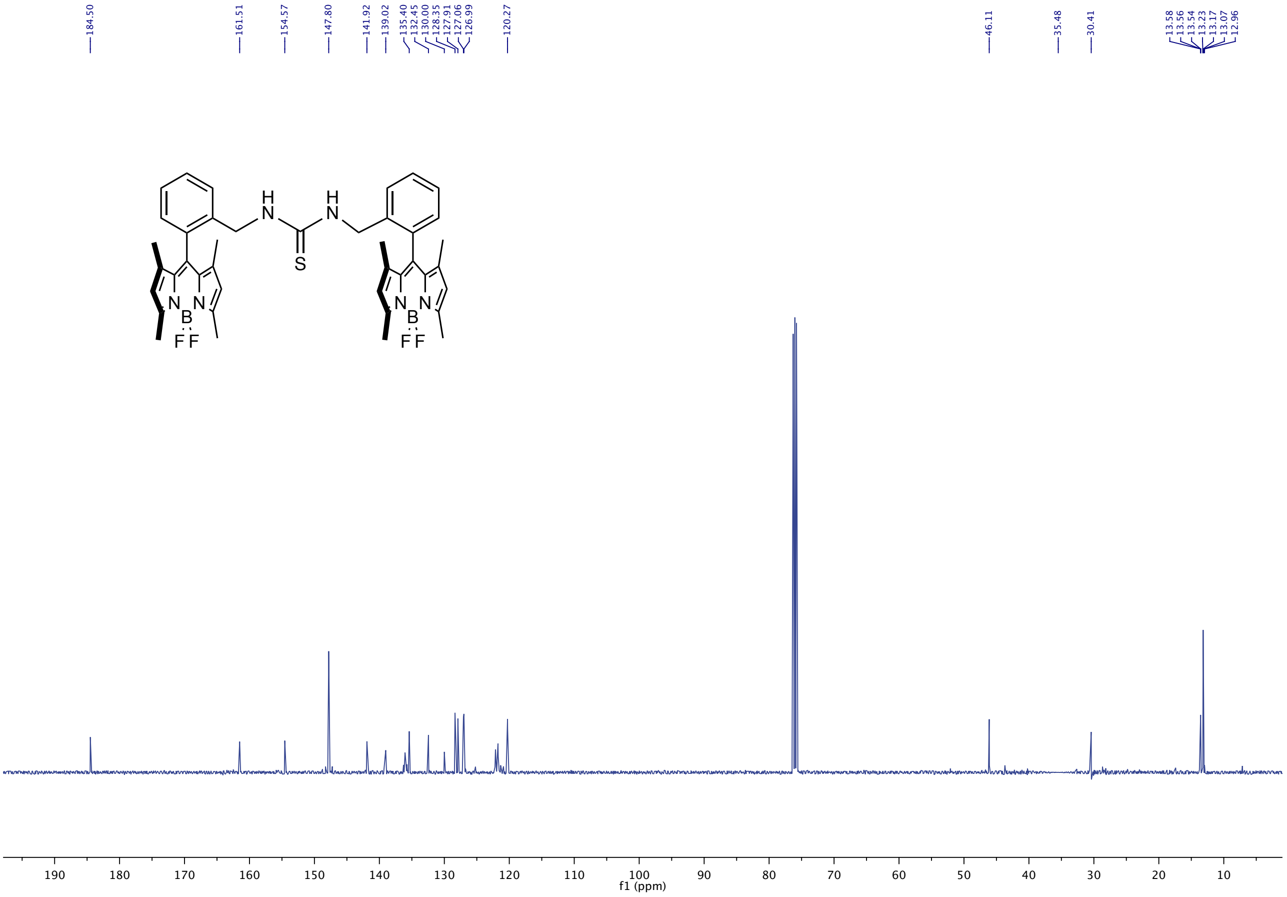

Figure S25. ^{13}C NMR spectrum of compound **4b**

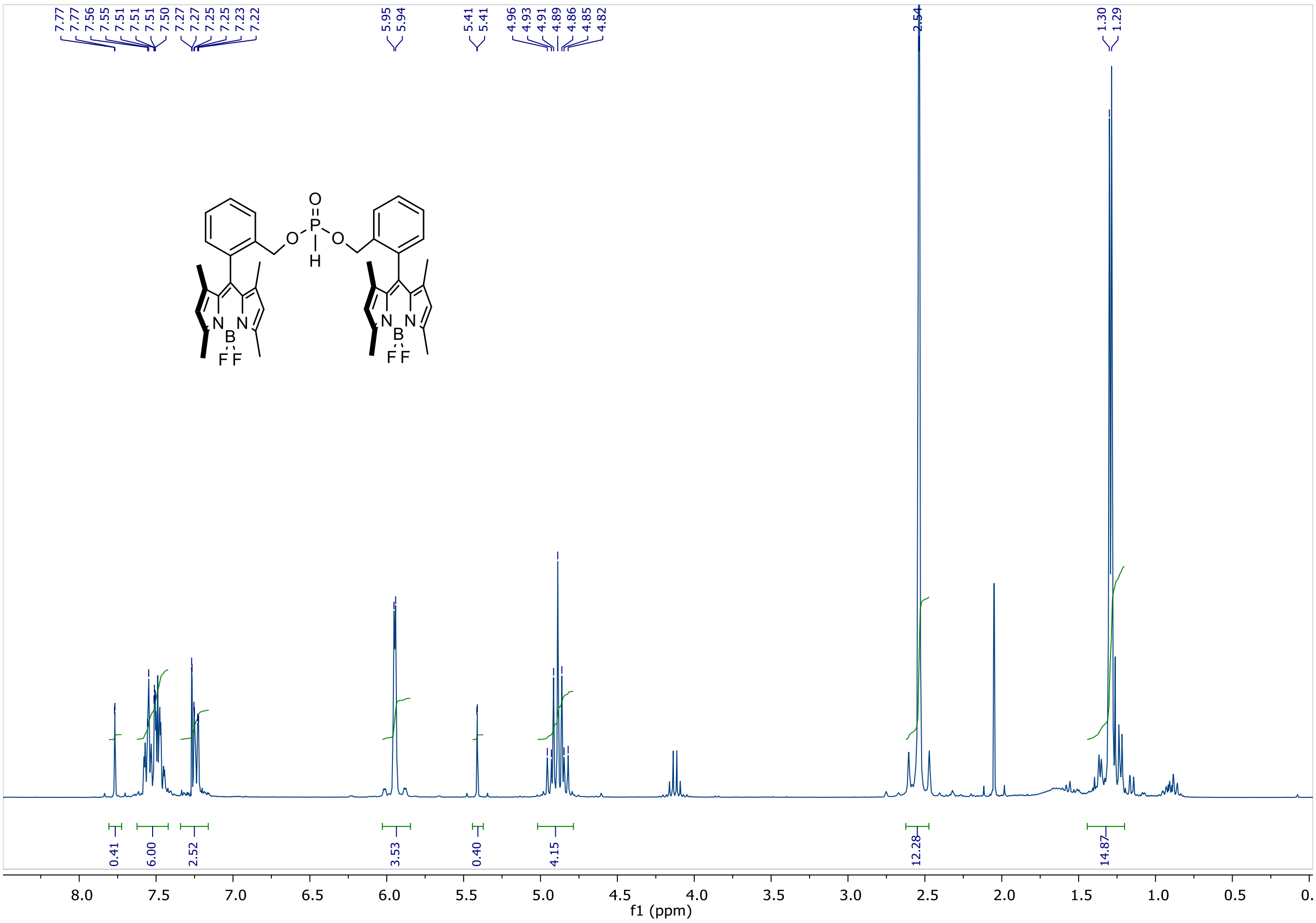

Figure S26. ^{1}H NMR spectrum of compound **4d**

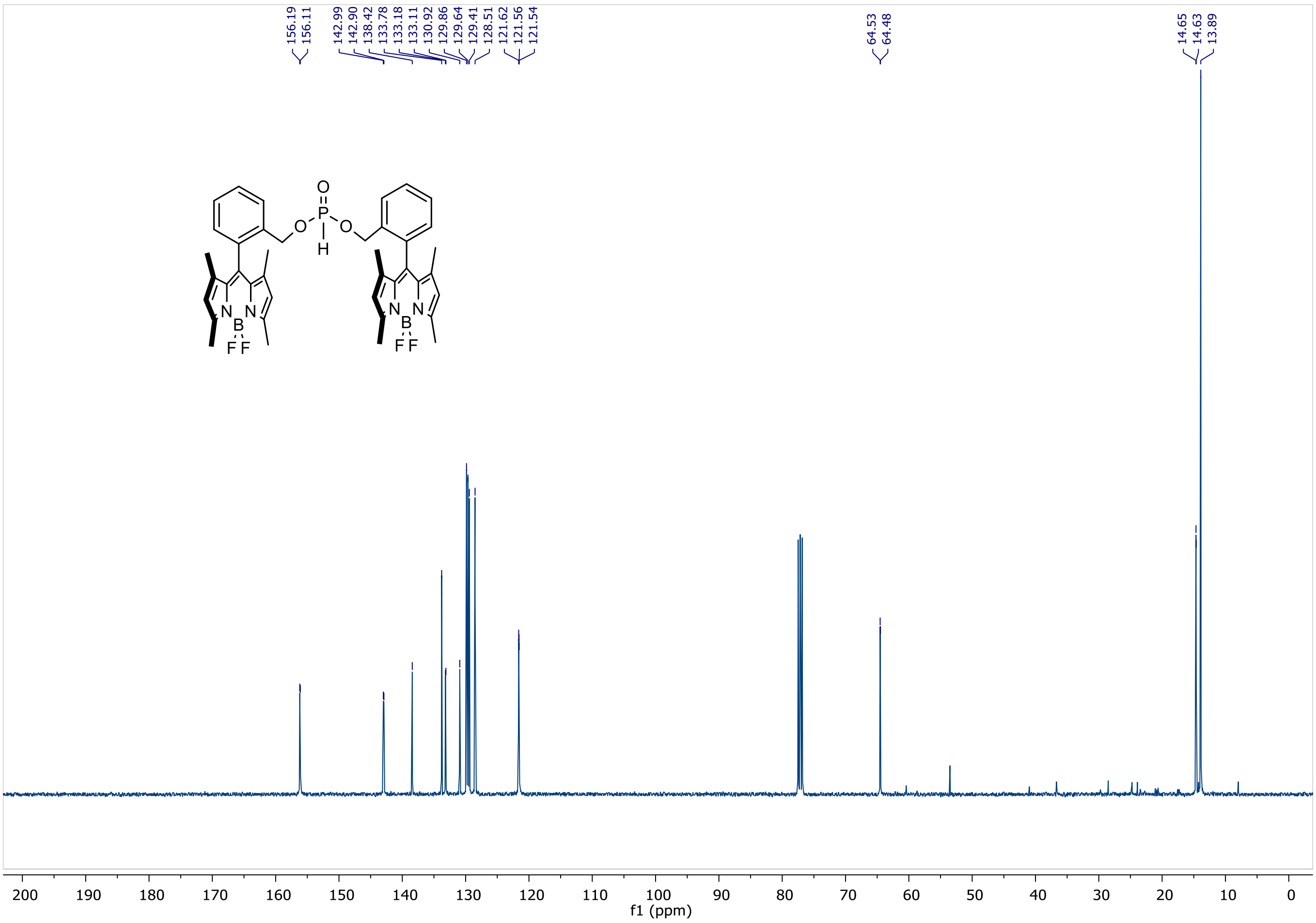

Figure S27. ^{13}C NMR spectrum of compound **4d**

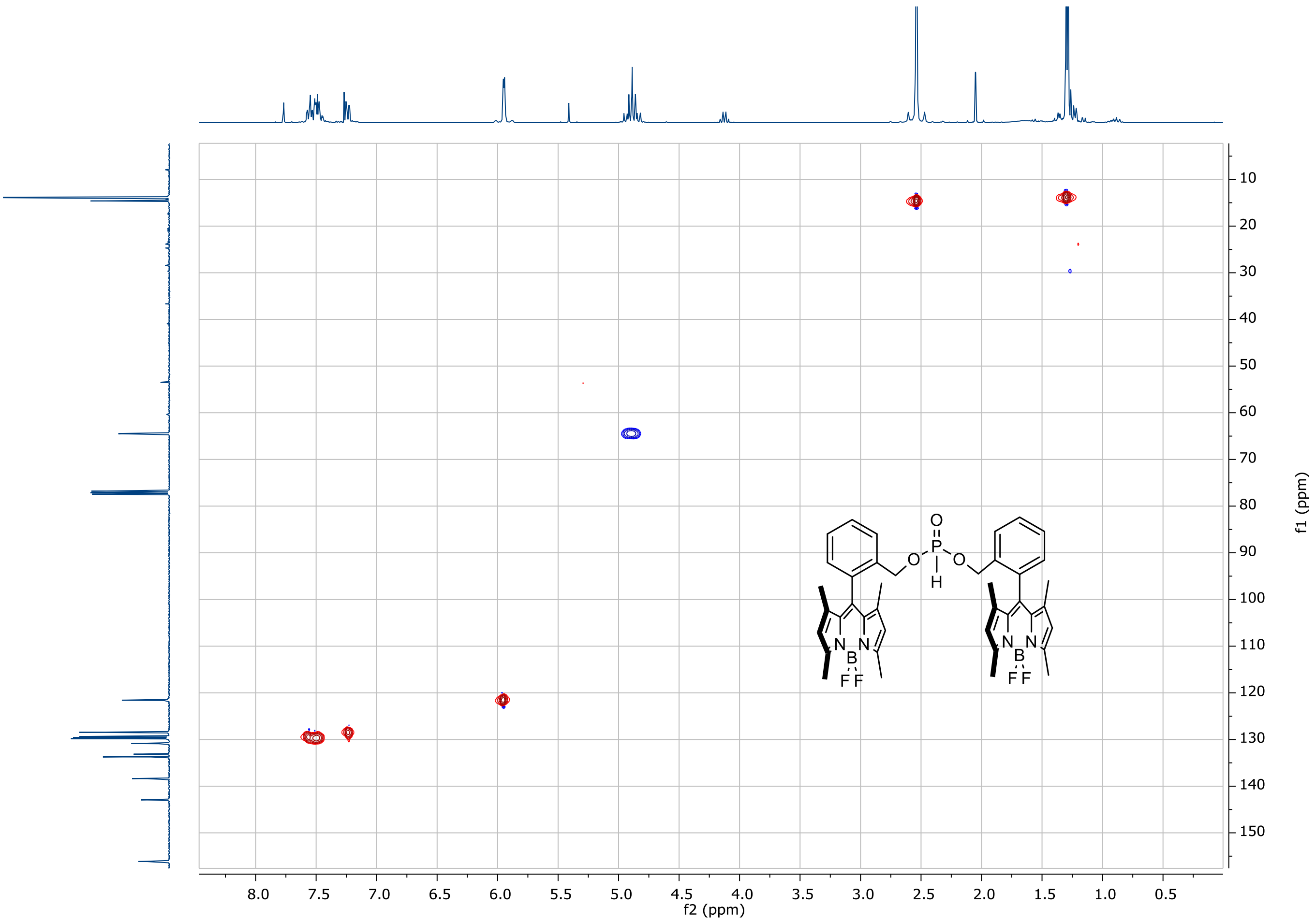

Figure S28. HSQC NMR spectrum of compound **4d**

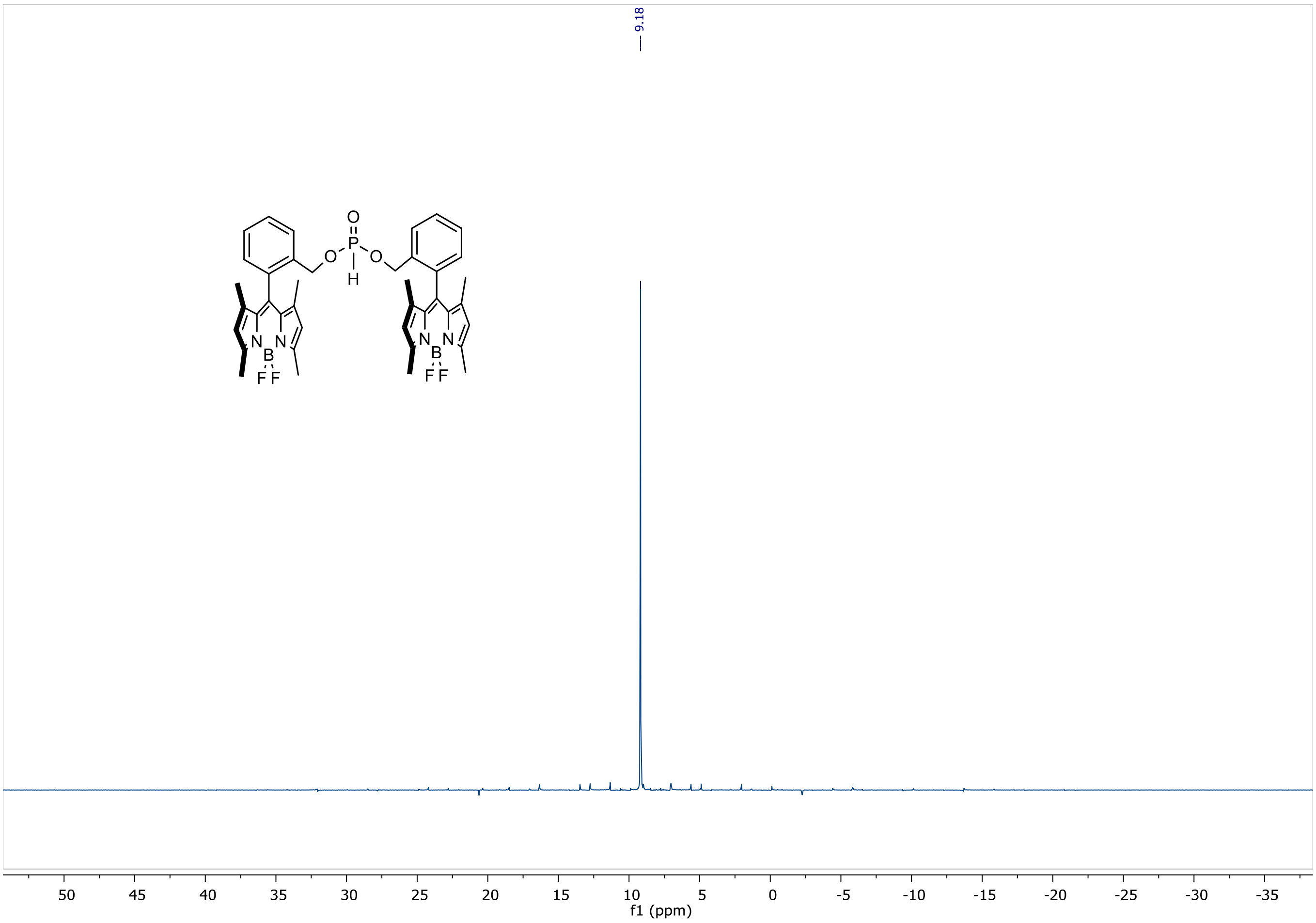

Figure S29. ^{31}P-NMR spectrum of compound **4d**

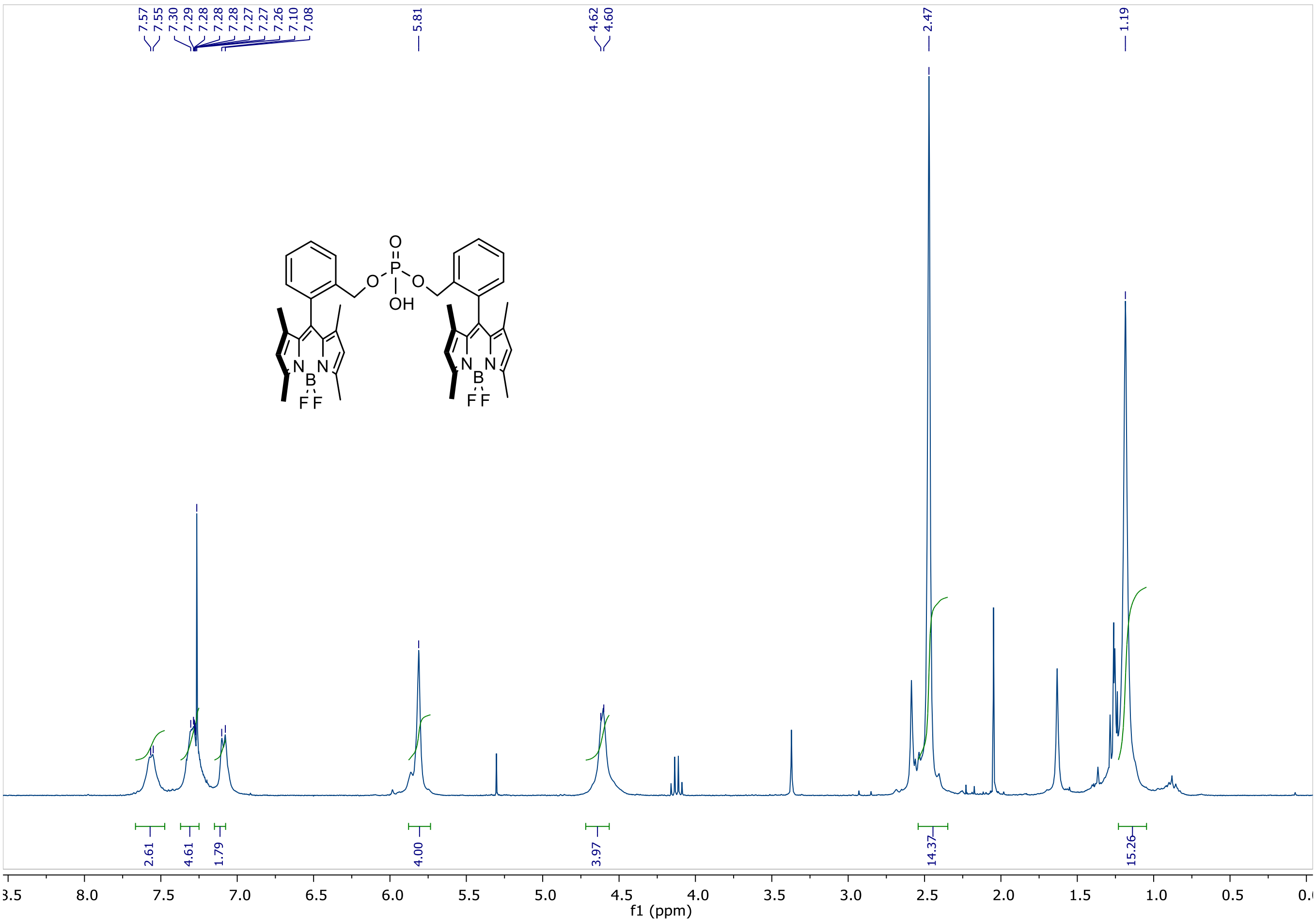

Figure S30. ^{1}H NMR spectrum of compound **4e**

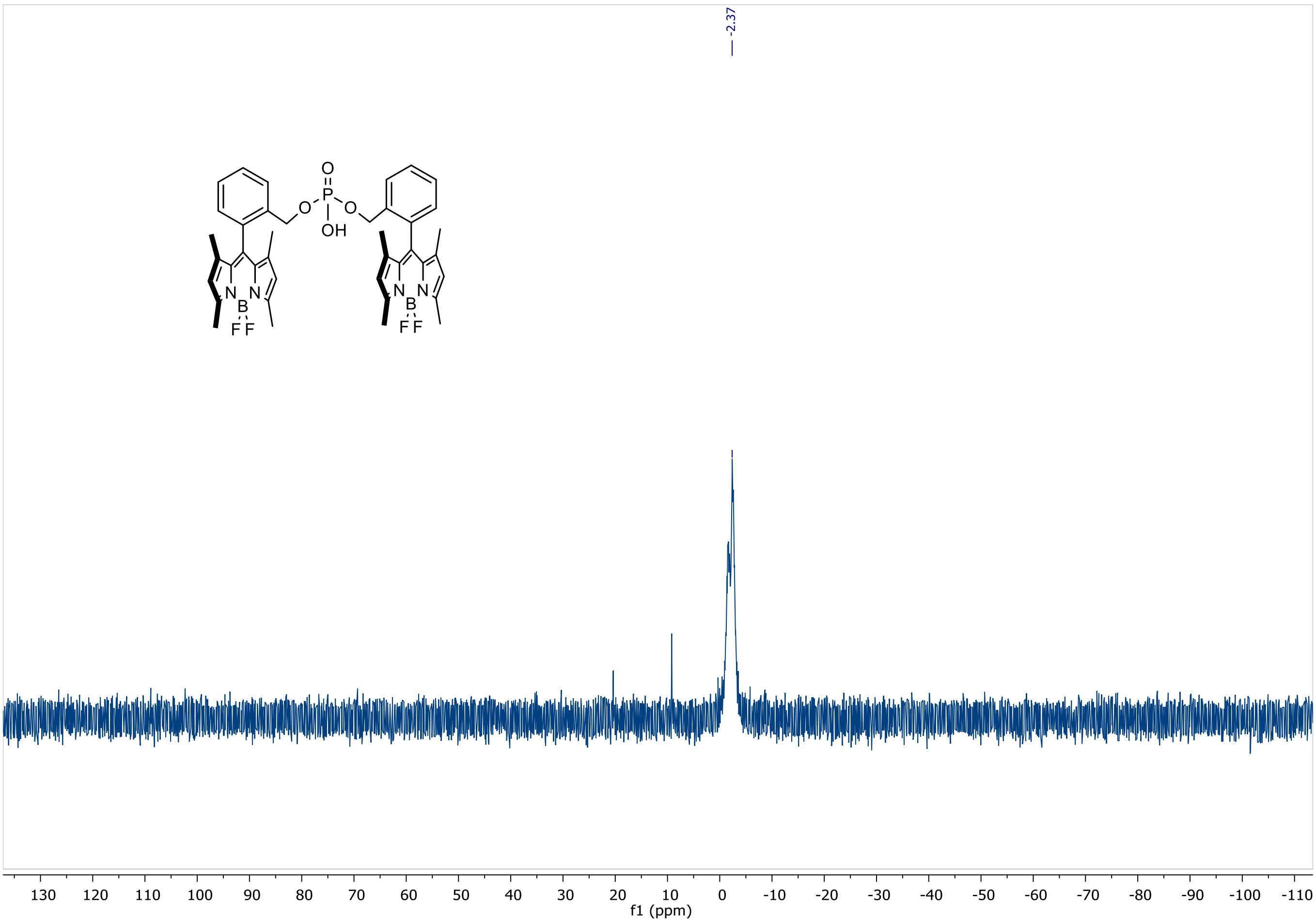

Figure S31. ^{31}P-NMR spectrum of compound **4e**

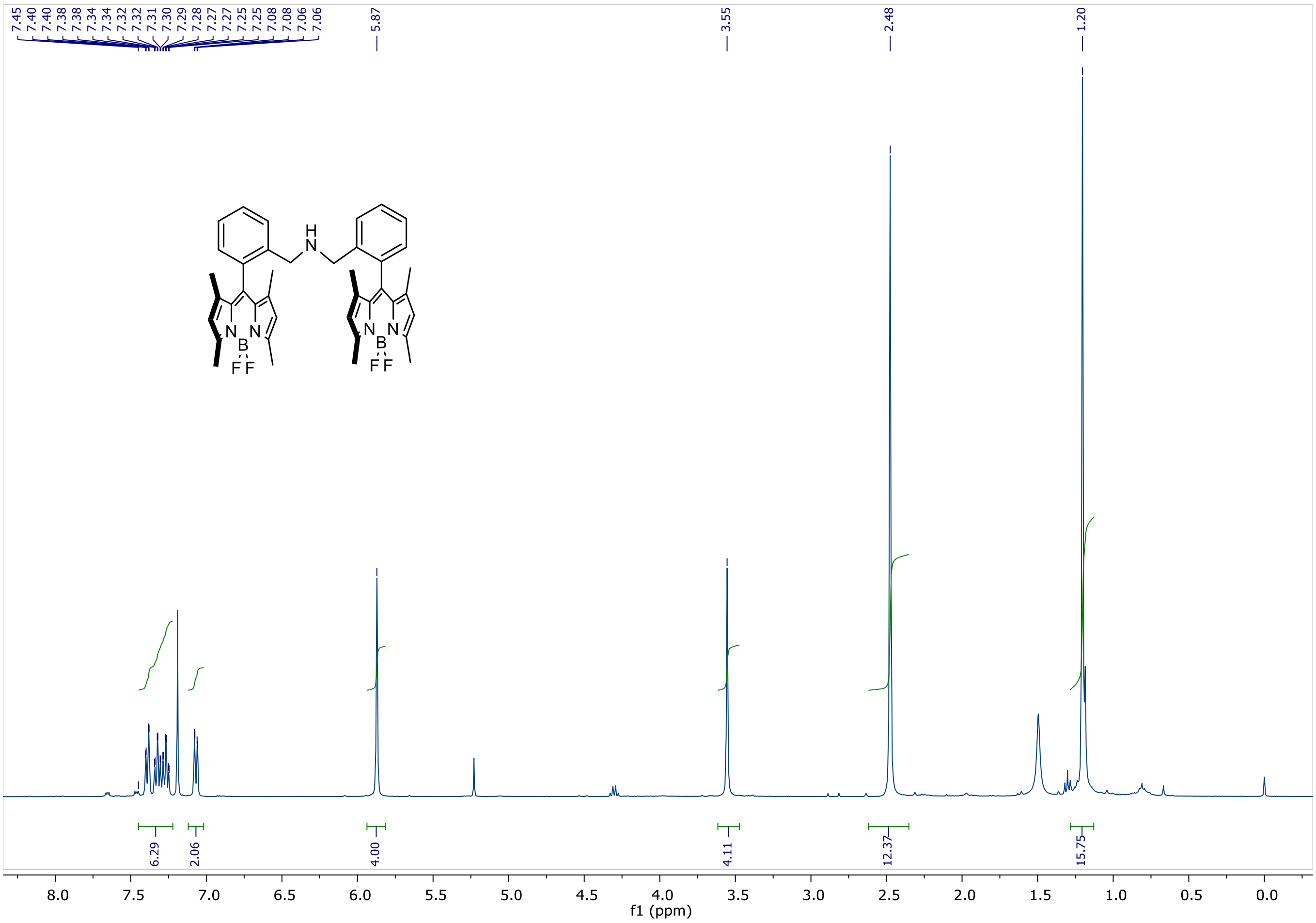

Figure S32. ^{1}H NMR spectrum of compound **4f**

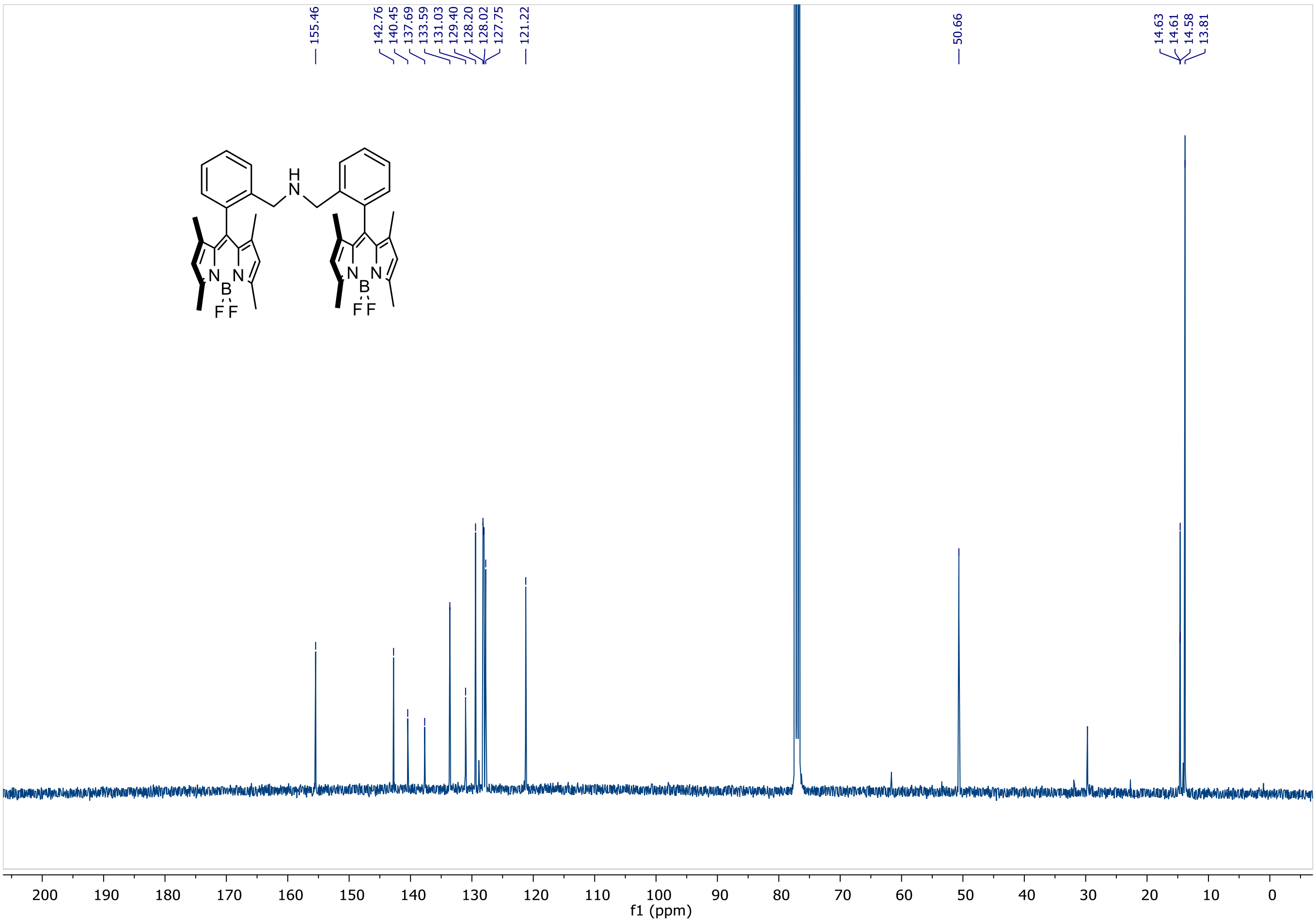

Figure S33. ^{13}C NMR spectrum of compound **4f**

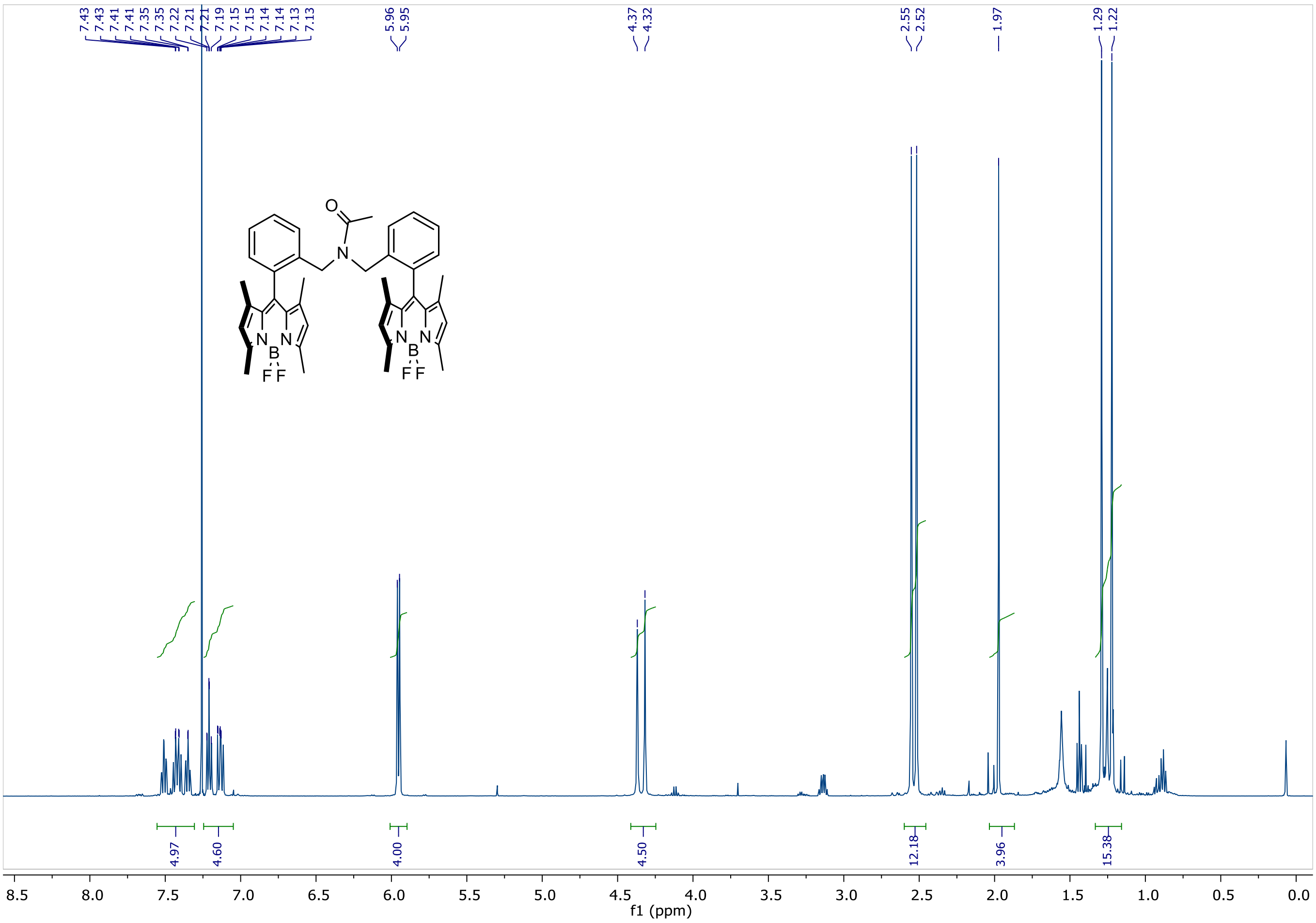

Figure S34. ^{1}H NMR spectrum of compound **4g**

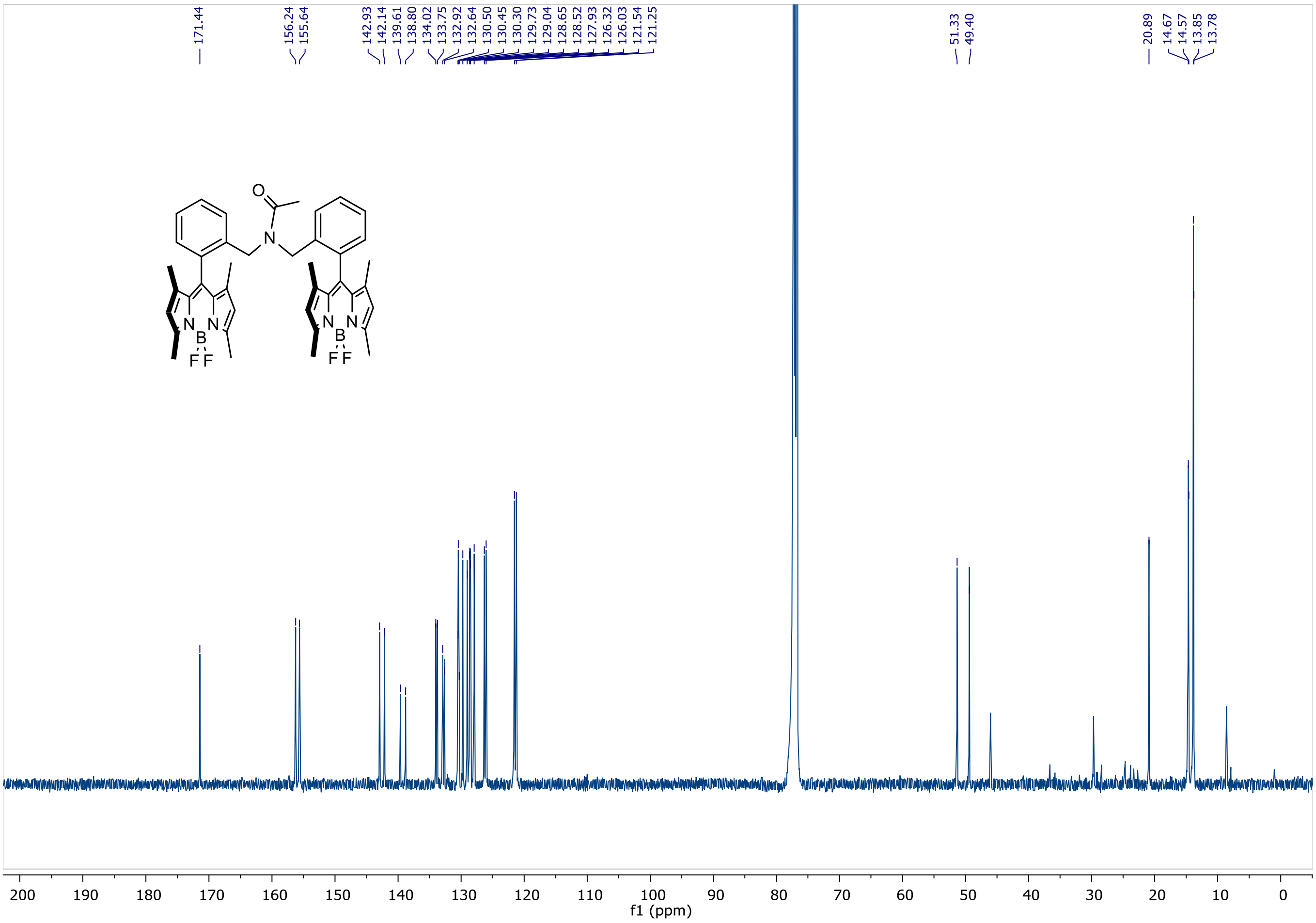

Figure S35. ^{13}C NMR spectrum of compound **4g**

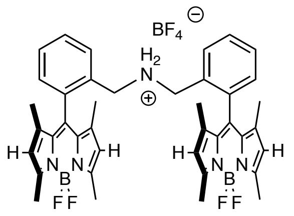

4h

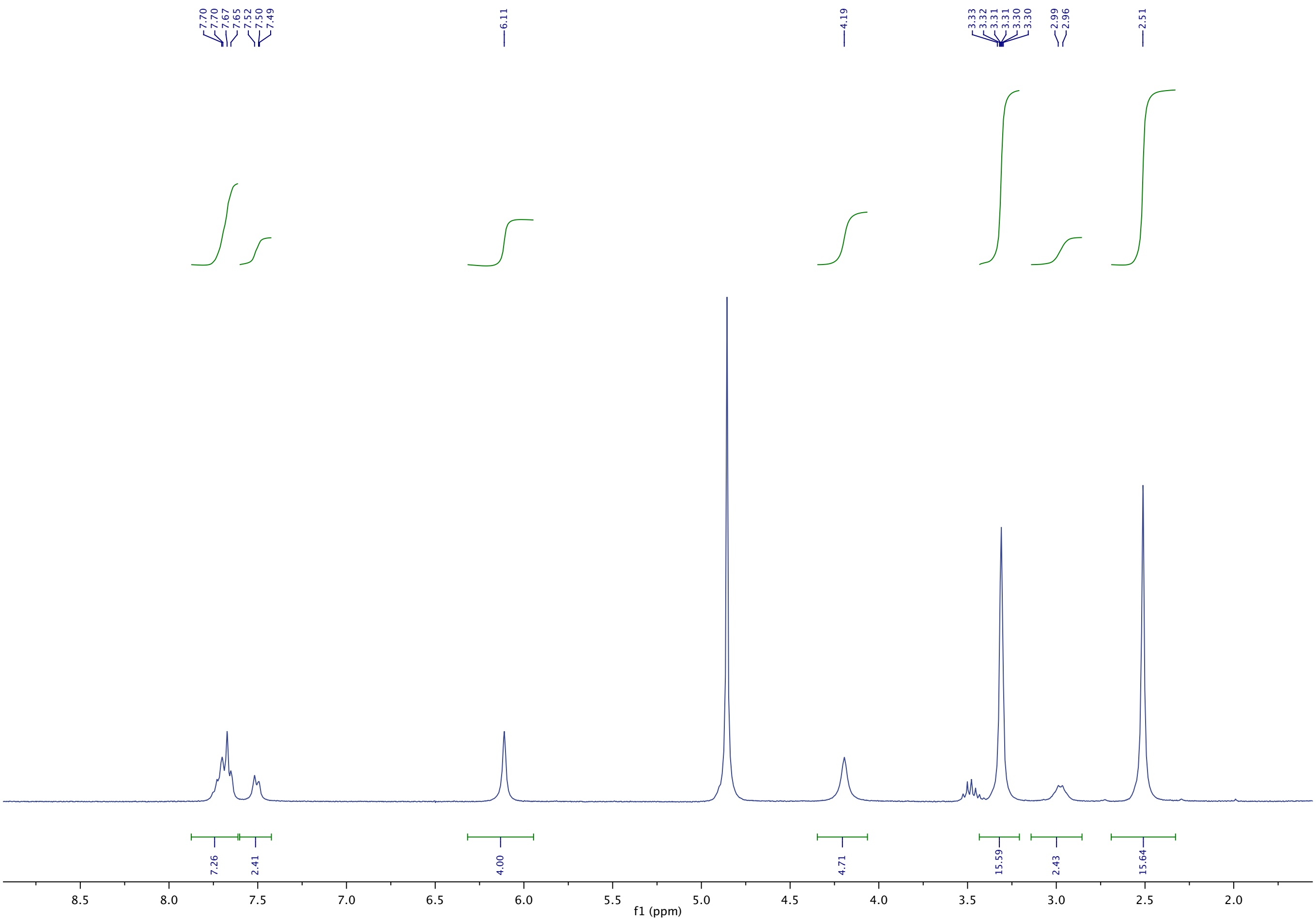

Figure S36. ¹H NMR spectrum of compound **4h**

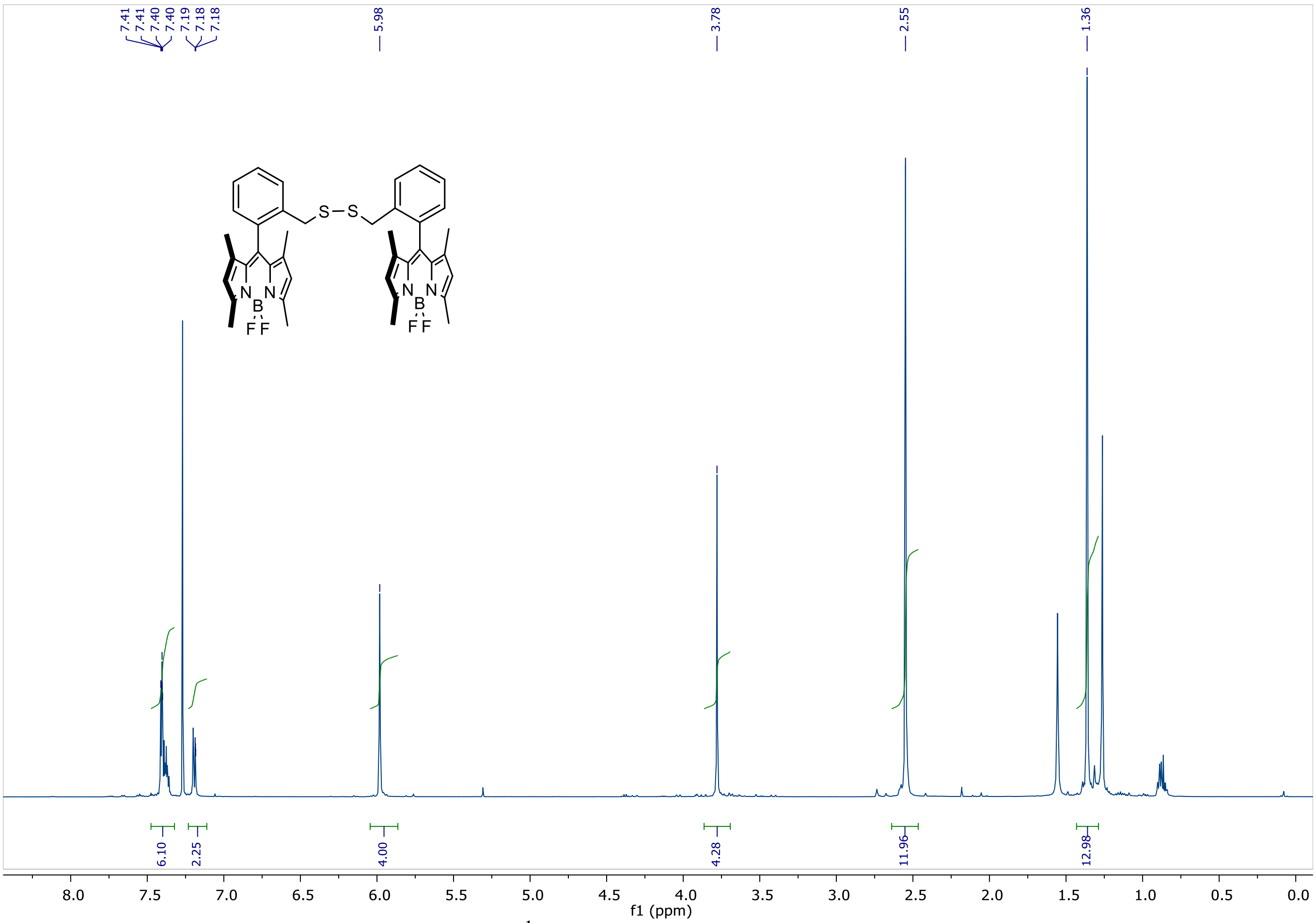

Figure S37. ^{1}H NMR spectrum of compound **4i**

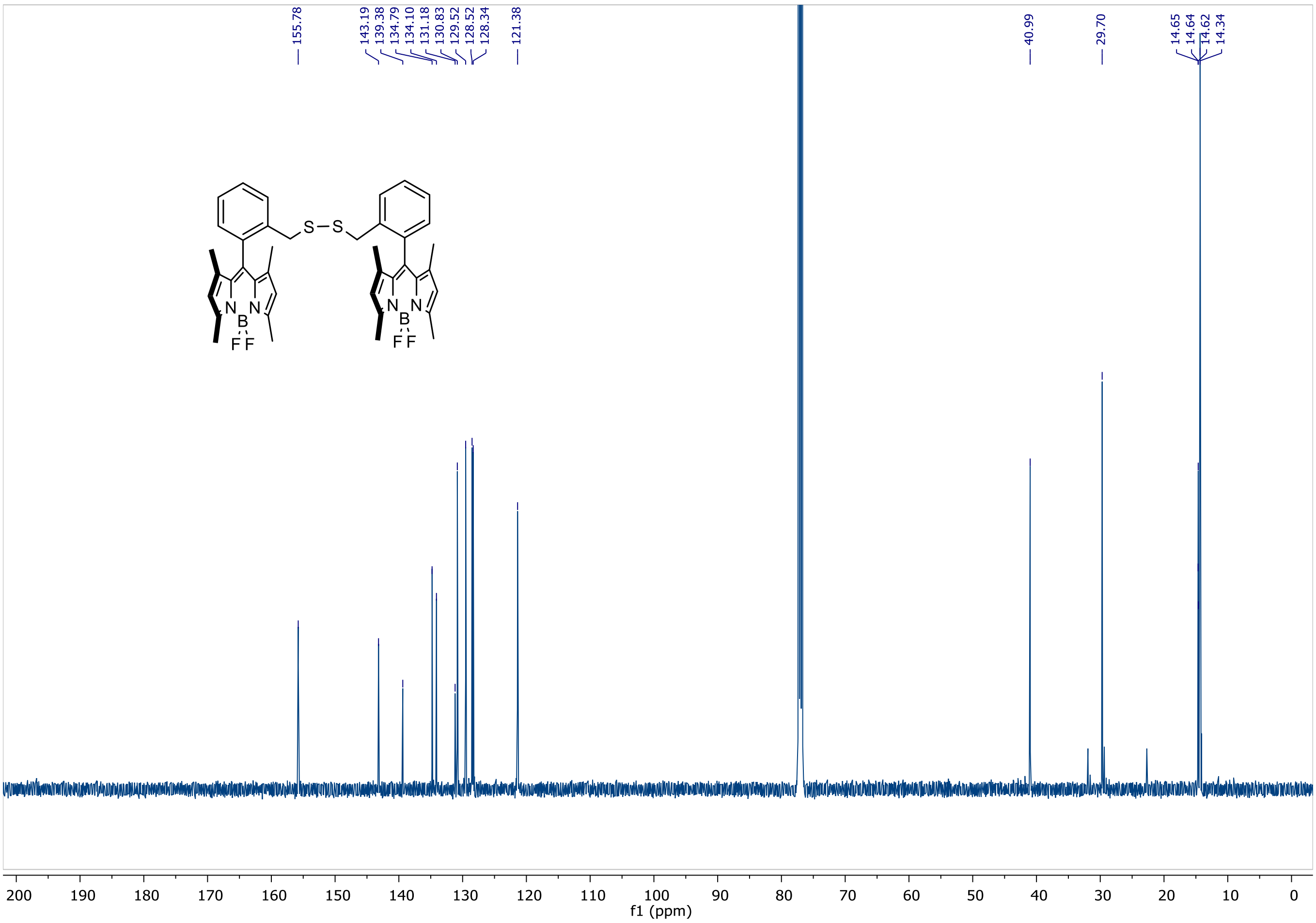

Figure S38. ^{13}C NMR spectrum of compound **4i**

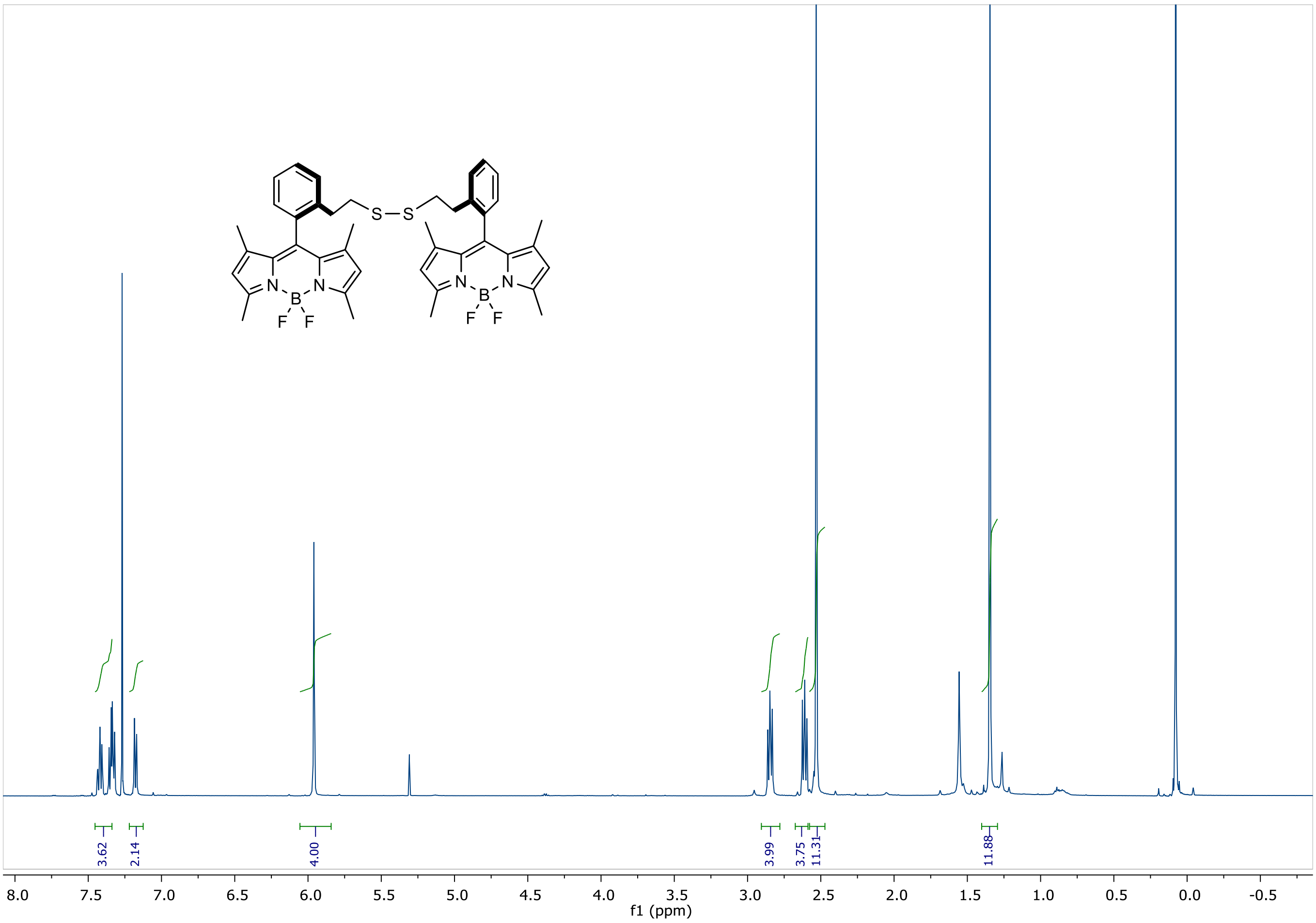

Figure S39. ^{1}H NMR spectrum of compound **4j**

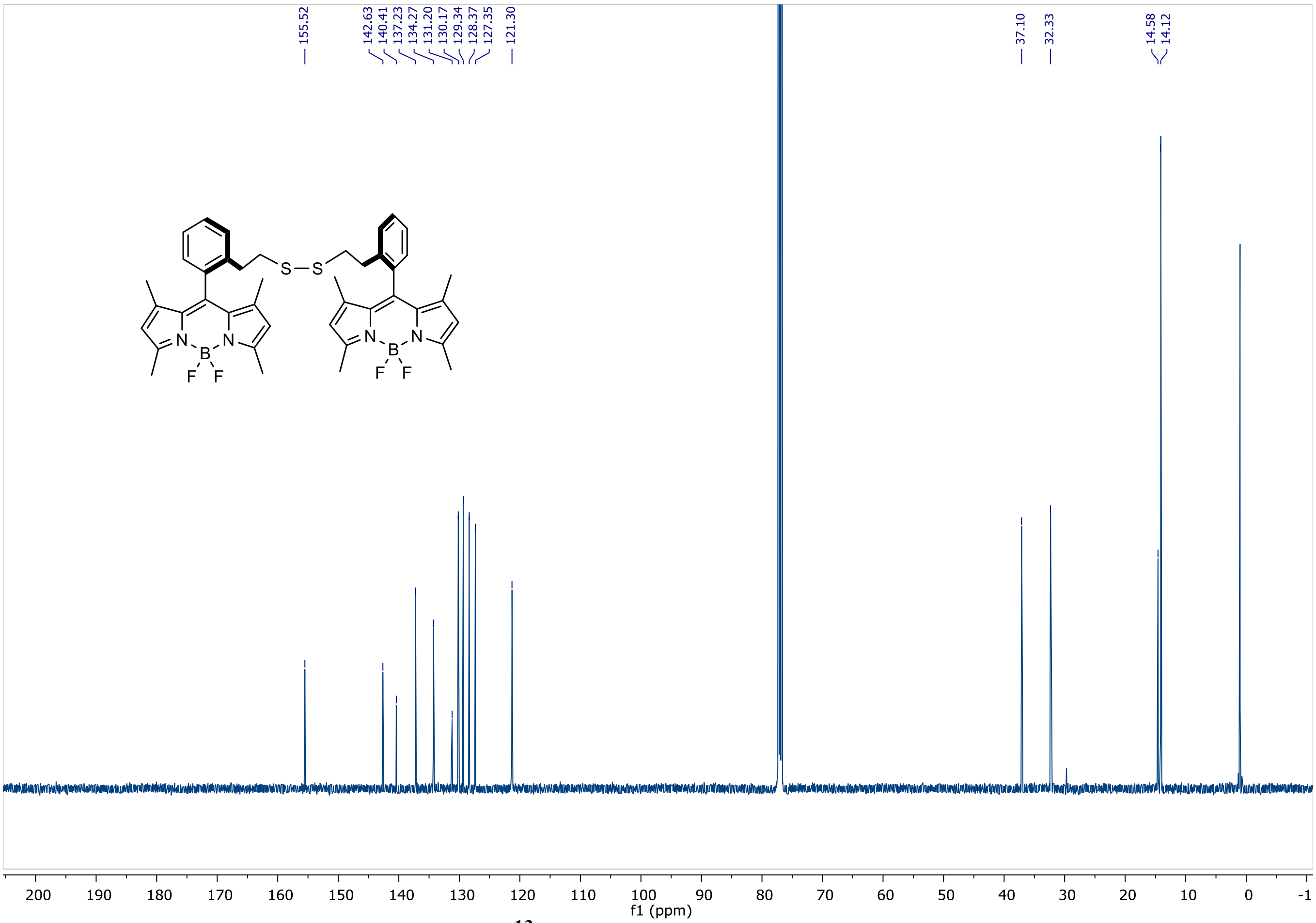

Figure S40. ^{13}C NMR spectrum of **compound 4j**

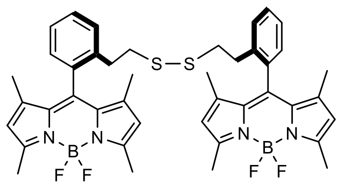

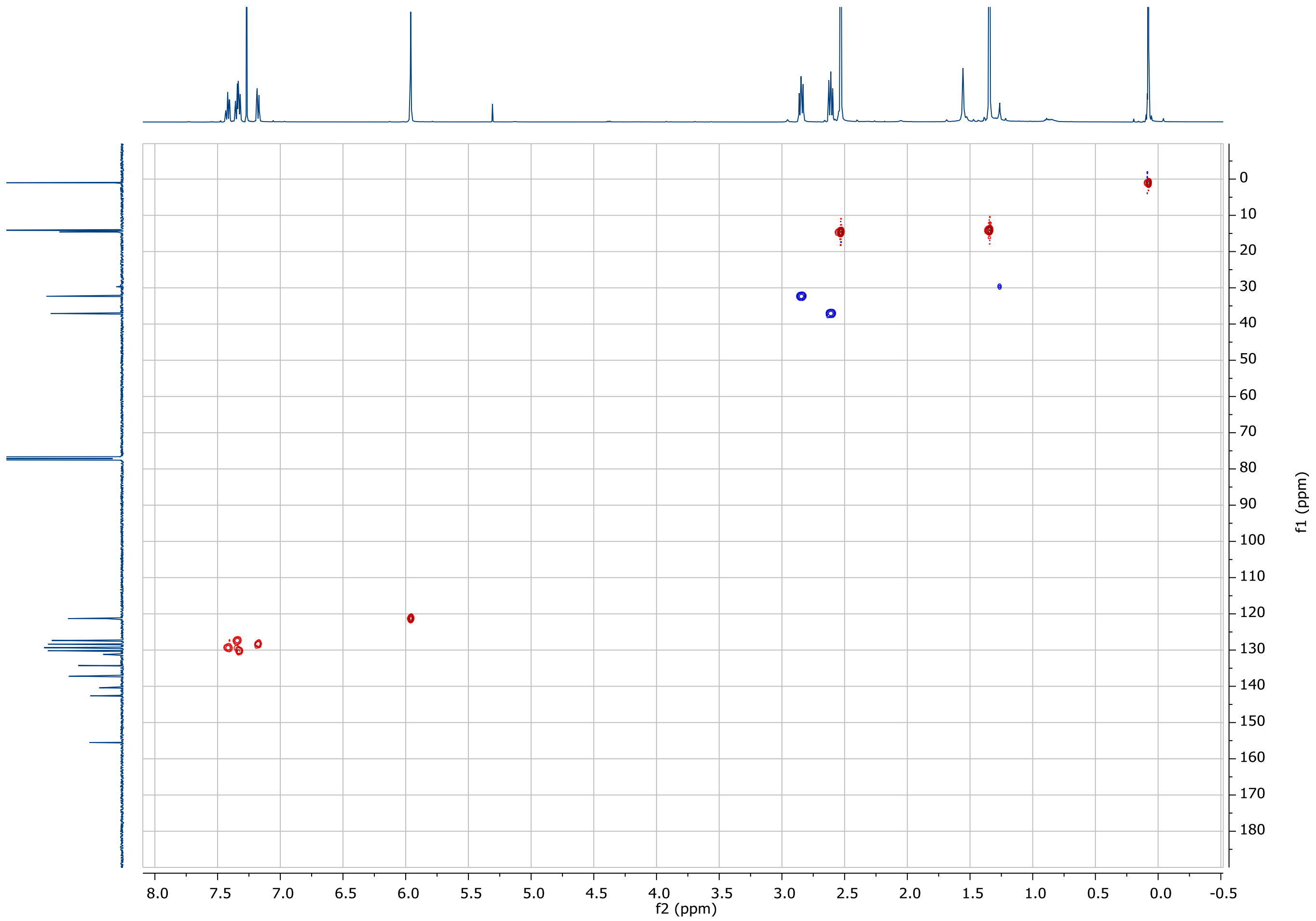

Figure S41. HSQC NMR spectrum of compound **4j**

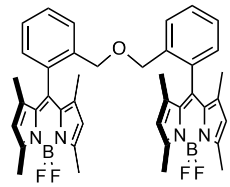

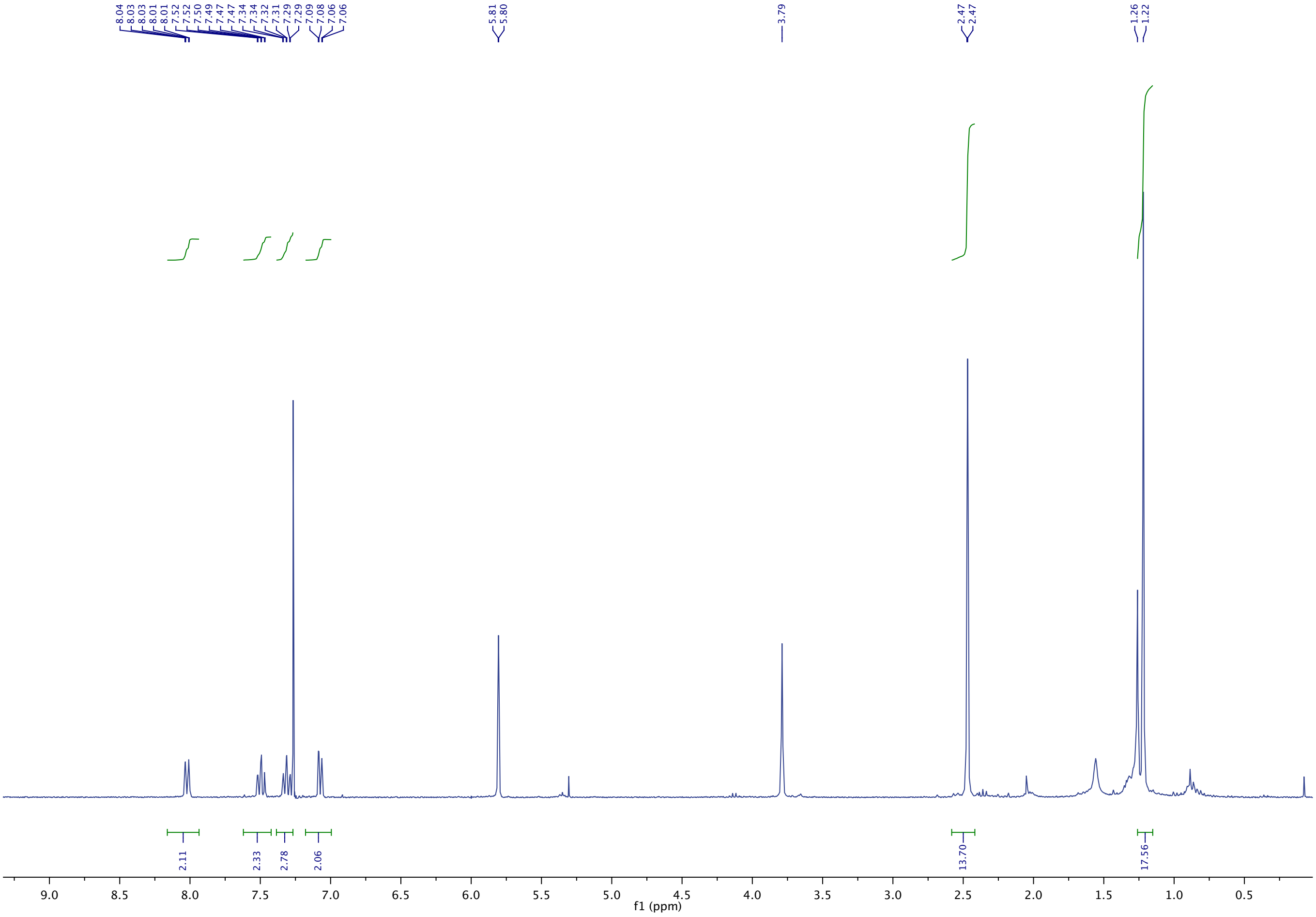

Figure S42. ^{1}H NMR spectrum of compound **4k**

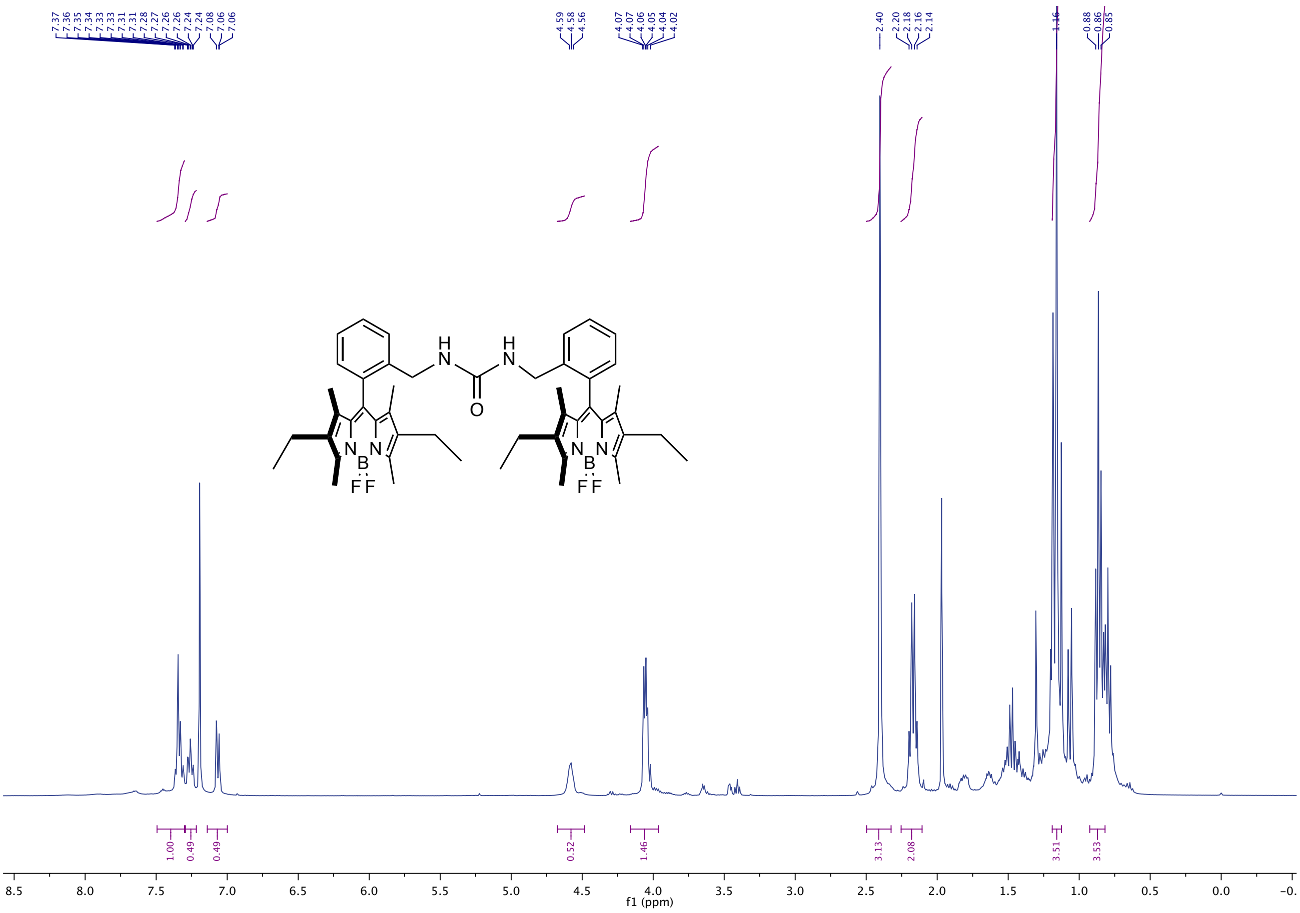

Figure S43. ^{1}H NMR spectrum of compound **5a**

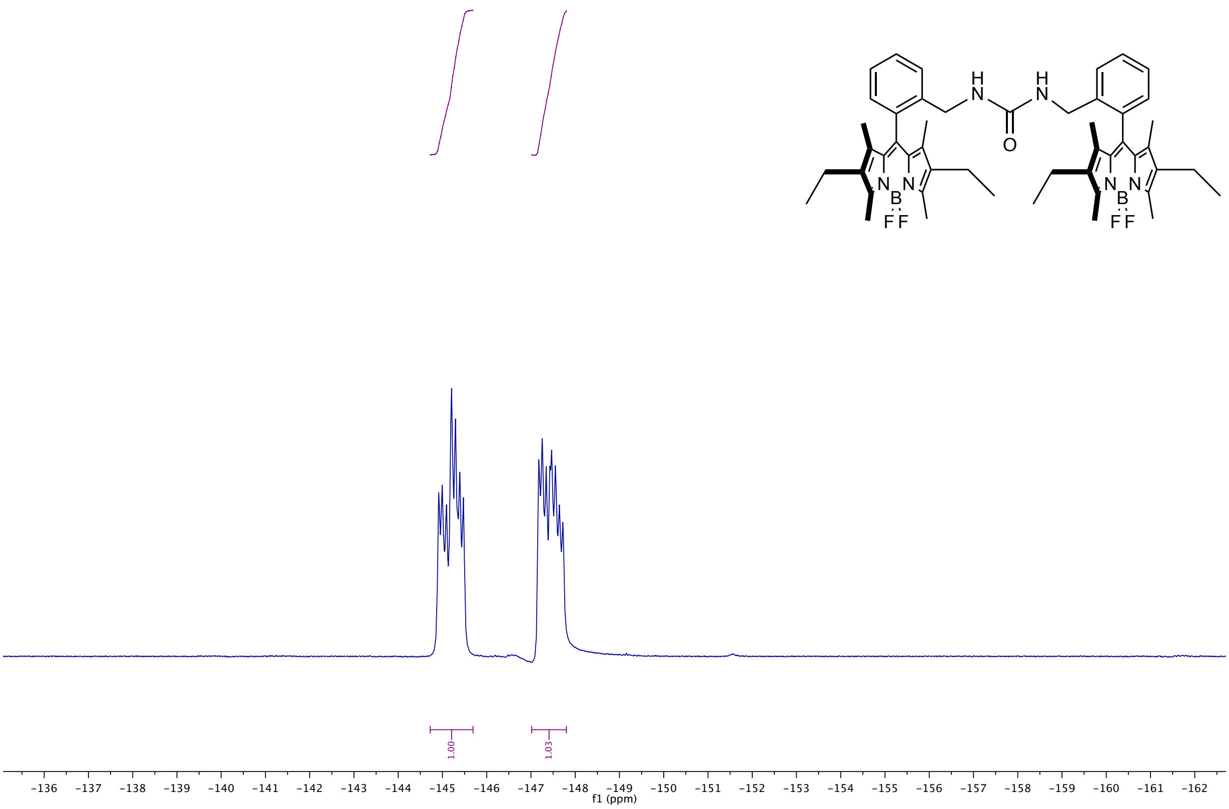

Figure S44. ^{19}F NMR spectrum of compound **5a**

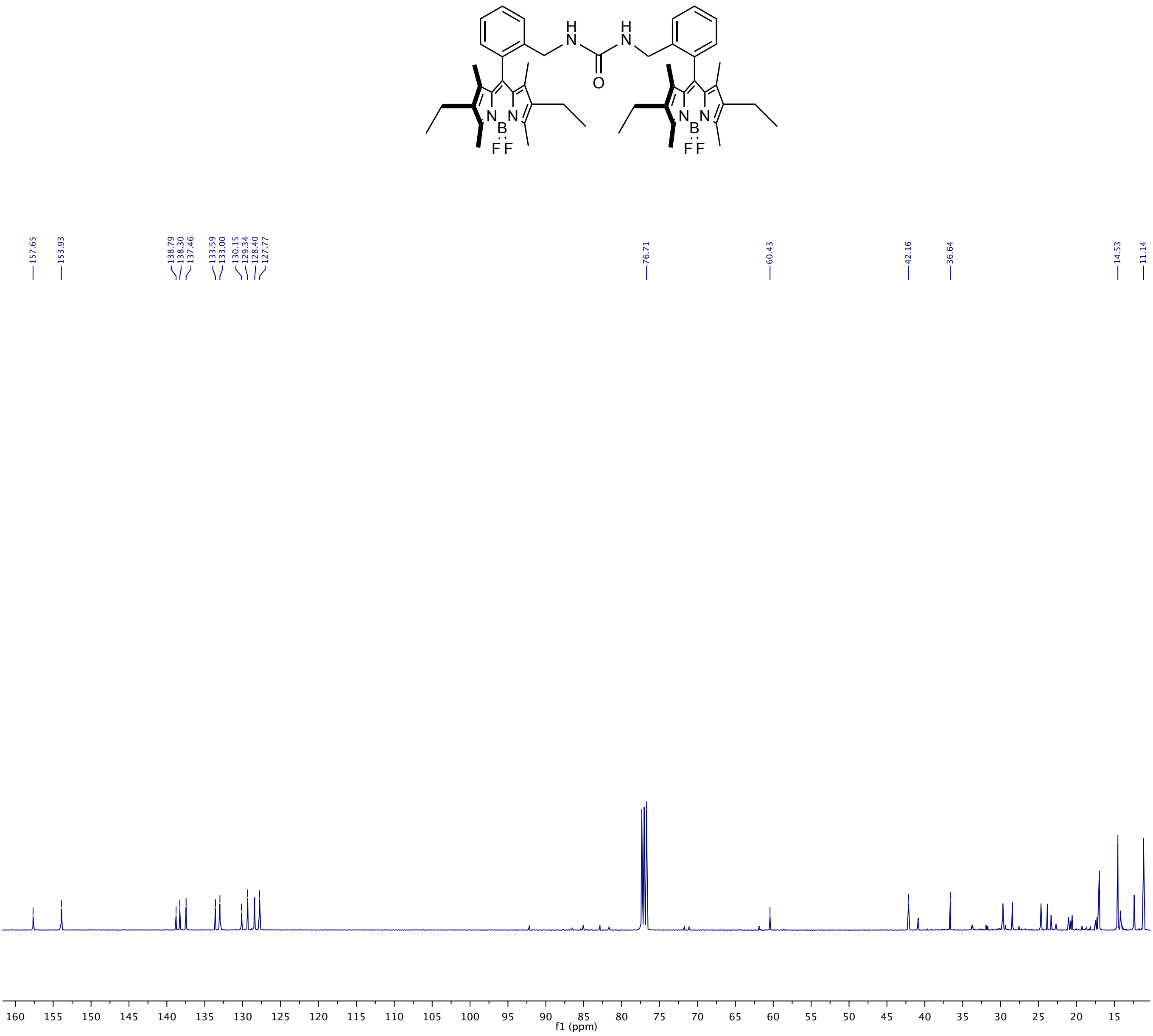

Figure S45. ^{13}C NMR spectrum of compound **5a**

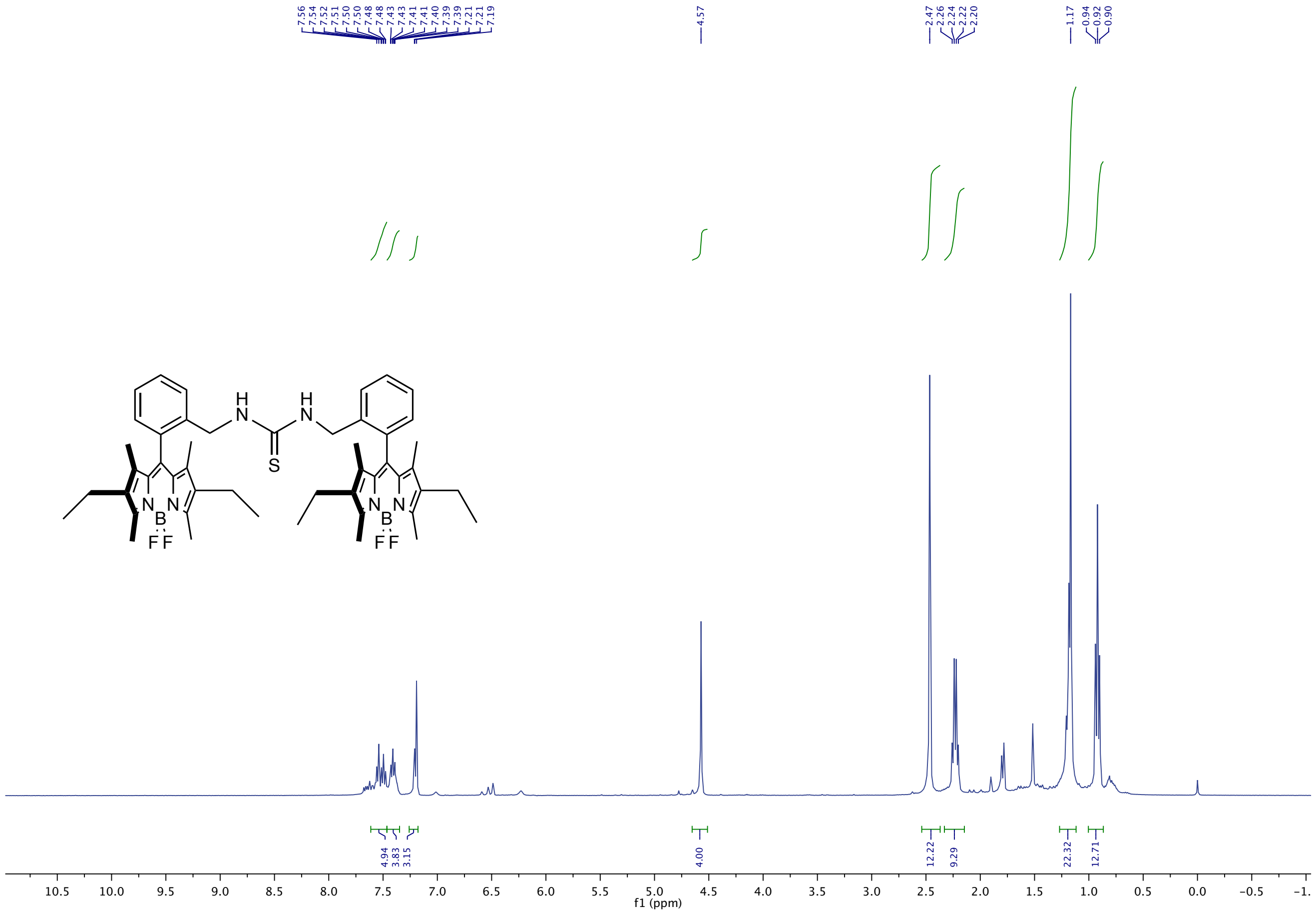

Figure S46 ^{1}H NMR spectrum of compound **5b**

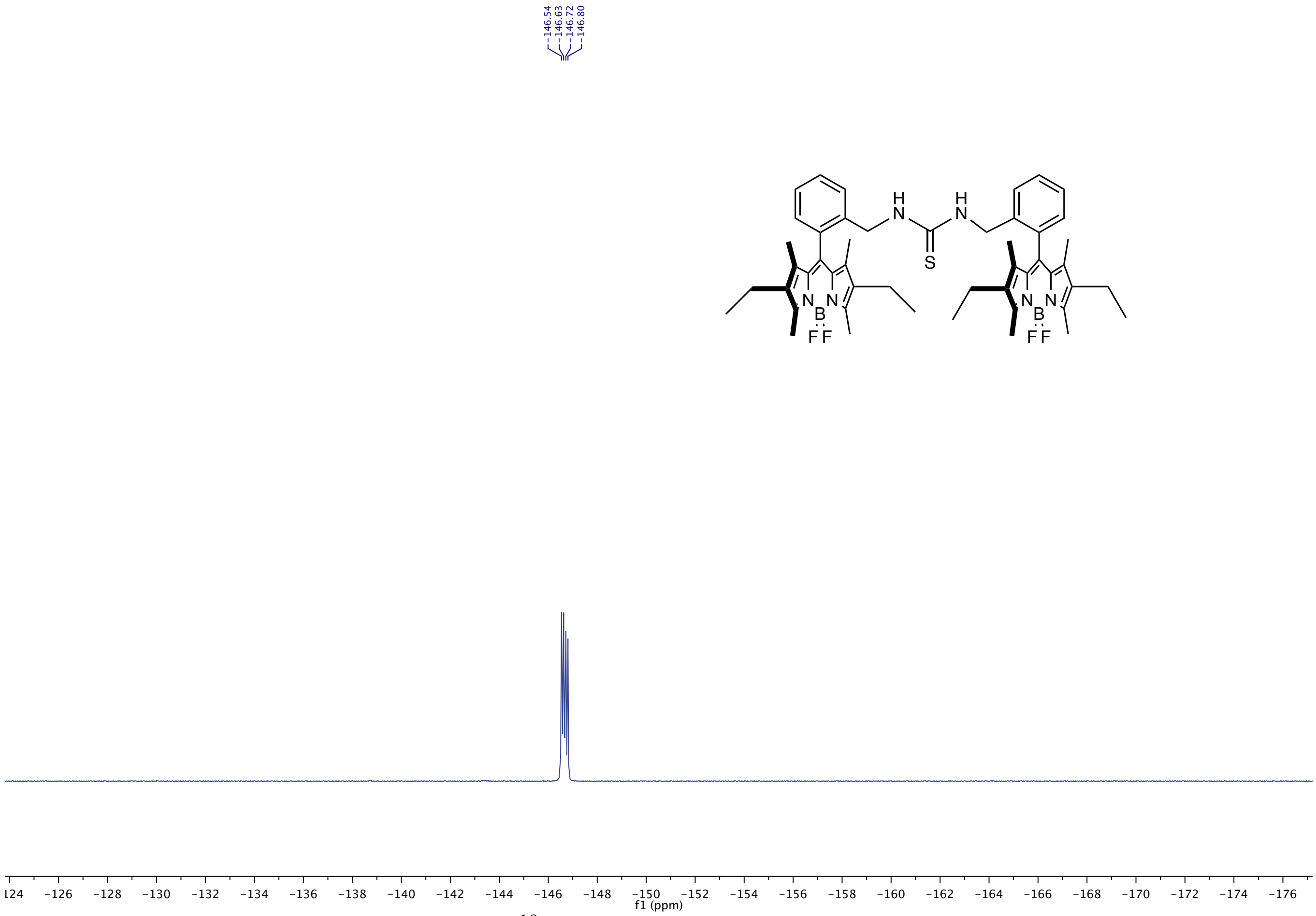

Figure S47. ^{19}F NMR spectrum of compound **5b**

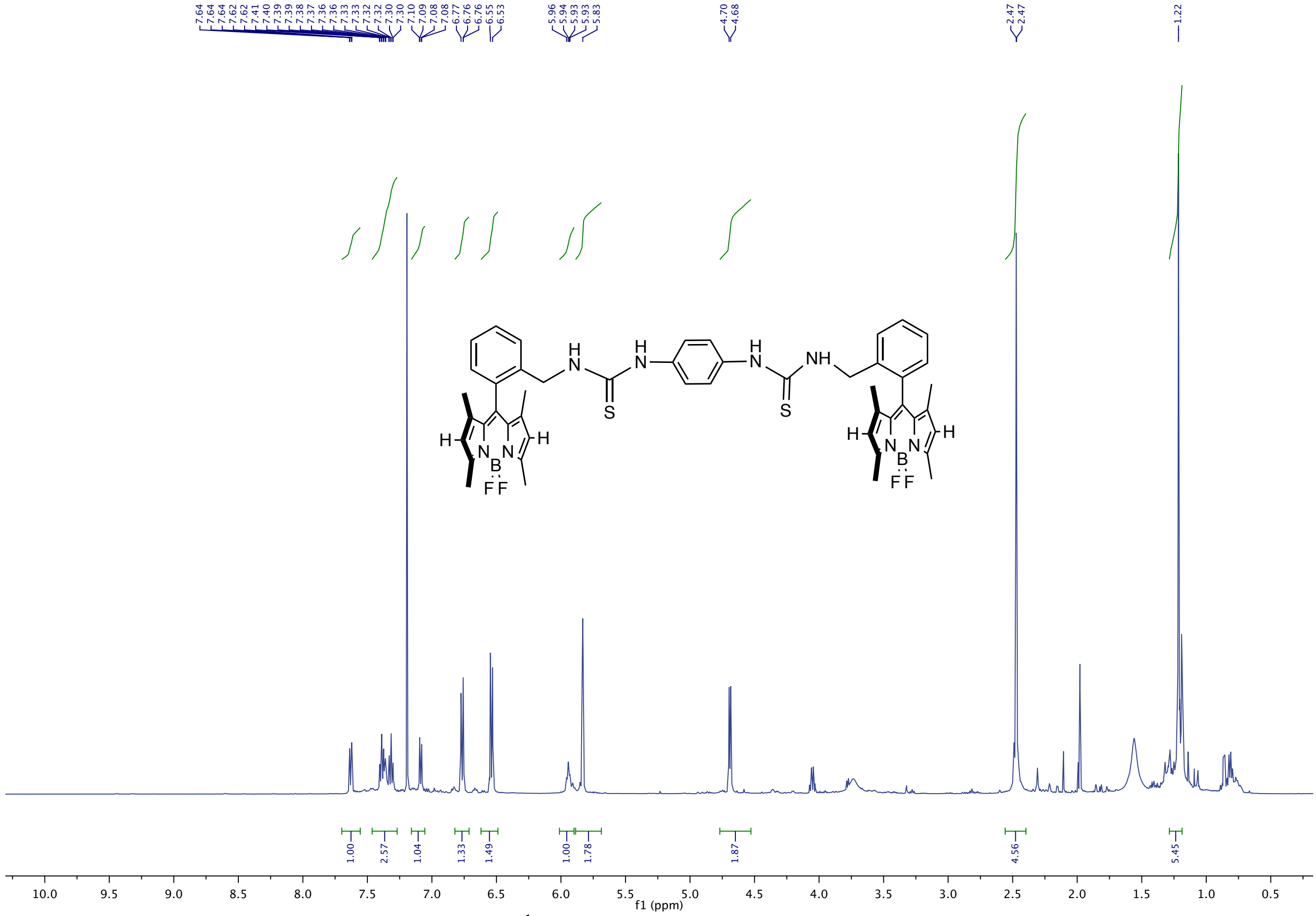

Figure S48. ^{1}H NMR spectrum of compound **5c**

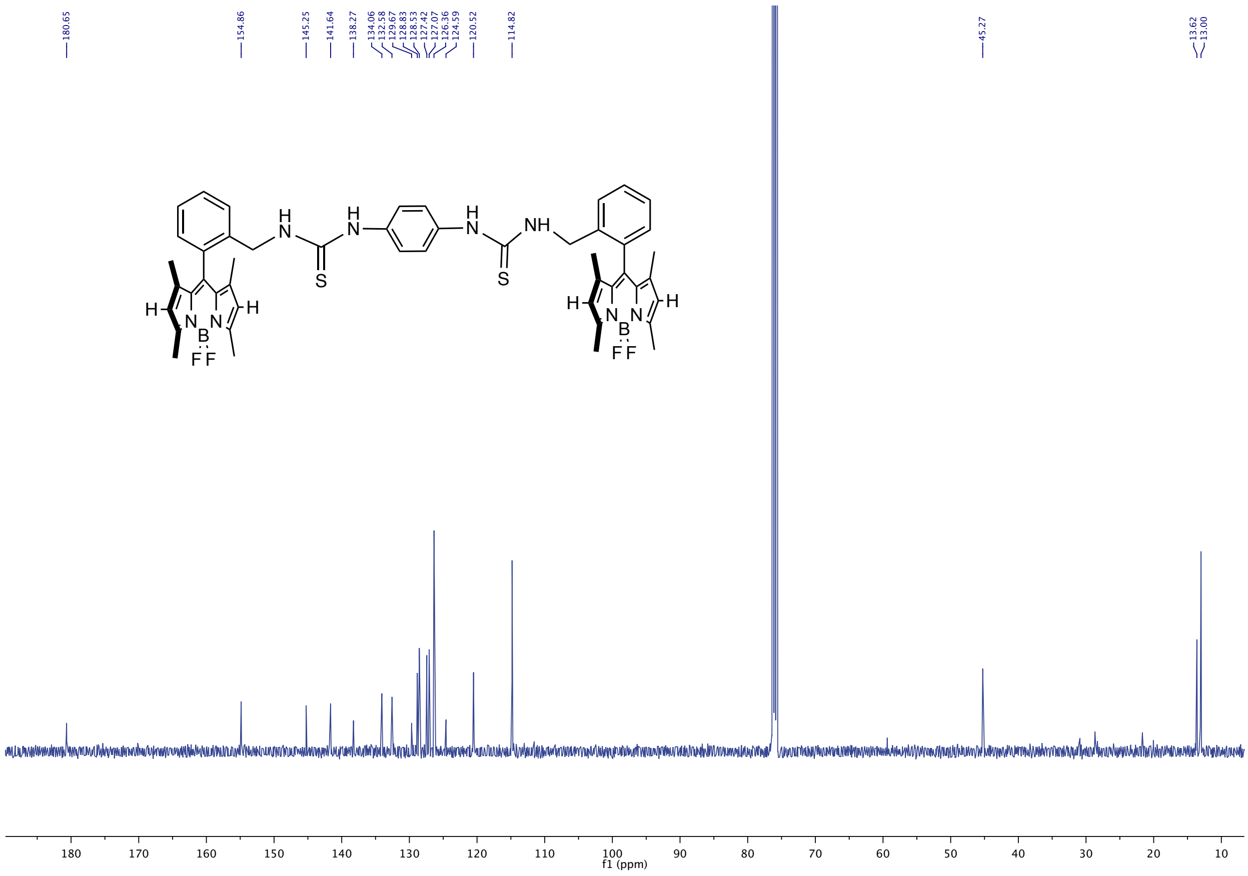

Figure S49. ^{13}C NMR spectrum of compound **5c**

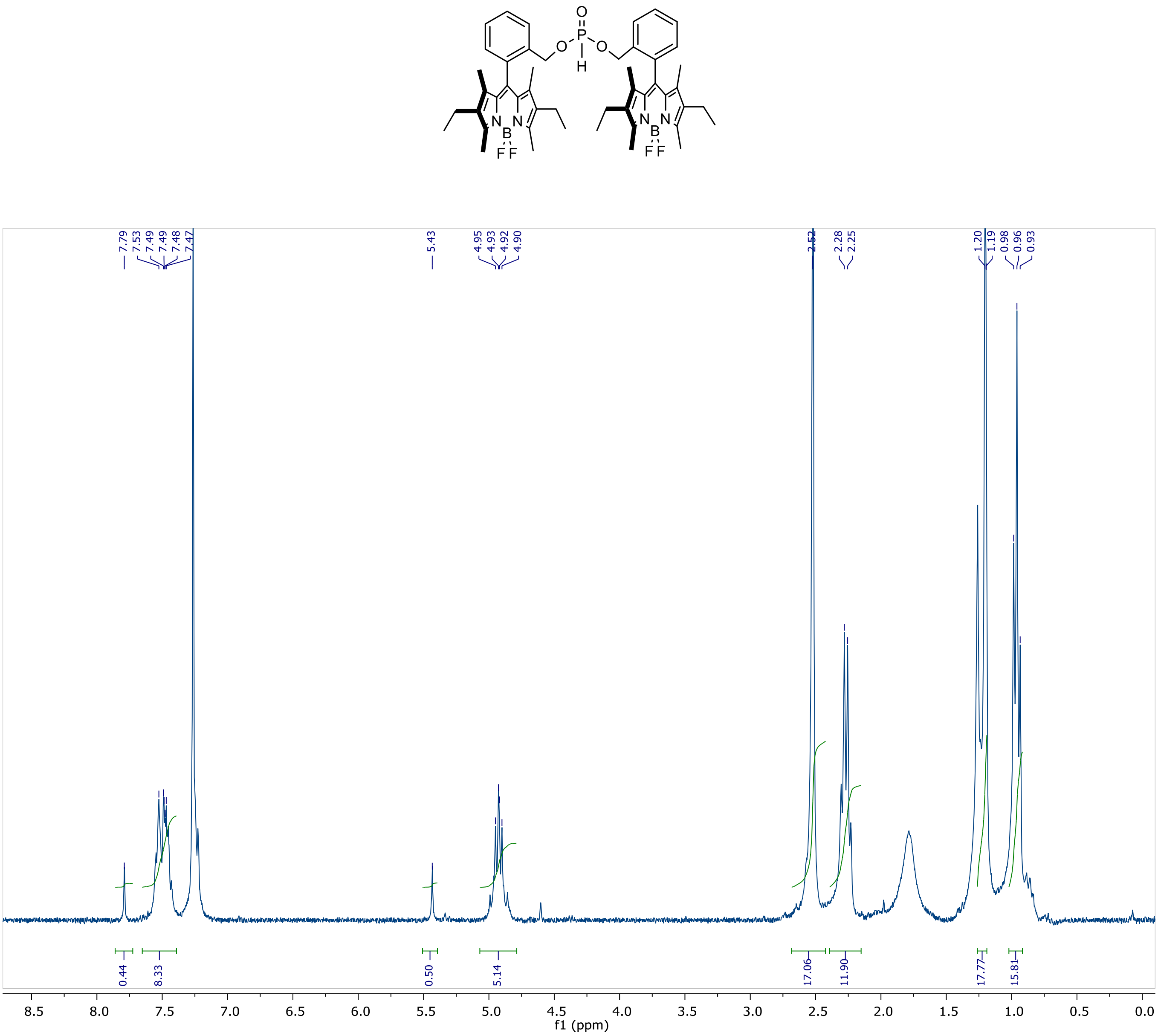

Figure S50. ^{1}H NMR spectrum of compound **5d**

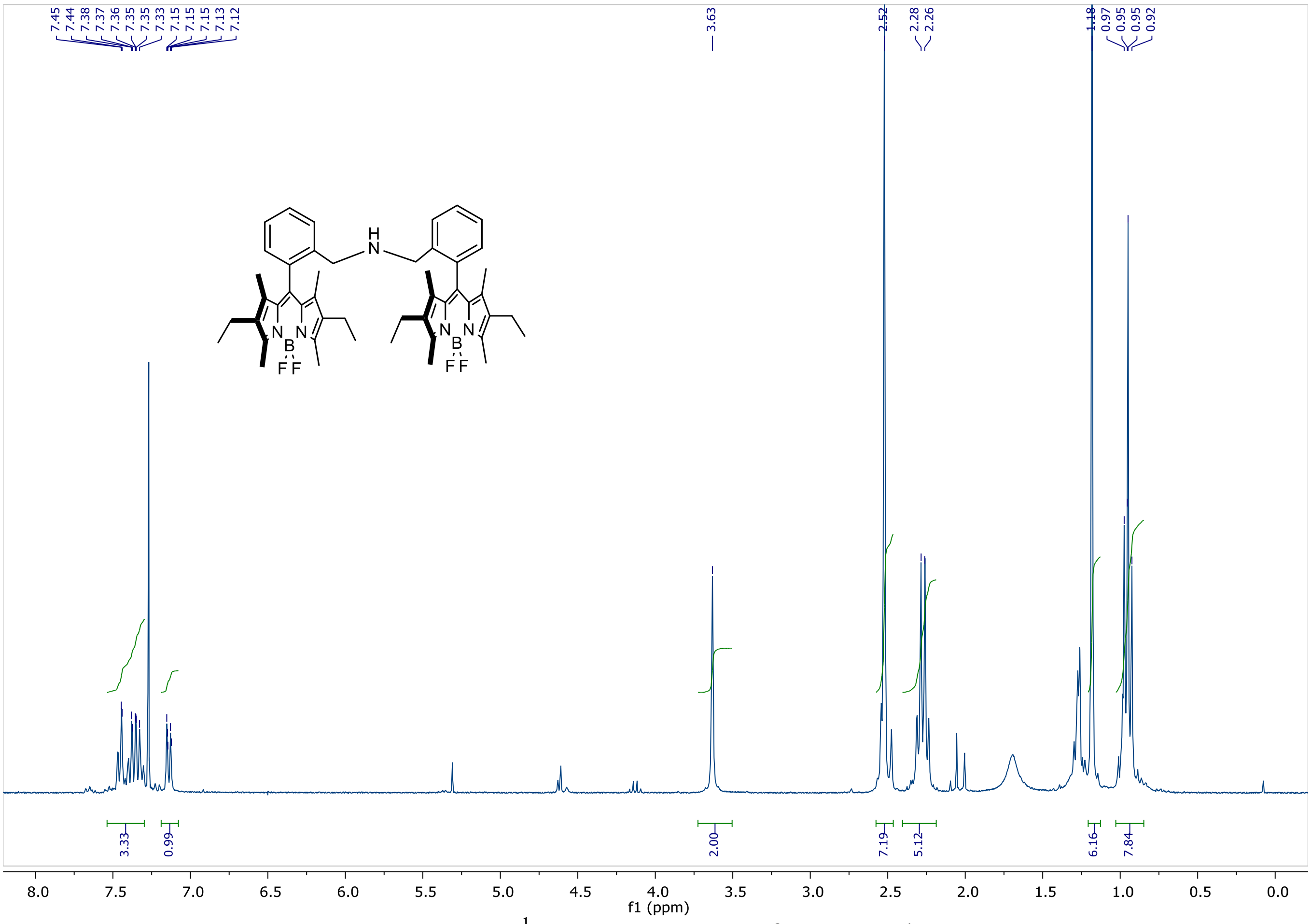

Figure S51. ^{1}H NMR spectrum of compound **5f**

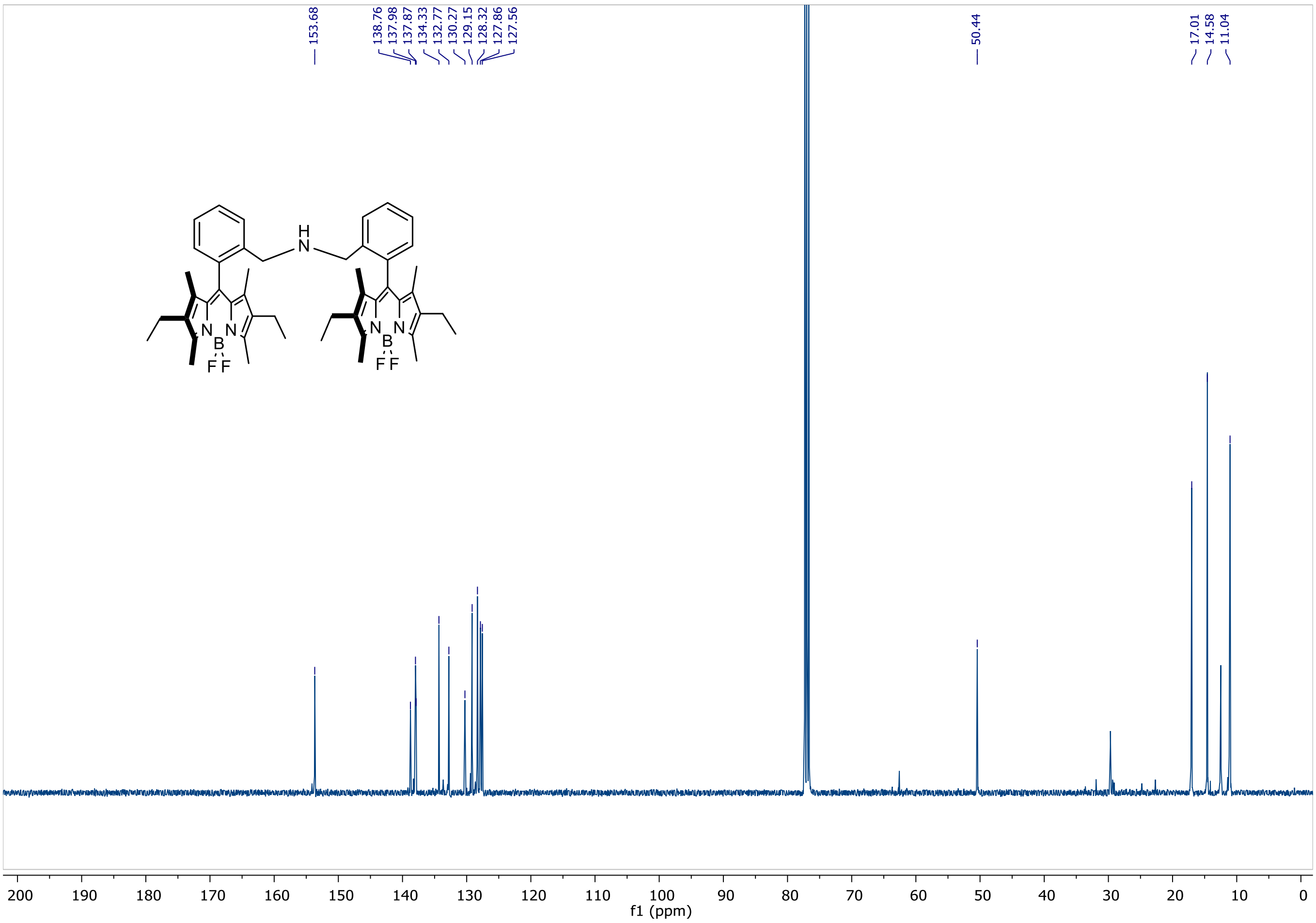

Figure S52. ^{13}C NMR spectrum of compound **5f**

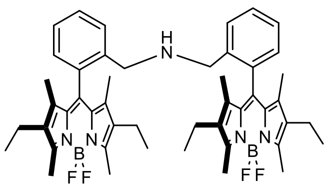

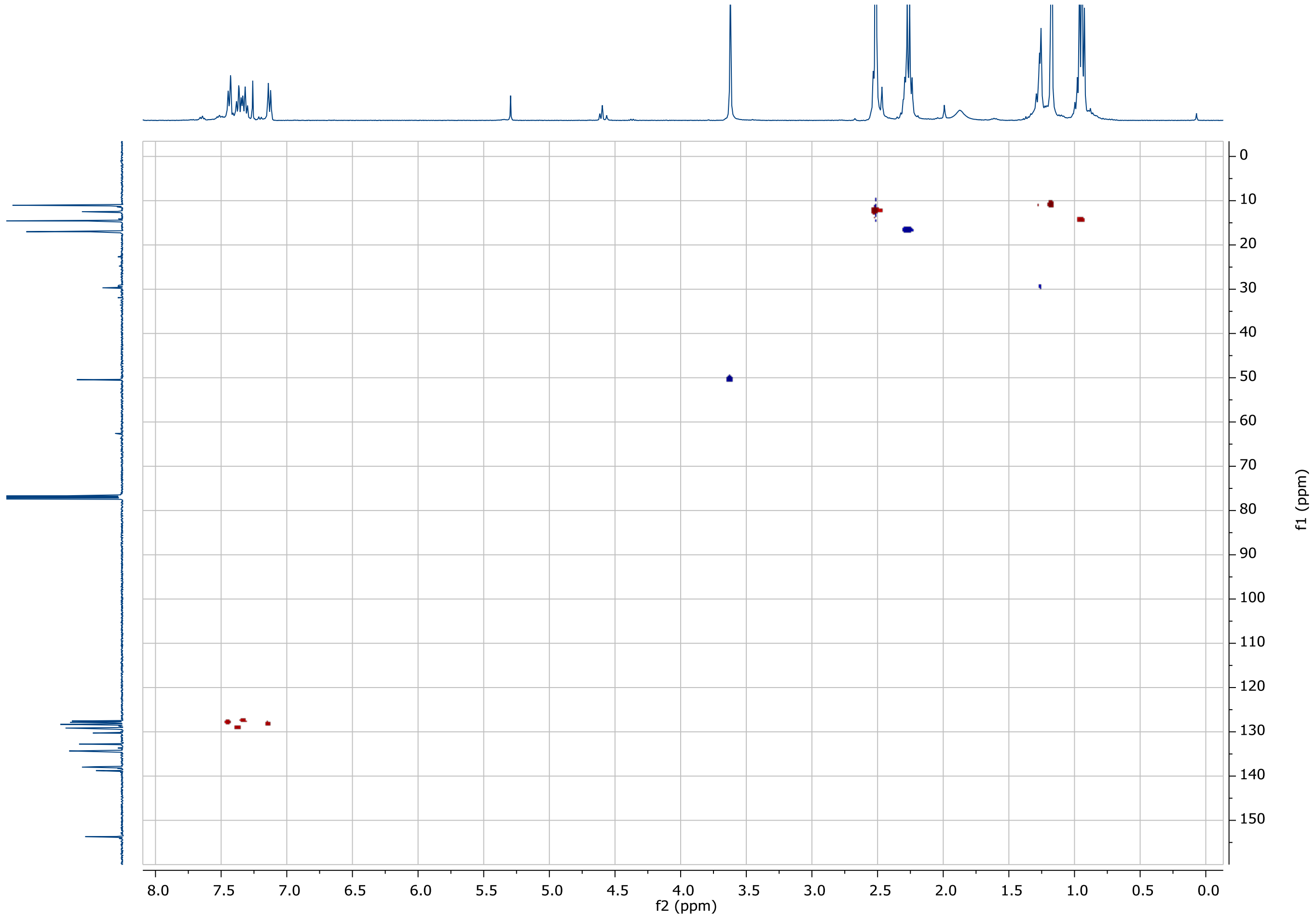

Figure S53. HSQC NMR spectrum of compound **5f**

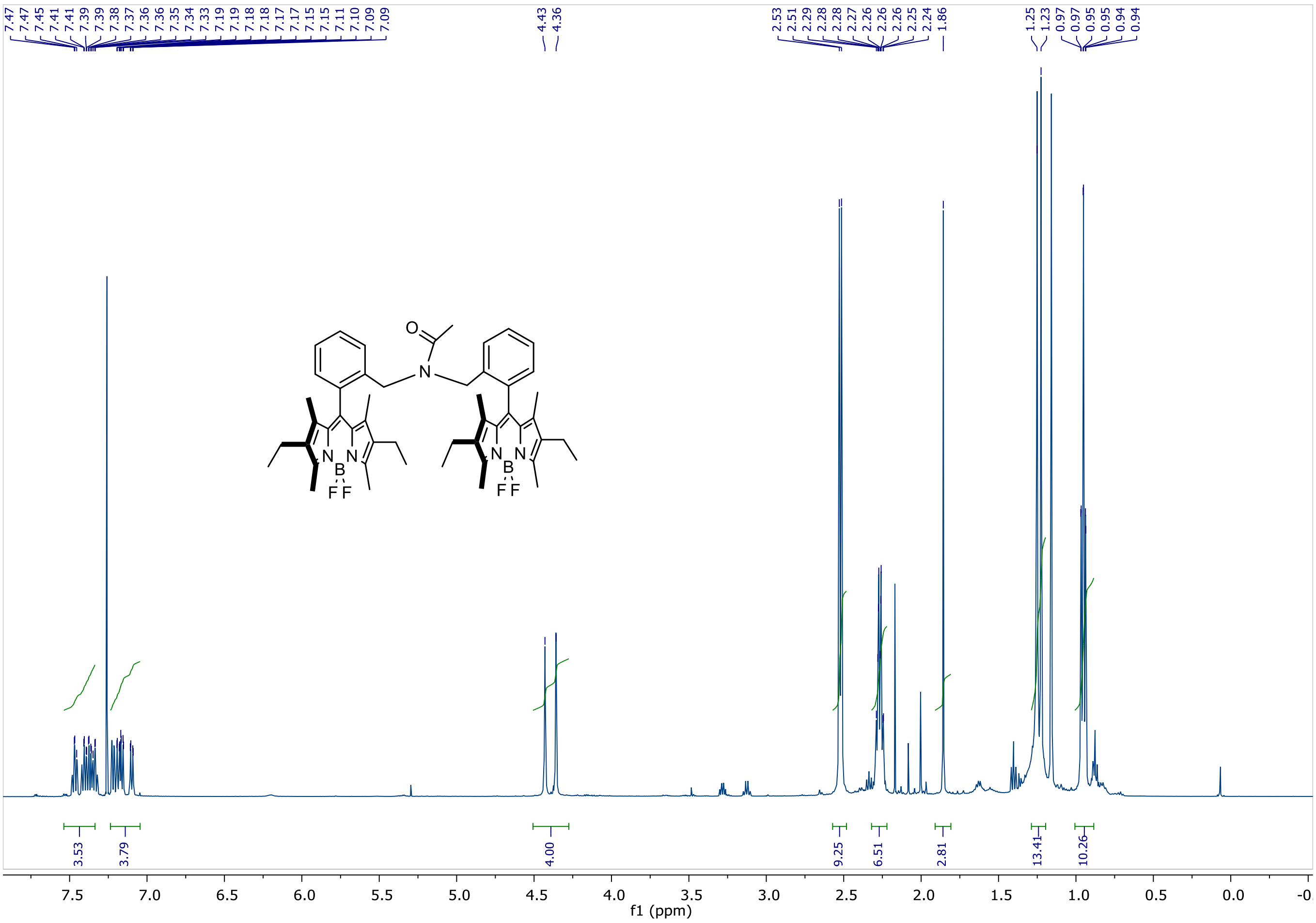

Figure S54. ^{1}H NMR spectrum of compound **5g**

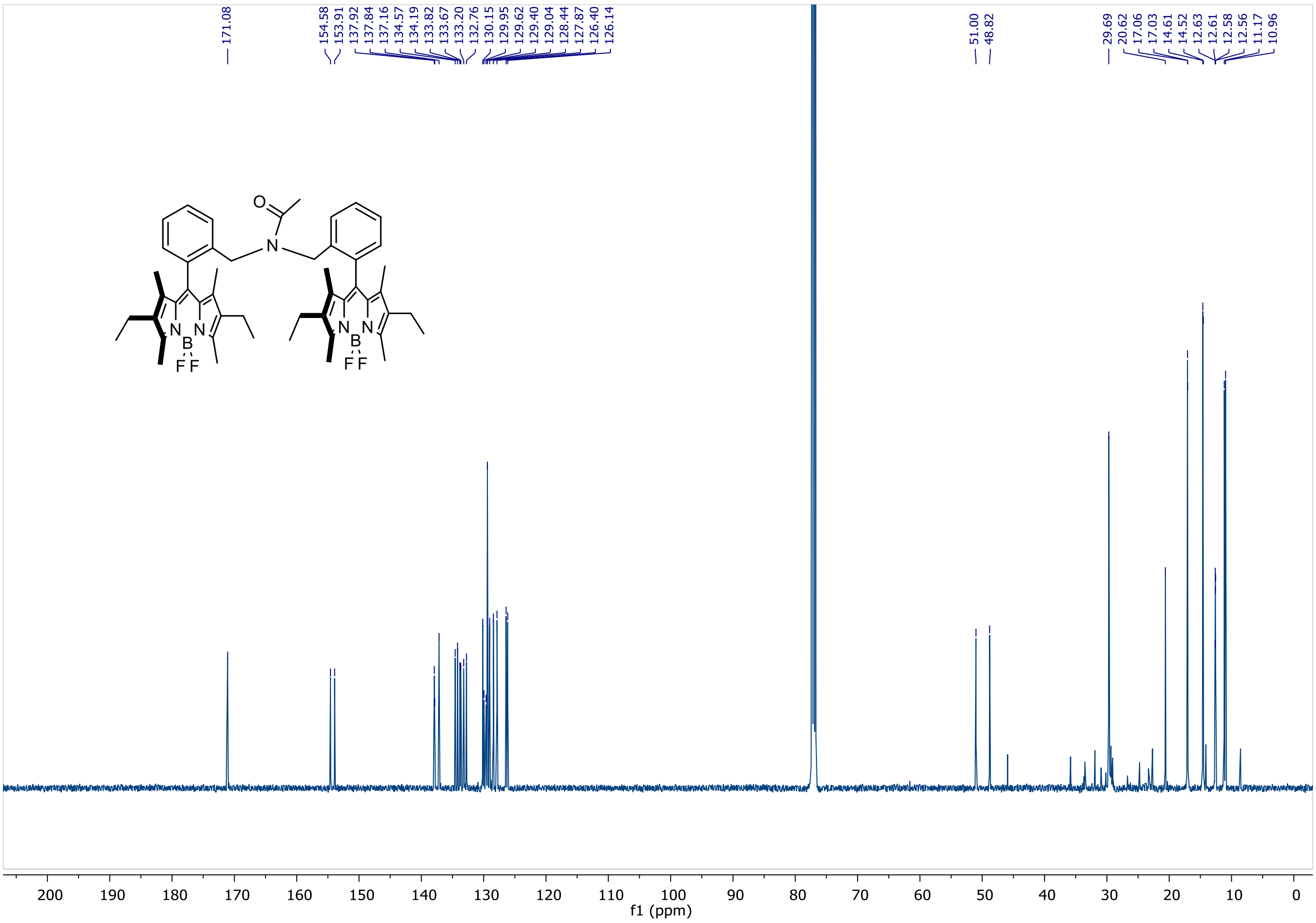

Figure **S55.** ^{13}C NMR spectrum of compound **5g**

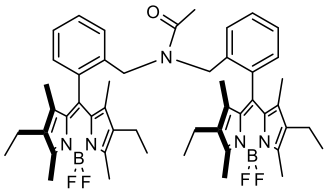

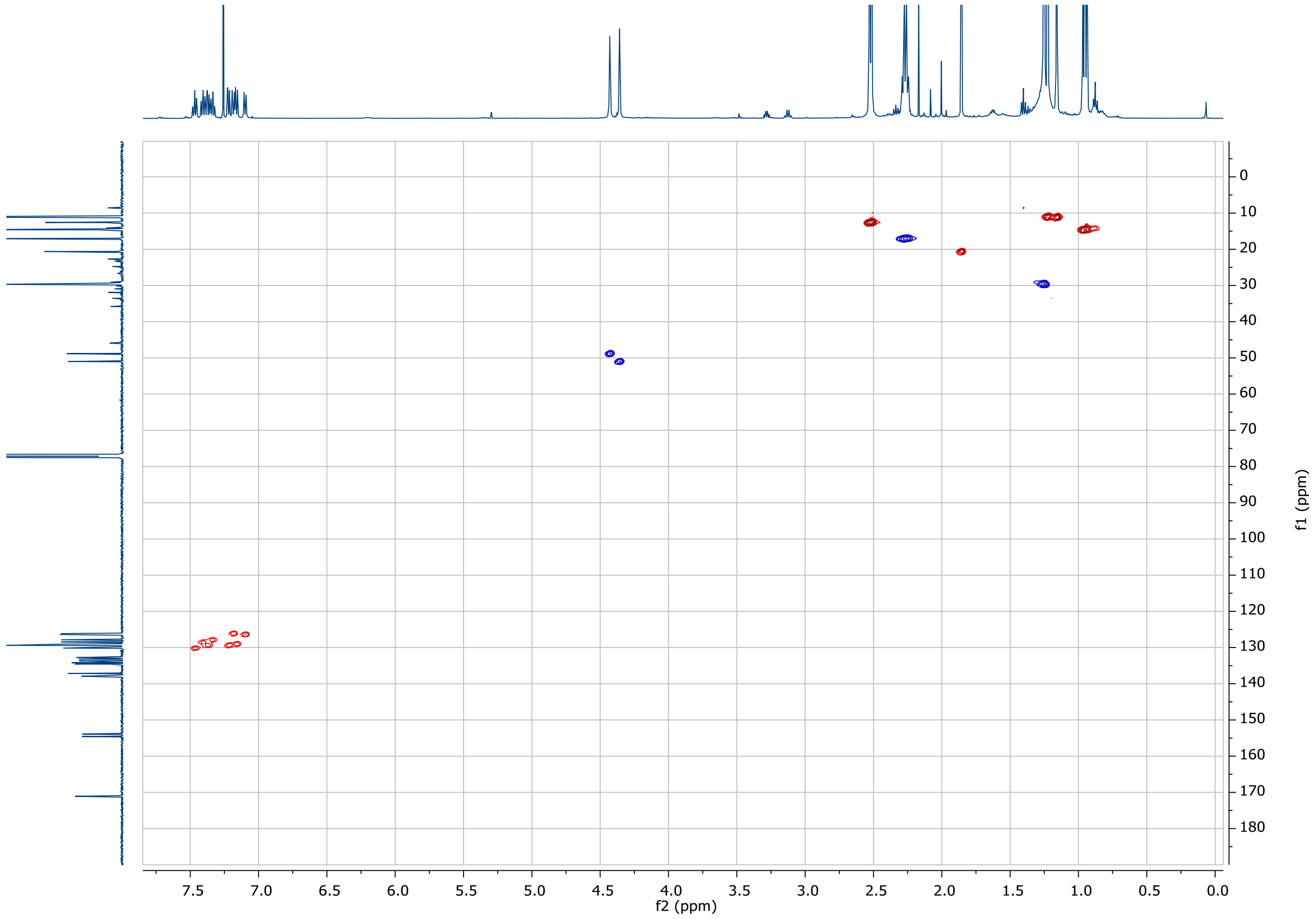

Figure S56. HSQC NMR spectrum of compound **5g**

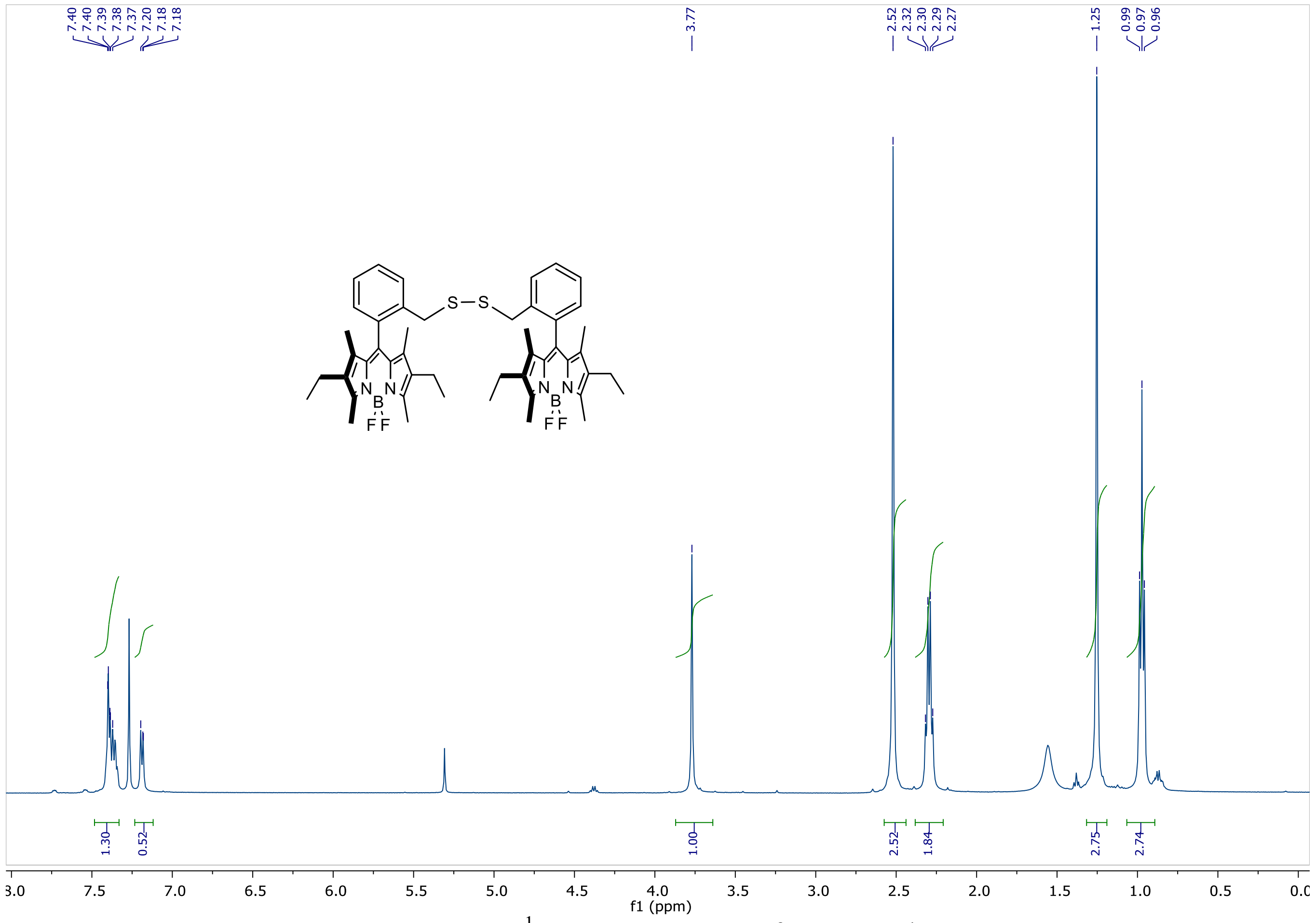

Figure S57. ^{1}H NMR spectrum of compound **5i**

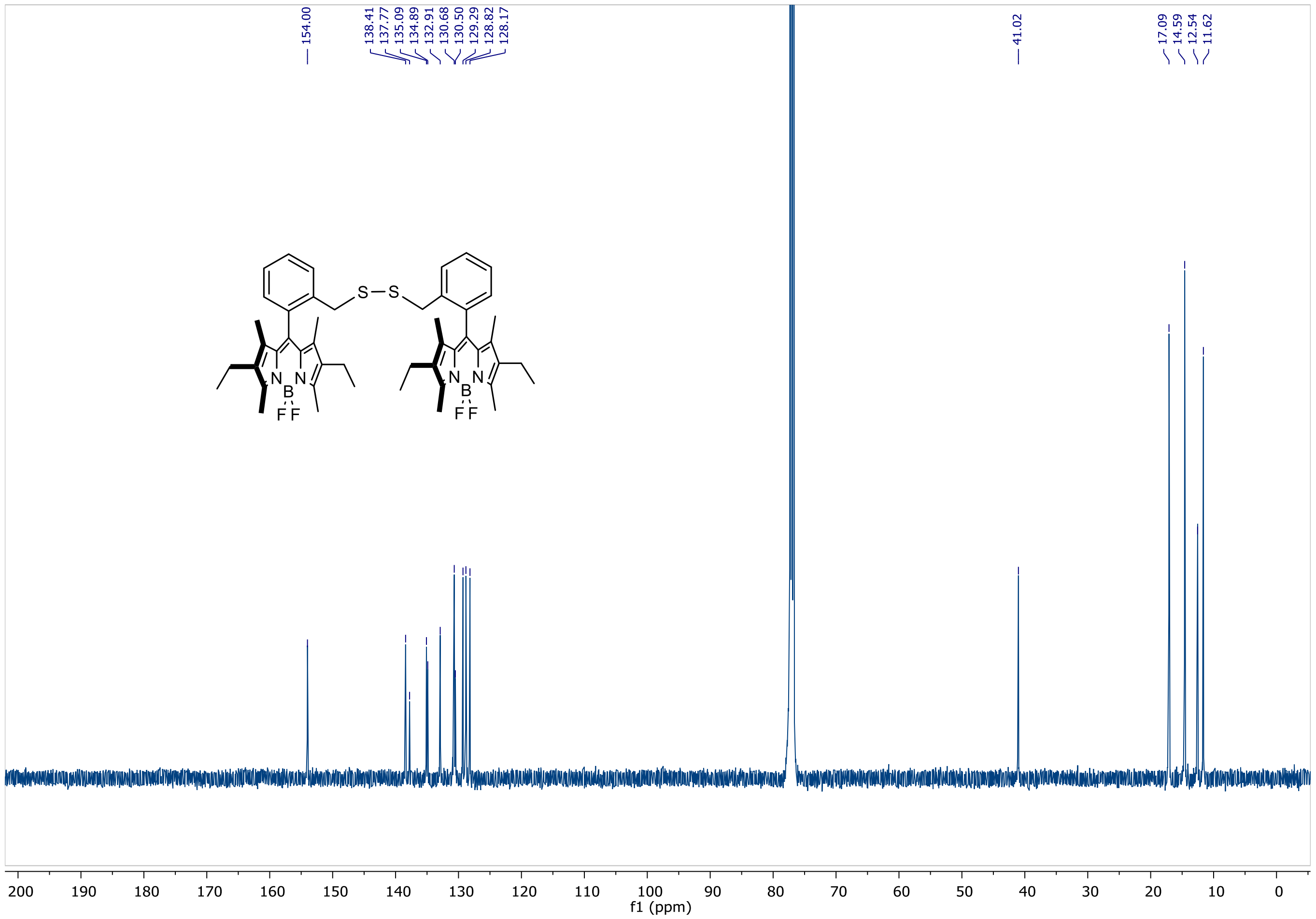

Figure S58. ^{13}C NMR spectrum of compound **5i**

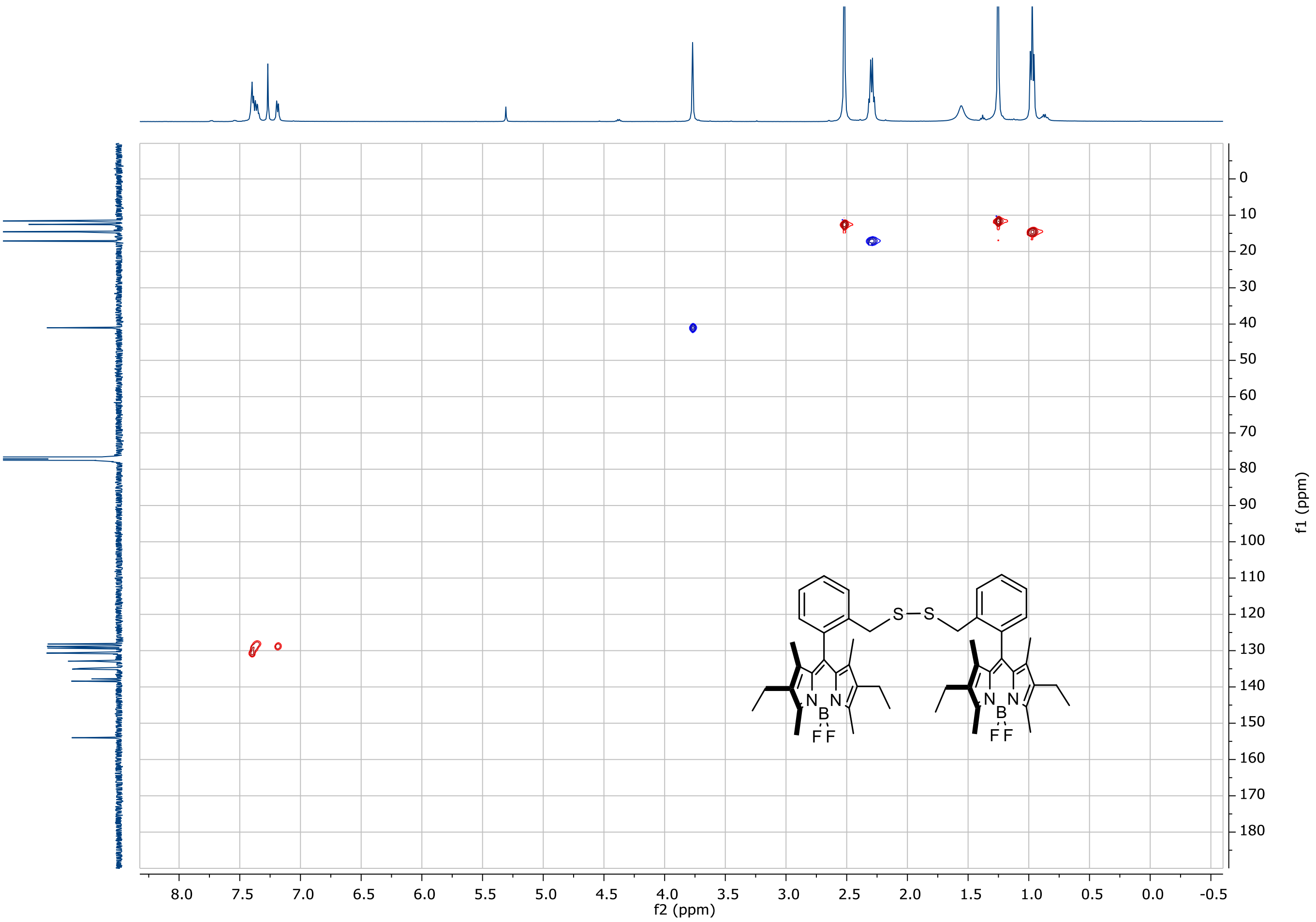

Figure S59. HSQC NMR spectrum of compound **5i**

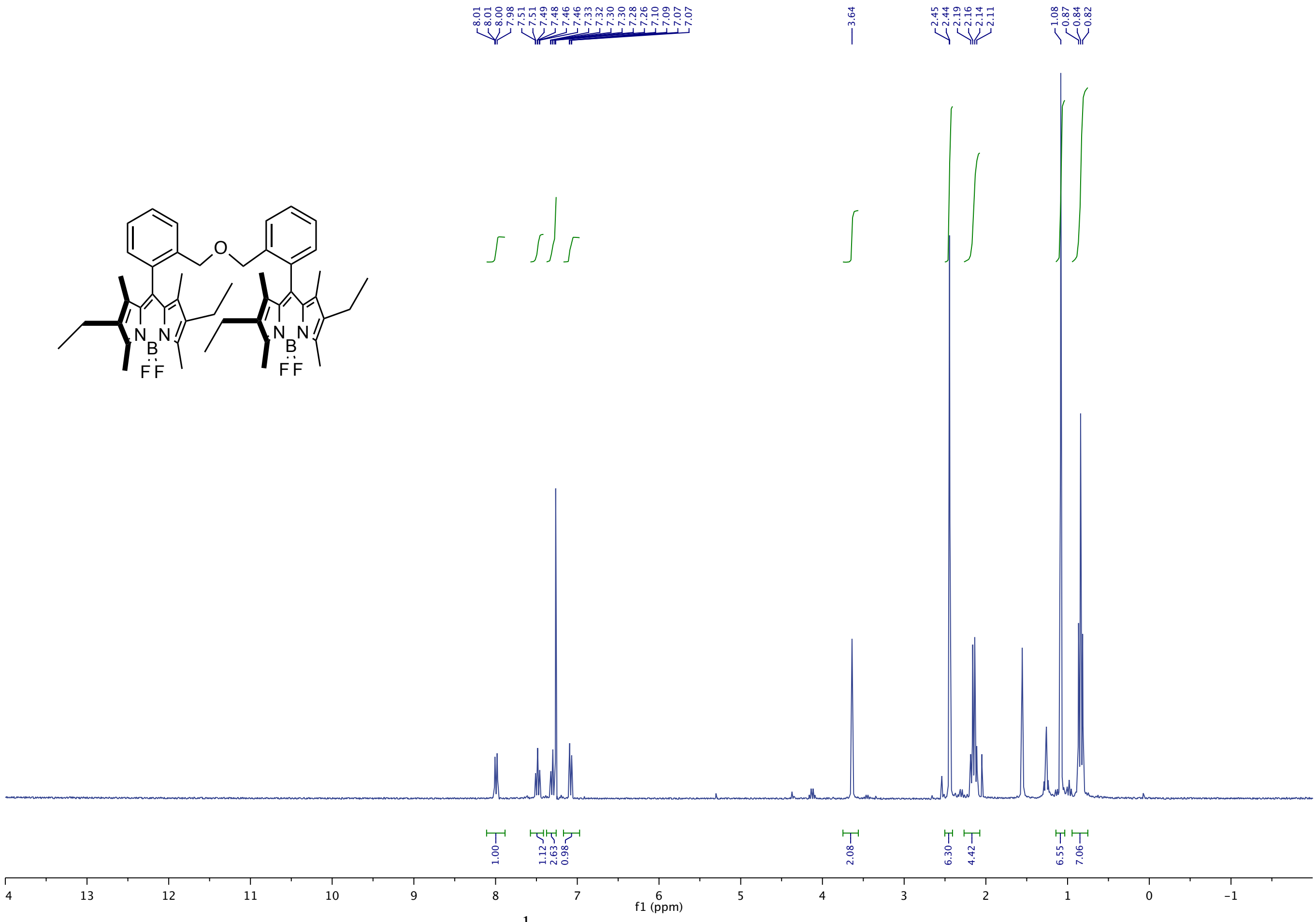

Figure S60. ^{1}H NMR spectrum of compound **5k**

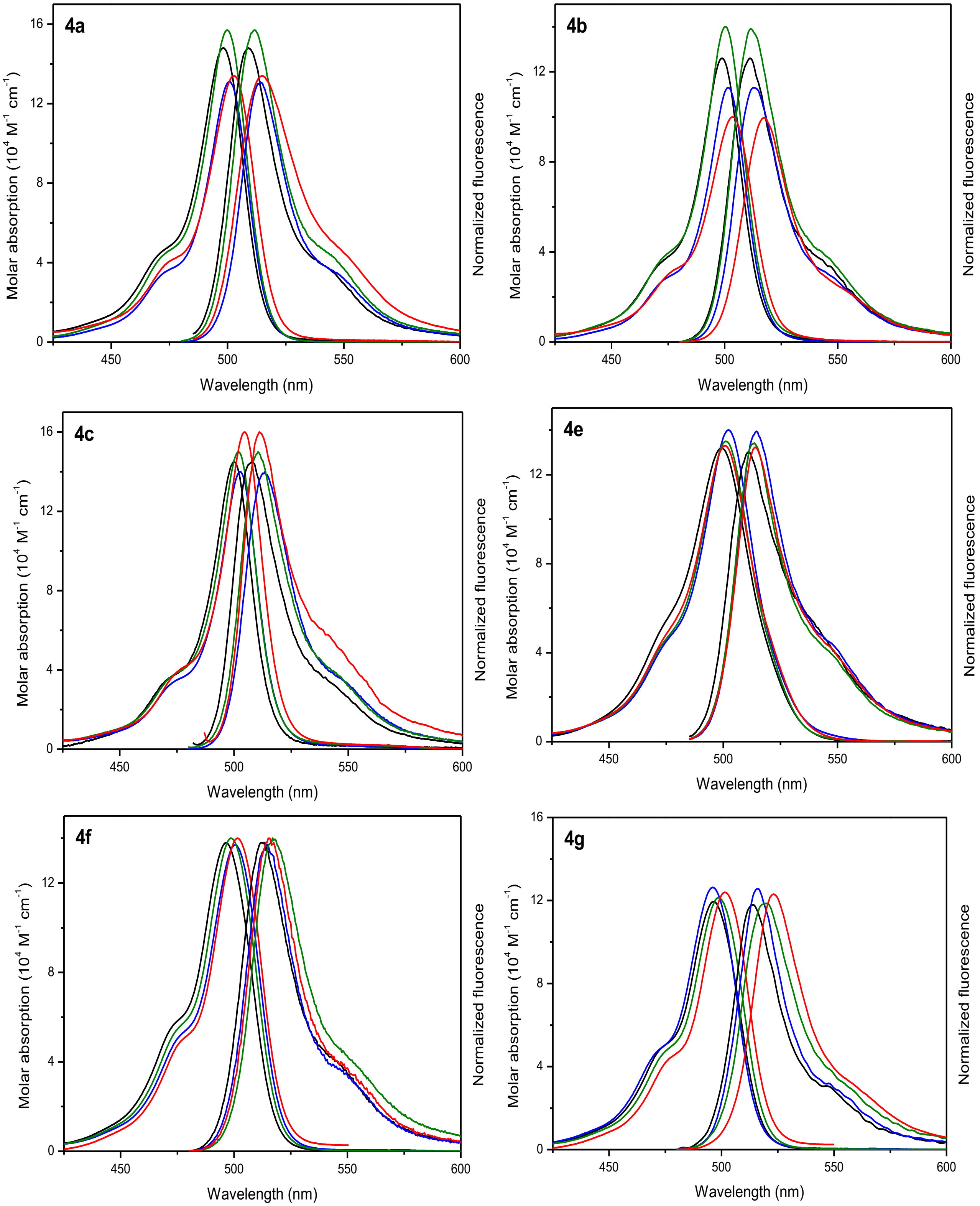
4a
4b
4c
4e
4f
4g
Molar absorption (10^4 M^-1 cm^-1)
Normalized fluorescence
Wavelength (nm)
0
4
8
12
16
450
500
550
600

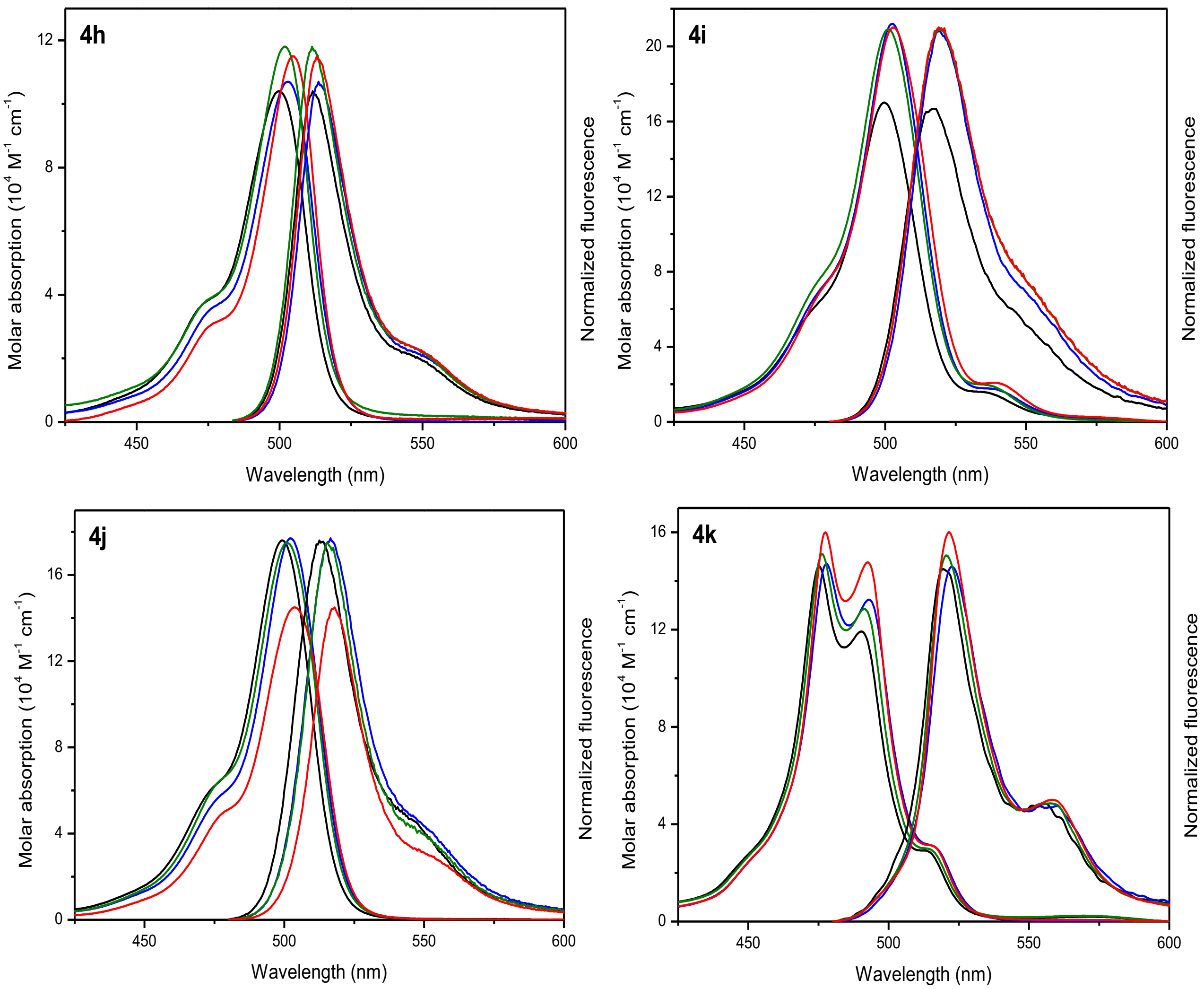

Figure S61. Absorption (scaled by their molar absorption coefficient) and normalized fluorescence spectra properties of the bis-BODIPYs based on tetramethylated dipyrrin building block **2**, bearing different bridges; urea (**4a**), thiourea (**4b** and **4c**), phosphonate (**4e**), amine (**4f**), acetylamine (**4g**), ammonium (**4h**), disulfur (**4i** and **4j**) and ether (**4k**) as spacers, and at different solvents; cyclohexane in red (except **4e** and **4i**, which are in diethyl ether owing to lack of solubility in cyclohexane), ethanol in green, dimethylformamide in blue and acetonitrile in black.

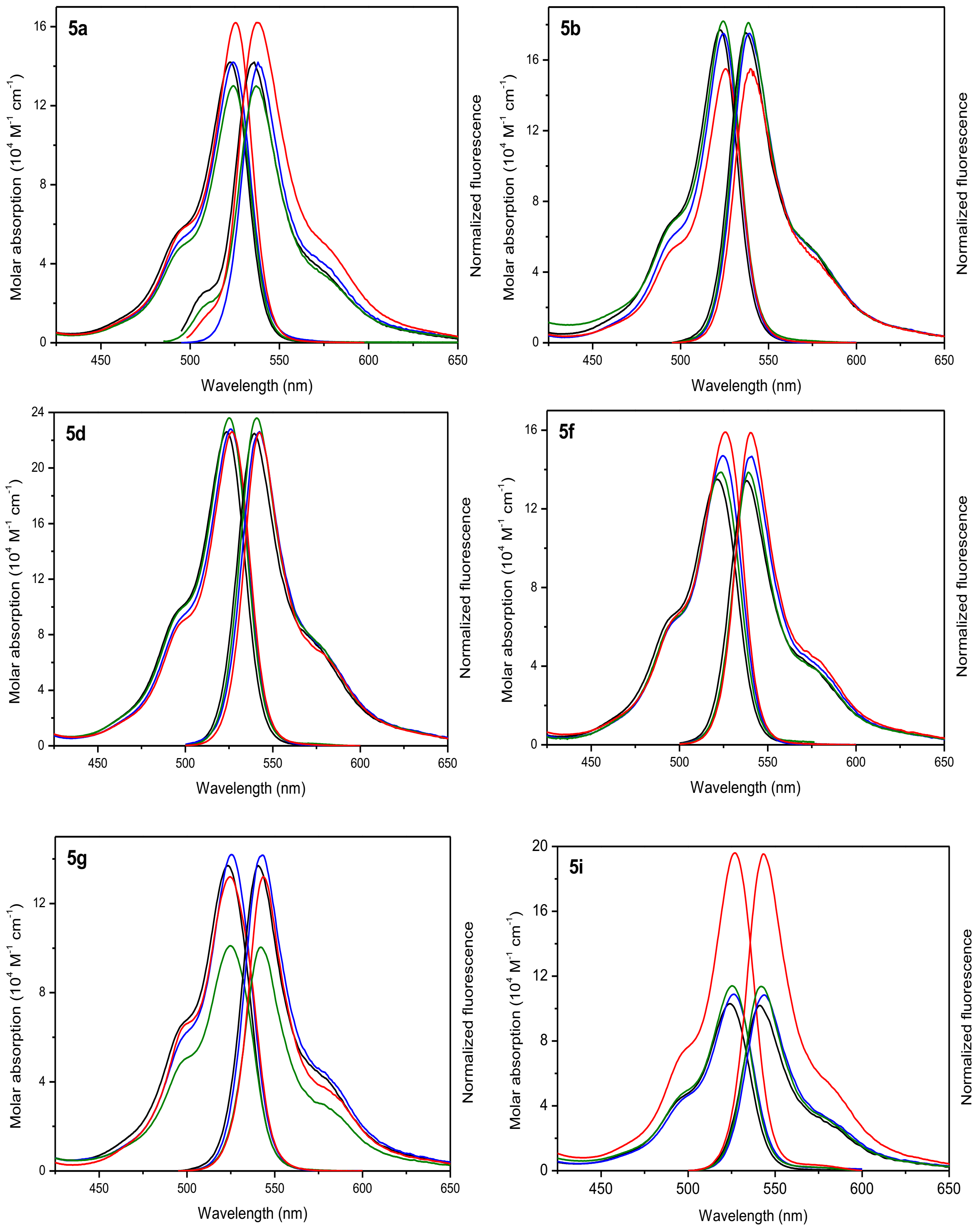
5a
5b
5d
5f
5g
5i
Molar absorption (10^4 M^-1 cm^-1)
Normalized fluorescence
Wavelength (nm)
450
500
550
600
650

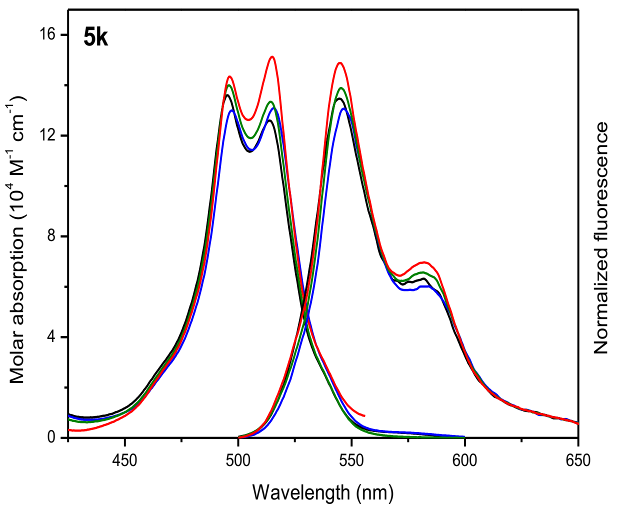

Figure 62. Absorption (scaled by their molar absorption coefficient) and normalized fluorescence spectra properties of the bis-BODIPYs based on fully alkylated dipyrrin building blocks **3**, bearing different bridges; urea (**5a**), thiourea (**5b**), phosphonate (**5d**), amine (**5f**), acetylamine (**5g**), disulfur (**5i**) and and ether (**5k**) as spacers, and at different solvents; cyclohexane in red, ethanol in green, dimethylformamide in blue and acetonitrile in black
